# Open-label randomized controlled trial of ultra-low tidal ventilation without extracorporeal circulation in patients with COVID-19 pneumonia and moderate to severe ARDS: study protocol for the VT4COVID trial

**DOI:** 10.1186/s13063-021-05665-z

**Published:** 2021-10-11

**Authors:** Jean-Christophe Richard, Hodane Yonis, Laurent Bitker, Sylvain Roche, Florent Wallet, Claire Dupuis, Hassan Serrier, Laurent Argaud, Guillaume Thiery, Bertrand Delannoy, Christian Pommier, Paul Abraham, Michel Muller, Frederic Aubrun, Florian Sigaud, Guillaume Rigault, Emilie Joffredo, Mehdi Mezidi, Nicolas Terzi, Muriel Rabilloud

**Affiliations:** 1 Université Lyon 1, Université de Lyon, Lyon, France; 2 Medical Intensive Care Unit, Croix-Rousse Hospital, Hospices Civils de Lyon, 103 Grande Rue de la Croix Rousse, 69004 Lyon, France; 3 CREATIS INSERM 1044 CNRS 5220, Villeurbanne, France; 4 Pôle Santé Publique, Service de Biostatistique et Bioinformatique, Hospices Civils de Lyon, Lyon, France; 5 CNRS, UMR 5558, Laboratoire de Biométrie et Biologie Évolutive, Équipe Biostatistique-Santé, Villeurbanne, France; 6 Medical-Surgical Intensive Care Unit, Lyon-Sud Hospital, Hospices Civils de Lyon, Pierre-Bénite, France; 7 International Center of Research in Infectiology, INSERM U1111, CNRS UMR 5308, ENS, UCBL, Lyon University, Lyon, France; 8 Medical Intensive Care Unit, CHU Gabriel Montpied, Clermont-Ferrand, France; 9 Cellule Innovation, Délégation à la Recherche Clinique et à l’Innovation, Hospices Civils de Lyon, Lyon, France; 10 Medical Intensive Care Unit, Edouard Herriot Hospital, Hospices Civils de Lyon, Lyon, France; 11 Medical Intensive Care Unit, Hopital Nord, CHU Saint-Etienne, Saint-Priest En Jarez, France; 12 Medical-Surgical Intensive Care Unit, Clinique de la Sauvegarde, Lyon, France; 13 Medical-Surgical Intensive Care Unit, Centre Hospitalier Saint Joseph-Saint Luc, Lyon, France; 14 Surgical Intensive Care Unit, Edouard Herriot Hospital, Hospices Civils de Lyon, Lyon, France; 15 Medical-Surgical Intensive Care Unit, Centre Hospitalier Annecy Genevois, Pringy, France; 16 Surgical Intensive Care Unit, Croix-Rousse Hospital, Hospices Civils de Lyon, Lyon, France; 17 Service de Médecine Intensive Réanimation, CHU Grenoble Alpes, La Tronche, France; 18 Université de Grenoble-Alpes, Grenoble, France; 19 INSERM U1042, Grenoble, France

**Keywords:** COVID-19, SARS-CoV-2, Acute respiratory distress syndrome, Mechanical ventilation, Ultraprotective ventilation, Ultra-low tidal volume ventilation, Tidal volume

## Abstract

**Background:**

Acute respiratory distress syndrome (ARDS) is a severe complication of COVID-19 pneumonia, with a mortality rate amounting to 34–50% in moderate and severe ARDS, and is associated with prolonged duration of invasive mechanical ventilation. Such as in non-COVID ARDS, harmful mechanical ventilation settings might be associated with worse outcomes. Reducing the tidal volume down to 4 mL kg^−1^ of predicted body weight (PBW) to provide ultra-low tidal volume ventilation (ULTV) is an appealing technique to minimize ventilator-inducted lung injury. Furthermore, in the context of a worldwide pandemic, it does not require any additional material and consumables and may be applied in low- to middle-income countries. We hypothesized that ULTV without extracorporeal circulation is a credible option to reduce COVID-19-related ARDS mortality and duration of mechanical ventilation.

**Methods:**

The VT4COVID study is a randomized, multi-centric prospective open-labeled, controlled superiority trial. Adult patients admitted in the intensive care unit with COVID-19-related mild to severe ARDS defined by a PaO_2_/FiO_2_ ratio ≤ 150 mmHg under invasive mechanical ventilation for less than 48 h, and consent to participate to the study will be eligible. Patients will be randomized into two balanced parallels groups, at a 1:1 ratio. The control group will be ventilated with protective ventilation settings (tidal volume 6 mL kg^−1^ PBW), and the intervention group will be ventilated with ULTV (tidal volume 4 mL kg^−1^ PBW). The primary outcome is a composite score based on 90-day all-cause mortality as a prioritized criterion and the number of ventilator-free days at day 60 after inclusion. The randomization list will be stratified by site of recruitment and generated using random blocks of sizes 4 and 6. Data will be analyzed using intention-to-treat principles.

**Discussion:**

The purpose of this manuscript is to provide primary publication of study protocol to prevent selective reporting of outcomes, data-driven analysis, and to increase transparency. Enrollment of patients in the study is ongoing.

**Trial registration:**

ClinicalTrials.govNCT04349618. Registered on April 16, 2020

**Supplementary Information:**

The online version contains supplementary material available at 10.1186/s13063-021-05665-z.

## Administrative information

Note: the numbers in curly brackets in this protocol refer to SPIRIT checklist item numbers. The order of the items has been modified to group similar items (see http://www.equator-network.org/reporting-guidelines/spirit-2013-statement-defining-standard-protocol-items-for-clinical-trials/).

**Table:** 

Title {1}	Open label randomized controlled trial of ultra-low tidal ventilation without extracorporeal circulation in patients with COVID-19 pneumonia and moderate to severe ARDS. Study protocol for the VT4COVID trial.
Trial registration {2a and 2b}.	ClinicalTrials.gov, NCT04349618
Protocol version {3}	April 12th, 2020 -version 3 January 22th, 2021- version 4
Funding {4}	French Ministry of Health (PHRCI 2020) and Hospices Civils de Lyon
Author details {5a}	Jean-Christophe RICHARD^1,2,3^, Hodane YONIS^2^, Laurent BITKER^1,2,3^, Sylvain ROCHE^1,4,5^, Florent WALLET^6,7^, Claire DUPUIS^8^, Hassan SERRIER^9^, Laurent ARGAUD^10^, Guillaume THIERY^11^, Bertrand DELANNOY^12^, Christian POMMIER^13^, Paul ABRAHAM^14^, Michel MULLER^15^, Frederic AUBRUN^16^, Florian SIGAUD^17^, Guillaume RIGAULT^17,18^, Emilie JOFFREDO^6^, Mehdi MEZIDI^1,2^, Nicolas TERZI^17,18,19^, Muriel RABILLOUD^1,4,5^ 1. Université de Lyon, Université Lyon 1, Lyon, FRANCE 2. Hospices Civils de Lyon, Croix-Rousse Hospital, Medical Intensive Care Unit, Lyon, FRANCE 3. CREATIS INSERM 1044 CNRS 5220, Villeurbanne, FRANCE 4. Hospices Civils de Lyon, Pôle Santé Publique, Service de Biostatistique et Bioinformatique, Lyon, FRANCE 5. CNRS, UMR 5558, Laboratoire de Biométrie et Biologie Évolutive, Équipe Biostatistique-Santé, Villeurbanne, FRANCE. 6. Hospices Civils de Lyon, Lyon-Sud Hospital, Medical-Surgical Intensive Care Unit, Pierre-Bénite, FRANCE 7. International Center of Research in Infectiology, Lyon University, INSERM U1111, CNRS UMR 5308, ENS, UCBL, Lyon, FRANCE 8. CHU Gabriel Montpied, Medical Intensive Care Unit, Clermont-Ferrand, FRANCE 9. Hospices Civils de Lyon, Cellule Innovation, Délégation à la Recherche Clinique et à l’Innovation, Lyon, FRANCE. 10. Hospices Civils de Lyon, Edouard Herriot Hospital, Medical Intensive Care Unit, Lyon, FRANCE 11. CHU Saint-Etienne, Hopital Nord, Medical Intensive Care Unit, Saint-Priest-En-Jarez, FRANCE 12. Clinique de la Sauvegarde, Medical-Surgical Intensive Care Unit, Lyon, FRANCE 13. Centre Hospitalier Saint Joseph-Saint Luc, Medical-Surgical Intensive Care Unit, Lyon, FRANCE 14. Hospices Civils de Lyon, Edouard Herriot Hospital, Surgical Intensive Care Unit, Lyon, FRANCE 15. Centre Hospitalier Annecy Genevois, Medical-Surgical Intensive Care Unit, Pringy, FRANCE 16. Hospices Civils de Lyon, Croix-Rousse Hospital, Surgical Intensive Care Unit, Lyon, FRANCE 17. CHU Grenoble Alpes, Service de Médecine Intensive Réanimation, La Tronche, FRANCE 18. Université de Grenoble-Alpes, Grenoble, FRANCE 19. INSERM U1042, Grenoble, FRANCE
Name and contact information for the trial sponsor {5b}	[SPIRIT guidance: Name and contact information for the trial sponsor.] Hospices Civils de Lyon A PACHOT. Direction de la Recherche Clinique. Hospices Civils de Lyon. Lyon, FRANCE
Role of sponsor {5c}	The study funder had no part in study design; collection, management, analysis, and interpretation of data; writing of the report; and in the decision to submit the report for publication. The study funder had no ultimate authority over any of these activities The study sponsor had no part in study design, collection, analysis, and interpretation of data; writing of the report; and in the decision to submit the report for publication, but was involved in data management. The study sponsor had no ultimate authority over any of these activities

## Introduction

### Background and rationale {6a}

Acute respiratory distress syndrome (ARDS) is the most severe complication related to COVID-19 pneumonia and was observed in 78% of the patients under mechanical ventilation in one of the largest prospective case series of COVID-19 patients who required intensive care admission [1]. Current management of COVID-19-related ARDS is based on mechanical ventilation in intensive care units until recovery of lung injury and dexamethasone [2]. However, COVID-19-related 90-day mortality is extremely high, amounting to 34% and 50% in moderate and severe ARDS, respectively [1]. Harmful mechanical ventilation settings might partly explain the mortality of COVID-19 pneumonia. Indeed, 30% of ARDS patients under protective ventilation exhibit tidal hyperinflation on computed tomography, associated with an increase in pro-inflammatory mediators in broncho-alveolar lavage (BAL) [3], suggesting excessive tidal volume (VT). The magnitude of tidal hyperinflation may be even greater in COVID-19 ARDS patients, as we recently observed in a CT study on 13 COVID-19 ARDS patients as compared to 9 non-COVID-19 ARDS patients [4]. Moreover, in a recent observational study on 482 ARDS patients, an increase of 1 mL kg^−1^ predicted body weight (PBW) in initial VT was associated with a 23% increase in adjusted mortality risk, suggesting that small variations in VT at the initial phase of ARDS may impact prognostic [5]. Reducing VT from 6 to 3–4 mL kg^−1^ PBW (so-called ultraprotective ventilation) combined with extracorporeal CO_2_ removal (ECCOR) was associated with a decrease in pro-inflammatory mediators in BAL [6] and a lower mortality in the more severe patients [7].

We have recently performed a pilot multicenter before-after trial [8], on 34 ARDS patients, aiming to provide ultra-low tidal volume ventilation (ULTV) without ECCOR, while aiming to keep arterial pH above 7.20. In this study, VT could be reduced to 4 mL kg^−1^ PBW in 65% and to 5 mL kg^−1^ PBW in 88% of the patients, although 9% of the patients had a pH below 7.20 at inclusion. This strategy was applied during the whole course of ARDS, sedation dose was not significantly increased, and the rate of acute cor pulmonale (6%) was lower than expected. Regarding safety, day 90 mortality amounted to 41% despite the inclusion of some moribund patients, and 32% of the patients developed transient severe acidosis with pH < 7.15.

ULTV without extracorporeal circulation is an appealing technique to protect the ARDS lung in the context of a worldwide pandemic, as it does not require any material and consumables, and may be applied in low- to middle-income countries. We hypothesized that ULTV without extracorporeal circulation is a credible option to reduce COVID-19 mortality and duration of mechanical ventilation, in patients with severe and moderately severe ARDS [2].

### Objectives {7}

The main objective is to assess the benefit of ULTV in comparison with protective ventilation on a composite primary endpoint that will include the all-cause mortality at day 90 as the prioritized criterion and the number of days free from invasive mechanical ventilation (VFD) at day 60 as the second criterion.

The following are the secondary objectives:

1. To test whether ULTV is associated with a decrease in ARDS all-cause mortality at day 90 after inclusion
2. To test whether ULTV is associated with higher VFD at day 60 after inclusion
3. To test whether ULTV is associated with a decrease in time to successful extubation
4. To test whether ULTV is associated with a shorter hospital stay
5. To test whether ULTV impacts the following respiratory parameters assessed daily from inclusion to weaning of deep sedation or 14 days after inclusion whichever comes first (PaO_2_/FiO_2_ ratio, pH, PaCO_2_, positive end-expiratory pressure (PEEP), VT, total PEEP, plateau pressure, driving pressure, respiratory rate, mechanical power (MP))
6. To test whether ULTV is associated with an increase in sedation dose during the first 14 days after inclusion
7. To test whether ULTV is associated with an increase in rescue therapy during the first 14 days after inclusion
8. To test whether ULTV is associated with a decrease in the incidence density rate of adverse events during the first 28 days after inclusion (severe mixed acidosis, ventilator-associated pneumonia, acute cor pulmonale, barotrauma, any serious adverse events)
9. To test whether ULTV is associated with cognitive impairment at day 365 after inclusion
10. To test whether ULTV is associated with quality-of-life impairment at day 365 after inclusion
11. To test whether ULTV is associated with post-traumatic stress disorder at day 365 after inclusion
12. To evaluate the efficiency of the innovative strategy performing a cost-effectiveness analysis at day 90 after inclusion

### Trial design {8}

The study is a multicenter prospective open-labeled, randomized controlled superiority trial, with two parallel groups and balanced randomization at a 1:1 ratio.

## Methods: participants, interventions, and outcomes

The third version of the protocol (Additional file 1) was published on April 12, 2020, before the inclusion of the 1st patient in the study. The current version is the fourth version of the protocol (Additional file 2), which was accepted on January 22, 2021, by the research ethics committee. Changes between versions 3 and 4 will be reported below. The WHO Trial Registration Data Set is provided in Additional file 3.

### Study setting {9}

The study will be conducted in 11 intensive care units located in both academic and non-academic French hospitals (Additional file 4).

### Eligibility criteria {10}

#### Inclusion criteria

- Adults aged 18 years or older
- ARDS according to the Berlin Definition [9]
- COVID-19 pneumonia confirmed by a positive SARS-Cov-2 RT-PCR performed less than 7 days before inclusion
- Invasive mechanical ventilation with PaO_2_/FiO_2_ ≤ 150 mmHg and PEEP ≥ 5 cmH_2_O and with a VT ≤ 6 mL kg^−1^ PBW

#### Non-inclusion criteria

1. Exclusion criteria related to ARDS history
  - Onset of invasive or non-invasive ventilation more than 48 h before inclusion
  - Previous inclusion in the present study
2. Exclusion criteria related to ARDS severity or complications
  - Arterial pH < 7.21 despite respiratory rate set to 35 min^−1^ at the time of inclusion
  - Patient under any extracorporeal CO_2_ removal technique or extracorporeal membrane oxygenation
  - Pneumothorax or bronchopleural fistula
3. Exclusion criteria related to comorbidities
  - Suspected intracranial hypertension
  - Severe chronic obstructive pulmonary disease defined by a GOLD score ≥ 3
  - Chronic respiratory failure requiring long term oxygen or non-invasive ventilation
  - Obesity with body weight over height ratio greater than 1 kg cm^−1^
  - Sickle cell disease
  - Bone marrow transplant < 6 months or neutropenia
  - Burn injury with extension greater than 30% of body surface area
  - Cirrhosis with Child-Pugh score C
  - Advance directives to withhold or withdraw life-sustaining treatment
4. Exclusion criteria related to legislation
  - Patient under an exclusion period relative to participation to another clinical trial, current inclusion into another clinical trial sharing the same primary endpoint as the present study, or inclusion into a clinical trial involving unlicensed new drugs or medical devices. Modification of this criterion was accepted by the research ethics committee on January 22, 2021, and co-inclusion into a clinical trial involving unlicensed new drugs or medical devices was no longer forbidden.
  - Pregnancy.
  - Patient under a legal protective measure.
  - Lack of affiliation to social security as required by French regulation.
  - Lack of written informed consent by the patient or next of kin.

### Who will take informed consent? {26a}

Before inclusion in the trial, written informed consent of patient’s the legal representative will be sought by local investigators, as all patients are expected to be unable to consent. If the patients’ legal representative could not be physically present for a written statement (e.g., transport restrictions relative to lockdown or quarantine), an emergency inclusion procedure could be used for inclusion, and study approval by the patients’ legal representative was later sought. In any case, patient’s written informed consent was sought as soon as its medical condition allowed this procedure.

### Additional consent provisions for collection and use of participant data and biological specimens {26b}

Potential future studies intending unplanned use of the data generated in this trial will require an additional consent of included patients. Unplanned use of biological specimens will not be performed.

### Interventions

#### Explanation for the choice of comparators {6b}

The control group will be managed with protective ventilation (i.e., VT 6 mL kg^−1^ PBW, PEEP set using the PEEP-FiO_2_ table of the low PEEP arm of the ALVEOLI trial [10], aiming for a plateau pressure < 30 cmH_2_O), as this strategy reflects a standard of care according to the national [11] and international guidelines [12].

#### Intervention description {11a}

Within the first hour after randomization, the control group will be managed with protective ventilation (i.e., VT 6 mL kg^−1^ PBW, PEEP set using the PEEP-FiO_2_ table of the low PEEP arm of the ALVEOLI trial [10], aiming for a plateau pressure < 30 cmH_2_O) under deep sedation (i.e., Richmond Agitation-Sedation Scale (RASS) score [13] between − 3 and − 4). Respiratory rate (RR) will be set targeting a pH range between 7.20 and 7.45, and VT may be increased if pH remains below 7.15 despite a RR set to 35 min^−1^ and minimization of instrumental dead space (Table 1).

**Table 1:** Summary of the ventilation procedure

• Ventilatory mode: volume-assist control.	
• Instrumental dead space: minimize by using a heated humidifier and a low-volume endotracheal tube connector.	
• Control group: set VT to 6 mL kg^−1^ PBW.	
• Intervention group: stepwise reduction by 1 mL kg^−1^ PBW steps at intervals ≤ 2 h down to 4 mL kg^−1^ PBW and increase RR up to 35 min^−1^ to maintain VE constant.	
• Ratio of the duration of inspiration to the duration of expiration: adjust between 1:2 and 1:4 to maintain intrinsic PEEP ≤ 2 cm H_2_O.	
• Ventilatory goals: plateau pressure ≤ 30 cmH_2_O; 60 ≤ PaO_2_ ≤ 80 mmHg or 88% ≤ SpO_2_ ≤ 95%; 7.20 ≤ pH ≤ 7.45.	
• Allowable combinations of PEEP (cm of H_2_O) and FiO_2_: 5 and 30%, 5 and 40%, 8 and 40%, 8 and 50%, 10 and 50%, 10 and 60%, 10 and 70%, 12 and 70%, 14 and 70%, 14 and 80%, 14 and 90%, 16 and 90%, 18 and 90%, 20 and 100%, 22 and 100%, and 24 and 100%. Other combinations are allowed as a function of hemodynamic tolerance to PEEP.	
• Set ventilator alarms as follows: upper VT alarm set to 1.5 × set VT to identify double-triggering, upper RR alarm set to 37 min^−1^.	
• Procedure when PaO_2_ < 60 mmHg or SpO_2_ < 88% despite adjustments of FiO_2_ and PEEP (in the following order as needed): 1, use PP; 2, add NMBA; 3, add iNO; and 4, consider ECMO.	
• Procedure when PaO_2_ > 80 mmHg or SpO_2_ > 95% (in the following order as needed): 1, stop iNO; 2, stop NMBA if administration > 48 h; and 3, adjust FiO_2_ and PEEP.	
• Procedure when plateau pressure is > 30 cmH_2_O (in the following order as needed): 1, inject a bolus of NMBA; 2, reduce VT to 4 mL kg^−1^ PBW (if pH ≥ 7.20); and 3 decrease PEEP down to a minimum of 5 cmH_2_O.	
• Procedure when pH < 7.20 (in the following order as needed): 1, increase sedation/NMBA dose to achieve good patient-ventilator synchrony; 2, increase RR up to 35 min^−1^; 3, may administer IV bicarbonate; 4, increase VT by 1 mL kg^−1^ PBW step up to 8 mL kg^−1^ PBW if pH < 7.15; and 5, consider ECCOR or ECMO.	
• Procedure when pH > 7.45 (in the following order as needed): 1, decrease VT down to 4 mL kg^−1^ or 6 mL kg^−1^ PBW in the intervention and control groups, respectively, and 2, decrease RR	
• Procedure when VT > 4 mL kg^−1^ PBW and pH > 7.20 in the intervention group: attempt to decrease VT down to 4 mL kg^−1^ PBW at least twice daily.	
• Procedure when VT > 6 mL kg^−1^ PBW and pH > 7.20 in the control group: attempt to decrease VT down to 6 mL kg^−1^ PBW at least twice daily.	
• Procedure when RR > 35 min^−1^ or patient-ventilator asynchrony in patients treated with NMBA: inject a bolus of NMBA and increase maintenance dose.	
• Procedure when RR > 35 min^−1^ or occurrence of patient-ventilator asynchrony in patients without NMBA and either PaO_2_/FiO_2_ < 150 mmHg or PEEP > 8 cmH_2_O (in the following order as needed): 1, inject a bolus of sedation drugs and opioid and increase maintenance dose and 2, inject a bolus of NMBA and resume continuous NMBA administration.	
• Procedure when RR > 35 min^−1^ or patient-ventilator asynchrony in patients without NMBA and PaO_2_/FiO_2_ ≥ 150 mmHg and PEEP ≤ 8 cmH_2_O (in the following order as needed): 1, adjust I/E ratio; 2, inject a bolus of sedation drugs and opioid; 3, switch ventilatory mode to pressure support targeting VT between 6 and 8 mL kg^−1^; 4, inject a bolus of sedation drugs and opioid and resume deep sedation targeting RASS between − 3 and − 4 and decrease VT down to 4 mL kg^−1^ in the intervention group; and 5, bolus NMBA and resume continuous NMBA administration.	

*ECCOR* extracorporeal CO_2_ removal, *ECMO* extracorporeal membrane oxygenation, *FiO*_*2*_ fraction of inspired oxygen, *iNO* inhaled nitric oxide, *NMBA* neuromuscular blocking agent, *PaO*_*2*_ partial pressure of arterial oxygen, *PBW* predicted body weight, *PEEP* positive end-expiratory pressure, *PP* prone positioning, *RR* respiratory rate, *SpO*_*2*_ oxyhemoglobin saturation measured by pulse oximetry, *VE* minute-ventilation, *VT* tidal volume

Within the first hour after randomization, the intervention group will be managed with ultra-low tidal volume ventilation (i.e., aiming for VT 4 mL kg^−1^ PBW, PEEP set using the same PEEP-FiO_2_ table [10], aiming for a plateau pressure < 30 cmH_2_O) under deep sedation (i.e., RASS score between − 3 and − 4 [13]). RR will be set targeting a pH range between 7.20 and 7.45, and VT may be increased if pH remains below 7.15 despite a RR set to 35 min^−1^ and minimization of instrumental dead space (Table 1). This ventilatory protocol was successfully tested in a pilot randomized multicenter trial on 34 patients [8].

Adjustment of ventilatory settings in both groups will be predefined (Table 1). Both ventilatory strategies will be applied until the success of a deep sedation weaning trial (i.e., PEEP decrease down to 5 cmH_2_O with PaO_2_/FiO_2_ ≥ 150 mmHg in the supine position and VT 6 mL kg^−1^ PBW) performed daily from day 3 of the study. In case of ventilator asynchrony or RR > 35.min^−1^ despite ventilatory settings adjustments (Table 1), deep sedation will be resumed and ventilatory strategies will be reapplied according to the allocation group.

#### Criteria for discontinuing or modifying allocated interventions {11b}

Two situations are expected to require transient modification of allocated interventions, namely severe mixed acidosis and plateau pressure greater than 30 cmH_2_O.

Rules for handling severe mixed acidosis and excessive plateau pressure in the intervention group and the control group will be predefined (Table 1) and provided to the investigators in the full protocol document (Additional files 1 and 2) and in a summarized version (Additional file 5) allowing the availability of this information at the bedside.

After the resumption of severe mixed acidosis and excessive plateau pressure episodes, VT will be set according to the allocation group, and patients will be analyzed in their allocation group according to the randomization.

In case of harm related to the intervention, patients could be managed outside of the protocol at the attending physician’s discretion but will remain in their allocation group for data analysis. Patients could be withdrawn from the study at their request or at the request of their legal representative, and their data will not be analyzed.

#### Strategies to improve adherence to interventions {11c}

Adherence to the protocol procedures will be recorded daily in the electronic case report forms during the first 14 days of the study. A document summarizing the study protocol (Additional file 5) and each procedure to adjust mechanical ventilation and ARDS adjunctive therapy will be provided to the investigators, allowing availability at the bedside of the study procedures.

#### Relevant concomitant care permitted or prohibited during the trial {11d}

Patients in both groups will receive deep sedation (i.e., targeting RASS score between − 3 and − 4) after randomization. After a successful deep sedation weaning trial, patients in both groups will be ventilated with VT 6–8 mL kg^−1^ PBW, sedation dose adjusted to target a RASS score between − 2 and 0, and tested daily with a spontaneous breathing test until successful extubation. All ventilatory co-interventions will be standardized to ensure group comparability:

- Neuromuscular blocking agent use will be advocated during the first 48 h of ARDS, in case of persisting severe hypoxemia (PaO_2_/FiO_2_ < 100 mmHg with a PEEP > 10 cmH_2_O) or in case of ventilator asynchrony despite deep sedation in assist volume-controlled mode (Table 1).
- Prone position will be advocated as long as PaO_2_/FiO_2_ < 150 mmHg or PEEP > 10 cmH_2_O or FiO_2_ > 60% in the supine position.
- Extracorporeal membrane oxygenation (ECMO) will be considered if PaO_2_/FiO_2_ < 60 mmHg in the prone position.
- ECCOR will be considered in patients with persisting severe respiratory acidosis despite adjustments of the ventilatory settings and pharmacological interventions as defined in Table 1.
- Recruitment maneuvers will not be recommended.

#### Provisions for post-trial care {30}

None

### Outcomes {12}

#### Primary outcome

The primary outcome is a composite score based on 90-day all-cause mortality as the first criterion and the number of VFD at day 60 after inclusion as the second criterion. The composite score will be obtained by comparing each patient of one group to all patients of the other group. For each pair, a value of + 1 (favorable), − 1 (unfavorable), or 0 (neutral) will be given to each patient. The score will be built as follows:

- A value of + 1 will be given for a patient alive at day 90 paired with a patient deceased at day 90.
- A value of + 1 will be given for a patient alive at day 90 paired with a patient alive at day 90 but with a lower number of VFD.
- A value of 0 will be given for pairs with both patients deceased at day 90.
- A value of 0 will be given in case of an identical number of VFD for the pairs of patients alive at day 90.
- A value of − 1 will be given for a deceased patient in comparison with patients alive at day 90.
- A value of − 1 will be given for a patient alive at day 90 paired with a patient alive at day 90 but with a higher number of VFD.

For a given patient, the score will correspond to the sum of values resulting in the comparison to all patients of the other group.

VFD will be computed as follows from the day of inclusion:

- VFD = 0 if the patient dies between inclusion and day 60
- VFD = 60 − *x* if the patient is successfully weaned from invasive mechanical ventilation, with *x* being the number of days from inclusion to last successful extubation. Successful weaning from mechanical ventilation will be defined as extubation without reintubation within at least 48 h (or weaning from mechanical ventilation for at least 48 h for patients with tracheostomy)
- VFD = 0 if the patient is mechanically ventilated for more than 60 days after inclusion

#### Secondary outcomes

1. Ninety-day all-cause mortality.
2. VFD at day 60 after inclusion.
3. Time from inclusion to successful extubation.
  Successful extubation will be defined as extubation without reintubation or death within 48 h in intubated patients and as weaning of invasive mechanical ventilation for at least 48 h in tracheotomized patients.
4. Length of hospital stay from inclusion.
5. Value of respiratory parameters (PaO_2_/FiO_2_ ratio, pH, PaCO_2_, PEEP, VT, total PEEP, plateau pressure, driving pressure, respiratory rate, MP) assessed daily from inclusion to weaning of deep sedation or 14 days after inclusion whichever comes first.
  Total PEEP and plateau pressure will be measured at the end of 3-s end-expiratory and end-inspiratory pauses, respectively. Driving pressure will be defined as the difference between plateau pressure and total PEEP. MP will be computed as previously described [14], and partitioned into 3 components:

- $$ VT-\mathrm{related}\ \mathrm{MP}=0.0098\times RR\times {VT}^2\times \frac{1}{2}{El}_{rs} $$
- $$ \mathrm{PEEP}-\mathrm{related}\ \mathrm{MP}=0.0098\  RR\times VT\times {\mathrm{PEEP}}_{\mathrm{tot}} $$
- $$ \mathrm{Resistive}\ \mathrm{MP}=0.0098\times {RR}^2\times {VT}^2\times \frac{\left(1+I:E\right)}{60\times I:E}{R}_{\mathrm{aw}} $$

with RR = respiratory rate, *El*_*rs*_ = elastance of the respiratory system = driving pressure/VT, *I:E* = inspiratory-to-expiratory ratio, *R*_aw_ = airway resistance = (peak airway pressure − plateau pressure)/inspiratory flow.

6. Value of daily sedation dose (midazolam, propofol, and opioid) during the first 14 days after inclusion.
  Opioid doses will be expressed as morphine equivalent (1 mg fentanyl = 100 mg morphine, 1 mg sufentanil = 1000 mg morphine).
7. The use of rescue therapy (i.e., muscle relaxant, prone position, inhaled nitric oxide, recruiting maneuvers, ECMO) during the first 14 days after inclusion.
8. Severe mixed acidosis, ventilator-associated pneumonia, acute cor pulmonale, barotrauma, and any serious adverse events during the first 28 days after inclusion.
  Severe mixed acidosis will be defined as the association of pH < 7.15 and PaCO_2_ > 45 mmHg.
  Ventilator-associated pneumonia will be reported as clinically diagnosed by the attending physician.
  Acute cor pulmonale will be defined as the association of right ventricle dilatation (right ventricle surface/left ventricle surface > 0.6) and septal dyskinesia assessed by echocardiography.
  Barotrauma will be defined as the occurrence of any pneumothorax, pneumomediastinum, subcutaneous emphysema, or pneumatocele of more than 2 cm.
  Serious adverse event will be defined as any adverse event resulting in death, leading to a life-threatening condition, resulting in hospitalization or prolongation of hospitalization, resulting in disability or permanent damage, and resulting any other important medical event.
9. Montreal Cognitive Assessment (T-MoCA) test [15] assessed by a phone call at day 365 after inclusion.
10. SF-36 score [16] assessed by a phone call at day 365 after inclusion.
11. IES-R score [17] assessed by a phone call at day 365 after inclusion.
12. Incremental cost-effectiveness ratios of the innovative strategy compared to the reference strategy at day 90 after inclusion.

### Participant timeline {13}

**Fig. 1:**
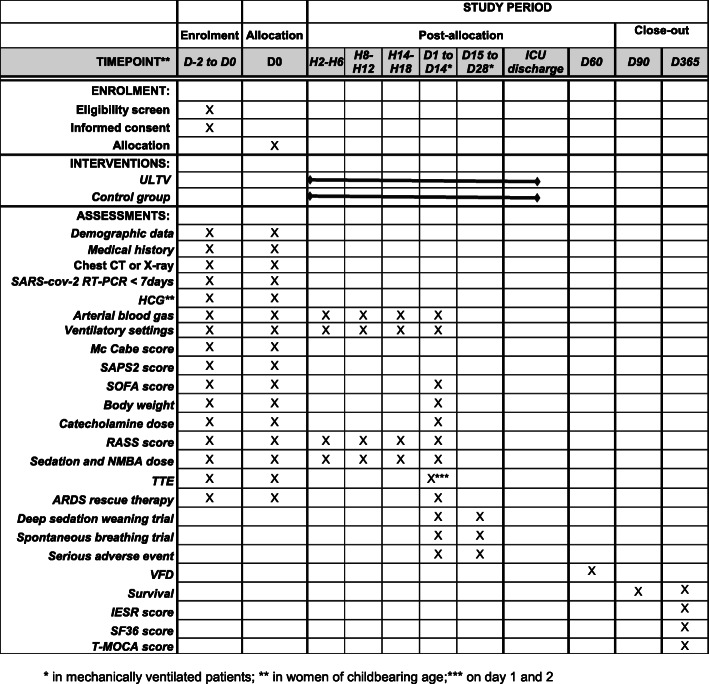
Participants’ timeline. *In mechanically ventilated patients. **In women of childbearing age. ***On days 1 and 2

Participant’s timeline is reported in Fig. 1.

### Sample size {14}

The sample size was computed through data simulations based on the following hypotheses:

- Ninety-day mortality 45% in the control group, and 40% in the intervention group (i.e., relative reduction 11%)
- Mean ± standard deviation (*SD*) VFD at day 60 28 ± 16.5 in the control group and 34.5 ± 13 in the intervention group

The expected mortality in patients with COVID-19 and moderate to severe ARDS is between 34 and 50% [1]. The mean and *SD* of VFD at day 60 in the control group was taken from the subgroup of patients with PaO_2_/FiO_2_ < 150 mmHg included in the control group of the prospective randomized Xtravent-study comparing ultra-protective ventilation with extracorporeal CO_2_ removal versus conventional protective ventilation [7]. To be conservative, we have chosen to take an expected difference between the two groups of the present study corresponding to 50% of the difference observed in the Xtravent-study between the control and the intervention groups.

For each scenario corresponding to a given sample size, 1000 datasets were simulated. For each simulation, the score was computed for each patient and compared between the two groups using the test of Mann and Whitney. The power was computed as the proportion of tests with a bilateral *p*-value < 0.05.

The results of the simulations allowed us to determine that the inclusion of 100 patients per group will achieve a power greater than 80% to conclude a significant difference between the groups.

The simulations were implemented by two biostatisticians with the SAS software, version 9.4 (Copyright (c) 2002–2003 by SAS Institute Inc., Cary, NC, USA) and the R software, version 3.6.1 (R Core Team (2012)). The results were similar.

To account for premature exits from the study (withdrawal of informed consent for example), we have planned to add 10% more patients (10 supplementary patients in each group for a total of 220 patients).

### Recruitment {15}

SARS-COV-2 epidemic is currently active in France, with a massive afflux of patients in intensive care units. Most participating centers are almost exclusively dedicated to COVID-19 patients during the peak of epidemic waves, with extended capacities, which will ensure a high inclusion rate. At the time of study design, the anticipated duration of this epidemic was several weeks for the first wave, with expected consecutive waves. The study protocol of the intervention group was successfully tested in a pilot multicenter study in 11 academic and non-academic centers, emphasizing the feasibility of the study in the multicenter design [8].

To ensure an adequate number of participants will be enrolled in the required time frame, the participating centers will be asked to report on a regular basis their problems related to enrollment in order to find adequate responses to increase the enrollment rate. This led to the modification of the trial eligibility criteria regarding co-inclusions in trials testing unlicensed new drugs or medical devices (see above). Additional centers will be sought in case the enrollment rate in the trial is too low. Finally, a 1-year extension of the study time frame will be proposed in case the enrollment rate in the trial is too low.

## Assignment of interventions: allocation

### Sequence generation {16a}

The procedure proc plan of the SAS software will be used to generate a randomization list stratified by center, with a 1:1 ratio, using random blocks of sizes 4 and 6.

### Concealment mechanism {16b}

Allocation concealment will be ensured via a central web-based system (Ennov Clinical® 7.5.720). The treatment to which a patient will be allocated will be disclosed only after enrollment in the study.

### Implementation {16c}

The randomization key will only be known to the biostatistician and the data managers. Investigators at each study site will be responsible for patient enrollment in the study. Assignment of participants to each study group will be ensured by the central web-based system (Ennov Clinical® 7.5.720®) operated by local investigators, after verification of patient eligibility and inclusion in the study.

## Assignment of interventions: blinding

### Who will be blinded {17a}

Blinding of care providers will be unpracticable as knowledge of the ventilatory settings and ventilatory measurements are required to adapt care and to monitor the safety of mechanical ventilation. Outcome assessors will not be blinded as group allocation could be deducted from ventilatory settings provided in the digital medical charts. Data analysts will be blinded from group allocation, although this may be deducted from the VT measurements provided as a variable in the dataset.

### Procedure for unblinding if needed {17b}

Not applicable

## Data collection and management

### Plans for assessment and collection of outcomes {18a}

Investigators are responsible for the assessment and collection of outcomes, baseline, and other trial data. Data will be entered in the electronic case report form by delegated team members and will be monitored by trained clinical research associates. The digital version of the case report form is provided in Additional file 6. Subjects will be assessed daily while hospitalized in the intensive care unit (ICU). Day 60 assessment will be performed by investigators or delegated team members using electronic medical records and phone calls to the patient’s general practitioner and to any MD involved in patient care after ICU discharge if required. Day 365 assessment will be performed by a phone call by investigators or delegated team members, to administer 3 tests whose validity have been proven when administered remotely [15, 18, 19]. French language versions of T-MOCA [15], IES-R [17], and SF-36 [16] questionnaires will be implemented in the electronic case report form and are provided in Additional files 7, 8, and 9.

### Plans to promote participant retention and complete follow-up {18b}

Missing values for secondary outcomes assessed during ICU stay are not expected, since patients will remain hospitalized. Missing values of VFD at day 60 are not expected as this parameter will be assessed at ICU discharge. Missing values for survival day 90 or day 365 could occur for patients surviving at hospital discharge, and the subsequent procedure will be applied to minimize the number of patients with incomplete follow-up. Upon enrollment, patients’ and their next of kin’s contact information will be stored in the digital health record at each study site. Patients will be contacted by a phone call at day 90 and day 365 to assess the vital status, to perform the T-MoCA test [15], and to compute the SF-36 score and the IES-R score. In case contact with the patient is lost, study technicians will contact the patient’s next of kin or the patient’s general practitioner in order to re-establish contact or assess the vital status. Any attempt to contact the patient will be documented in the patient’s medical records.

### Data management {19}

An electronic case report form (eCRF) will be drawn up for each included patient using Ennov Clinical® 7.5.720 central web-based system. Subjects will be identified by the first letter of their first name, the first letter of their family name, the center number, and the inclusion number. This code will be the only information featured on the eCRF enabling a retrospective link to the patient. The data collected during the study will be processed electronically in accordance with the requirements of the French Data Protection Authority (CNIL) in compliance with French Reference Methodology MR001. eCRF will be transmitted electronically and centralized in the data management department of the coordinating site.

### Confidentiality {27}

Subject confidentiality is strictly held by the participating investigators, their staff, the sponsor, and their agents. This confidentiality is extended to cover clinical information relating to subjects, test results of biological samples or medical imaging, and all other information generated during participation in the study. All electronic transmission of data that leaves each study center will be identified only by a coded number that is linked to a subject through a code key maintained at the clinical site, and eventually destroyed at the end of the study. All source records including electronic data will be stored in secured systems.

No identifiable information concerning subjects in the study will be released to any unauthorized third party. Subject confidentiality will be maintained when the study results are published or presented in conferences. The study monitor, other authorized representatives of the sponsor, and representatives of regulatory agencies may inspect all documents and records required to be maintained by the investigator, including but not limited to medical records (office, clinic, or hospital) for the subjects in this study.

### Plans for collection, laboratory evaluation, and storage of biological specimens for genetic or molecular analysis in this trial/future use {33}

No biological specimens will be collected for the sake of the trial. All biological assays reported in the case report forms are those performed in the usual care of COVID-19 patients with ARDS. No biological samples will be stored for future analysis.

## Statistical methods

### Statistical methods for primary and secondary outcomes {20a}

#### Descriptive analysis

Patients’ characteristics will be described and compared between the two groups to verify the allocation efficacy. Quantitative characteristics will be described using the following statistics: number of missing data, mean, standard deviation, quartiles, and minimum and maximum values. Qualitative characteristics will be described using the following statistics: number of missing data and absolute and relative frequency in each category. Quantitative characteristics will be compared between the two groups using the *t* test of Student or the test of Mann and Whitney. Qualitative characteristics will be compared using the *χ*^2^ test or the exact test of Fisher.

#### Analysis of the primary outcome

The main analysis will be carried out by intention to treat, i.e., all the patients included in the study will be analyzed in their initial randomization group regardless of whether the allocated ventilation strategy was effectively applied or not.

The distribution of the composite score will be described in each group using the following statistics: mean, standard deviation, quartiles, and minimum and maximum values. It will be compared between the two groups using the test of Mann and Whitney.

The ULTV effect size will be quantified by the probability of a favorable result for a patient belonging to the ULTV arm in comparison with a patient belonging to the protective ventilation arm. This probability will be estimated by the proportion of pairs for which the patient of the ULTV arm will have a favorable result [20]. This estimate will be given with a 95% confidence interval.

#### Analysis of the secondary outcomes

The analysis of the secondary objectives will also be carried out by intention to treat. Qualitative secondary outcomes such as the mortality at day 90 will be described in each group by the event proportion and compared using the *χ*^2^ test or the exact test of Fisher. Quantitative secondary outcomes such the VFD at day 60 will be described in each group by the mean and the standard deviation or the quartiles, and the minimum and maximum values according to the shape of the distribution. They will be compared between the two groups using the *t* test of Student or the test of Mann and Whitney.

All the analyses will be carried out using the SAS software, version 9.4 (Copyright (c) 2002–2003 by SAS Institute Inc., Cary, NC, USA). The comparisons will be considered statistically significant for a bilateral *p*-value < 0.5.

### Interim analyses {21b}

No interim analysis is planned.

### Methods for additional analyses (e.g., subgroup analyses) {20b}

Exploratory analysis regarding the primary outcome will be performed by comparing both arms of the study in the following subgroups of patients: age greater or lower than its median value, SAPS2 greater or lower than its median value, SOFA score at randomization greater or lower than its median value, renal SOFA at randomization < 2 and ≥ 2, bicarbonates at randomization < 22 and ≥ 22 mmol L^−1^, driving pressure at randomization greater or lower than its median value, plateau pressure at randomization greater or lower than its median value, compliance of the respiratory system at randomization greater or lower than its median value, mechanical power at randomization greater or lower than its median value, PaO_2_ at randomization ≤ 100 mmHg and > 100 mmHg, and pH at randomization greater or lower than its median value.

### Methods in analysis to handle protocol non-adherence and any statistical methods to handle missing data {20c}

A per-protocol secondary analysis will be carried out. For this analysis, the patients with major deviations to the protocol will be excluded. In particular, the population of the ULTV arm will be restricted to patients:

- With VT ≤ 4.2 mL kg^−1^ (i.e., 4 + 5% to account for measurement error) at least 50% of the days between inclusion and weaning of deep sedation or day 14 whichever comes first, in the intervention group
- With 5.4 ≤ VT ≤ 6.6 mL kg^−1^ at least 50% of the days between inclusion and weaning of deep sedation or day 14 whichever comes first, in the control group

Weaning of deep sedation will be defined as the time at which RASS score is greater than − 3 for at least 48 h or sedation drugs are weaned for at least 48 h.

The frequency of missing data will be described in each group and for each described variable. No method of imputation will be used to replace missing data.

### Plans to give access to the full protocol, participant level-data, and statistical code {31c}

After publication of the results of the trial, the data will be made partially accessible to other investigators. Data access request will be reviewed by the Trial Steering Committee that will grant access or not. To gain access, requestors will be required to sign a data access agreement. Statistical code developed for the analysis of the trial can be made accessible by request to the biostatisticians of the study.

## Oversight and monitoring

### Composition of the coordinating center and trial steering committee {5d}

This study will be conducted in a manner consistent with Good Clinical Practice. The steering committee will be composed of 5 co-authors (MR, HY, JCR, LB, MM). Drs. HY, JCR, LB, and MM will oversee all research activities. The study coordinator (HY) will be responsible, in part, for the communication with all investigators. The team leader (JCR), project manager (Loredana Baboi), and study coordinator (HY) will hold weekly meetings, and full team meetings will be held monthly. There will be no endpoint adjudication committee, as the composite primary endpoint of the study was neither complex nor subjective (VFD and survival).

### Adverse event reporting and harms {22}

Based on the pilot trial on 34 patients [8], the following adverse events are suspected to increase or decrease in association with the intervention: severe mixed acidosis, ventilator-associated pneumonia, acute cor pulmonale, and barotrauma. These expected adverse events will be collected systematically and daily in the eCRF from day 1 to day 28 of the study as long as the participants remain mechanically ventilated. Given their high frequency in relation with the severity of the underlying disease, the following non-serious adverse events will not be reported: acute kidney injury with KDIGO grade < 3, metabolic disorders, non-fatal nosocomial infections (with the exception of bacteremia and ventilator-associated pneumonia), shock (with the exception of those related to acute cor pulmonale), non-fatal worsening of respiratory condition, and ICU-acquired weakness. All other adverse events occurring from inclusion to hospital discharge will be reported through a spontaneous report.

Serious adverse event (SAE) information will be collected for the duration of the participant’s involvement in the trial. SAEs will be managed according to the best current standard of care and reported to the sponsor according to good clinical practices. All SAEs will be reported to the sponsor within one business day, in a structured narrative explaining the events that occurred. An internal safety monitor will adjudicate all SAEs for report completeness, seriousness of event, and relationship to study interventions. After receiving an unexpected SAE report, the sponsor will notify to the French regulatory agencies and the research ethics committee (CPP Ile de France 7).

Expected adverse events, SAEs, and non-serious adverse events will be reported in trial publication.

### Frequency and plans for auditing trial conduct {23}

This study will be conducted in a manner consistent with Good Clinical Practice and within the French regulatory framework. Trial conduct will be regularly audited by the sponsor (Hospices Civils de Lyon) independently from the investigators, checking for the following items:

- Verification of the validity of informed consent by every trial participant
- Identification of any violation in the trial procedures
- Quality control of data reporting in the digital case report form
- Quality control of serious adverse events reporting

An inspection or audit may also be performed by regulatory authorities. Inspectors will check the documents, logistics, records, and any other resources the authorities consider to be associated with the clinical trial and that may be located at the trial site itself.

### Composition of the data monitoring committee, its role, and reporting structure {21a}

The data monitoring committee (DMC) is composed of 3 experts independent from the coordinating investigators and its institution, with expertise in conducting clinical trials, experience in serving on other DMC, and absence of financial or intellectual conflicts of interests with the sponsor and investigators of the present trial. The 3 experts are Pr. Christine Binquet (biostatistician, CHU Dijon), Pr. Jean Dellamonica (intensivist, CHU Nice), and Pr. Boris Jung (intensivist, CHU Montpellier). The DMC operated within the framework of the charter provided in Additional file 10. After the first meeting at the beginning of the trial, the DMC will reunite after the occurrence of the first 30 deaths among the study participants, every 30 deaths thereafter until study completion, and at least every 6 months during the study. Additional DMC meetings will be convened as needed.

### Plans for communicating important protocol amendments to relevant parties (e.g., trial participants, ethical committees) {25}

Important protocol modifications will have to be approved by the research ethics committee (CPP Ile de France 7) and French regulation agencies. After approval, protocol modifications will be signaled to the trial registry, investigators, and participants in the trial.

### Dissemination plans {31a}

This trial is registered with the publicly accessible www.clinicaltrials.gov registry for dissemination and data sharing purposes (NCT04349618). Its results will be published in a high-impact, peer-reviewed journal no more than 12 months after the end of the last participant visit at day 90 and made freely available for public access within 6 months of publication. A second publication reporting long-term follow-up at day 365 after inclusion is planned. The investigators will follow the rules and guidelines of the International Committee of Medical Journal Editors [21] for authorship. In practice, the steering committee will be among the authors of the publication, as will the investigators who included the patients in the trial. We expect to prepare additional publications addressing the different facets of this work. We will also present our results at national or international scientific conferences.

## Discussion

We had two practical concerns at the time of the study design. The first concern was related to a possible low inclusion rate. Indeed, the research ethic committee initially denied the possibility of co-inclusion in the present trial and in any clinical trial involving unlicensed new drugs or medical devices. As most intensive care units in France were participating in multiple drug trials testing antiviral or immunomodulatory drugs against COVID-19 pneumonia, we anticipated that the study would require at least 1 year for completion.

The second concern was related to the limited study funding, limiting the number of participating centers and restraining the geographical area of participating centers to limit monitoring costs.

## Trial status

This trial is ongoing. Enrollment began on April 15, 2020, and is expected to be completed on April 15, 2021.

## Supplementary Information

**Additional file 1.** Study protocol version 3.

**Additional file 2.** Study protocol version 4.

**Additional file 3.** WHO Trial Registration Dataset.

**Additional file 4.** Study sites.

**Additional file 5.** Protocol summarized version.

**Additional file 6.** Case report form.

**Additional file 7.** TMoCA questionnaire (French version).

**Additional file 8.** IES-R questionnaire (French version).

**Additional file 9.** SF36 questionnaire (French version).

**Additional file 10.** DSMB charter.

**Additional file 11.** Funding document.

**Additional file 12.** Ethical approval document (French).

**Additional file 13.** Ethical approval document (English).

**Additional file 14.** Consent form (patients).

**Additional file 15.** Consent form (surrogates).

**Additional file 16.** Confirmation form (patient).

## Abbreviations

ARDS: Acute respiratory distress syndrome
BAL: Broncho-alveolar lavage
DMC: Data monitoring committee
ECCOR: Extracorporeal CO_2_ removal
ECMO: Extracorporeal membrane oxygenation
FiO_2_: Fraction of inspired oxygen
ICU: Intensive care unit
IES-R: Impact of event scale - revised
MP: Mechanical power
PaCO_2_: Carbon dioxide partial pressure in the arterial blood
PaO_2_: Oxygen partial pressure in arterial blood over fraction of inspired oxygen
PBW: Predicted body weight
PEEP: Positive end-expiratory pressure
RASS: Richmond agitation sedation scale
T-MoCA: Telephonic Montreal cognitive assessment test
ULTV: Ultra-low tidal volume ventilation
VFD: Ventilator-free days
VT: Tidal volume
