# Supplementary material for: Open-label randomized controlled trial of ultra-low tidal ventilation without extracorporeal circulation in patients with COVID-19 pneumonia and moderate to severe ARDS: study protocol for the VT4COVID trial
Source: Trials. 2021 Oct 11;22:692. doi: 10.1186/s13063-021-05665-z (PMC8503716; doi:10.1186/s13063-021-05665-z)
Supplement: Supplementary file 1 — Additional file 1. Study protocol version 3. [file 13063_2021_5665_MOESM1_ESM.pdf]

Ventilation avec ultra faible volume courant chez les patients avec pneumonie à COVID-19 et SDRA modérément sévère à sévère – Etude randomisée contrôlée en ouvert

VT4-COVID

Protocole impliquant la personne humaine de catégorie 1 - HPS

Version 3 du 12/04/2020

Promoteur : Hospices Civils de Lyon
BP 2251
3 quai des Célestins,
69229 LYON cedex 02

Investigateur coordonnateur : YONIS Hodane, PH
Service de Médecine Intensive Réanimation
Groupement Hospitalier Nord
Hôpital de la Croix Rousse
103 Grande rue de la Croix Rousse
69004 Lyon
Téléphone : 04 72 07 17 62. Fax : 04 72 07 17 74.
Hodane.yonis@chu-lyon.fr

Code promoteur : 69HCL20_0322
N° IDRCB : 2020-A00869-30
Numéro d'enregistrement clinicaltrials.gov : *NCTXXXXX*
Avis favorable du CPP Ile de France VII le : 14/04/2020
Autorisation de l'ANSM le : 06/04/2020

RESUME

**TITRE**	Ventilation avec ultra faible volume courant chez les patients avec pneumonie à COVID-19 et SDRA modérément sévère à sévère – Etude randomisée contrôlée en ouvert - VT4-COVID
**PROMOTEUR**	Hospices Civils de Lyon<br>BP 2251<br>3 quai des Célestins,<br>69229 LYON cedex 02
**INVESTIGATEUR COORDONNATEUR**	Dr Hodane YONIS<br>Service de médecine Intensive Réanimation<br>Hôpital de la Croix-Rousse – Groupement Hospitalier Nord<br>93 grande rue de la Croix-Rousse 69004 - Lyon<br>Tél : 04 26 10 92 71 ; Fax : 04 72 07 17 74<br>Email : hodane.yonis@chu-lyon.fr
**VERSION DU PROTOCOLE**	3 du 12/04/2020
**JUSTIFICATION / CONTEXTE**	Le syndrome de détresse respiratoire aiguë (SDRA) est la complication la plus sévère de la pneumonie à Covid-19. La mortalité du SDRA compliquant une pneumonie à Covid-19 est extrêmement élevée et atteint 50 à 60%. Le seul traitement efficace à ce jour de cette pathologie est la ventilation mécanique, dans l'attente de la guérison. Une des caractéristiques de ce SDRA est la durée de ventilation mécanique particulièrement longue, ce qui fait que ces patients sont particulièrement exposés aux effets délétères potentiels de la ventilation mécanique. On sait qu'un volume courant (VT) excessif réglé sur le respirateur est associé à une surmortalité, ce qui fait que tous les patients avec SDRA sont actuellement ventilés avec un VT de 6 ml/kg, soit une ventilation supposée protectrice sur le poumon. Toutefois, il existe de nombreux arguments suggérant que le VT est excessif chez les patients les plus graves, dont la mortalité pourrait être abaissée lorsque qu'une ventilation ultraprotectrice (c-à-d avec un VT de 4 ml/Kg) est appliquée combinée avec une épuration extracorporelle de CO2 pour compenser l'augmentation de CO2 liée à la baisse du VT. Les limites de la ventilation ultraprotectrice associée à l'épuration extracorporelle de CO2 sont les suivantes : faible niveau de preuve, faible disponibilité des épurateurs extracorporels de CO2, coûts très élevés des circuits devant être changés toutes les 72 heures, nécessité d'anticoagulation importante compte-tenu des débits sang relativement faibles, iatrogénie liée à l'implantation des canules et au traitement anticoagulant, inapplicabilité de ces stratégies dans les pays émergents…Dans le contexte spécifique des patients Covid-19 avec SDRA, nous avons pu observer sur 2 des 4 patients étudiés avec un scanner quantitatif inclus dans un protocole de recherche observationnel la présence d'hyperinflation intracycle en quantité substantielle suggérant que ces patients sont particulièrement à risque d'agression pulmonaire induite par la ventilation mécanique liée à un volume courant encore trop élevé.<br>Nous avons récemment démontré la faisabilité et la sécurité d'une ventilation ultraprotectrice sans épuration extracorporelle de CO2 sur 34 patients avec SDRA. Cette stratégie a pu être appliquée pendant la totalité de l'évolution du SDRA, sans nécessité d'augmenter le traitement sédatif, et en observant un taux de patients avec cœur pulmonaire aigu plus faible qu'attendu (6% vs. plus de 20% attendu[1])<br>En ce qui concerne la sécurité, la mortalité à J90 de ces patients était de 41%, en dépit de l'inclusion de patients moribonds, mais 32% des patients de la série ont présenté des épisodes d'acidose mixte sévère transitoires,

	contrôlés par des adaptations secondaires des réglages ventilatoires. Ce risque serait probablement minimisé en ne proposant pas cette stratégie aux patients avec acidose sévère (pH < 7,21)<br>Notre hypothèse est que la ventilation ultraprotectrice sans épuration extracorporelle peut réduire la mortalité et la durée de ventilation mécanique du SDRA compliquant une pneumopathie à Covid-19, en réduisant les effets délétères de la ventilation mécanique, comme la ventilation mécanique prolongée est à ce jour le seul traitement efficace pour maintenir ces patients en vie.
**OBJECTIFS**	• **Objectif principal :** Evaluer le bénéfice de la ventilation ultraprotectrice en comparaison avec la ventilation protectrice sur un critère de jugement composite incluant la mortalité à J90 comme critère prioritaire et le nombre de jours vivants sans ventilation mécanique à J60 comme second critère<br>• **Objectif(s) secondaire(s) :**<br>*Objectifs d'efficacité :*<br>1. Tester si la ventilation ultraprotectrice est associée à une baisse de la mortalité toute cause à J90 en comparaison avec le groupe contrôle (analyse en intention de traiter)<br>2. Tester si la ventilation ultraprotectrice est associée à une augmentation du nombre de jours vivant sans ventilation mécanique à J60 en comparaison avec le groupe contrôle<br>3. Tester si la ventilation ultraprotectrice est associée à une baisse de la mortalité toute cause à J90 en comparaison avec le groupe contrôle (analyse per protocole)<br>4. Tester si la ventilation ultraprotectrice est associée à une diminution du temps entre inclusion et extubation avec succès en comparaison avec le groupe contrôle<br>5. Tester si la ventilation ultraprotectrice est associée à une diminution de la durée de séjour hospitalière depuis l'inclusion en comparaison avec le groupe contrôle<br>6. Tester si la ventilation ultraprotectrice impacte les paramètres ventilatoires suivants mesurés quotidiennement pendant les 7 premiers jours suivant l'inclusion, en comparaison avec le groupe contrôle (rapport entre la pression partielle en oxygène dans le sang artériel et la fraction inspirée en oxygène (PaO_2/FiO_2), pH, pression partielle en CO_2 dans le sang artériel ($PaCO_2$), pression expiratoire positive (PEP), volume courant (VT), PEP totale, pression de crête, pression plateau, pression de travail, fréquence respiratoire, temps inspiratoire)<br>*Objectifs de sécurité*<br>7. Tester si la ventilation ultraprotectrice n'est pas associée à une augmentation des doses de sédation mesurées quotidiennement pendant les 7 premiers jours suivant l'inclusion, en comparaison avec le groupe contrôle<br>8. Tester si la ventilation ultraprotectrice est associée à une réduction du taux de recours aux thérapeutiques de sauvetage au cours des 7 premiers jours suivant l'inclusion (décubitus ventral, curares, monoxyde d'azote inhalé, manœuvres de recrutement, ECMO)<br>9. Tester si la ventilation ultraprotectrice est associée à une réduction de la densité d'incidence des effets indésirables suivants, en comparaison avec le groupe contrôle : acidose mixte sévère, pneumonie acquise sous ventilation mécanique, cœur pulmonaire aigu, barotraumatisme, ou tout effet indésirable grave)<br>*Objectifs centrés patient*

	10. Tester si la ventilation ultraprotectrice n'est pas associée à une dégradation des performances cognitives à J365 de l'inclusion, en comparaison avec le groupe contrôle<br>11. Tester si la ventilation ultraprotectrice n'est pas associée à une dégradation de la qualité de vie à J365 de l'inclusion, en comparaison avec le groupe contrôle<br>12. Tester si la ventilation ultraprotectrice n'est pas associée à une augmentation du taux de patients avec syndrome de stress post-traumatique à J365 de l'inclusion, en comparaison avec le groupe contrôle<br>*Objectifs d'efficience*<br>13. Evaluer l'efficience de la ventilation ultraprotectrice, en comparaison avec le groupe contrôle à J90 de l'inclusion
**METHODOLOGIE / SCHEMA DE LA RECHERCHE**	Etude multicentrique régionale, prospective, de supériorité, ouverte, randomisée et contrôlée, avec 2 groupes parallèles et randomisation balancée avec un ratio 1 pour 1.<br>Cette étude se qualifie comme une étude interventionnelle de catégorie 1.
**CRITERES DE JUGEMENT**	• **Critère principal :** score composite incluant la mortalité à J90 comme critère priorisé et le nombre de jours vivants sans ventilation mécanique à J60 comme second critère, obtenu en comparant chaque patient d'un groupe, à l'ensemble des patients de l'autre groupe.<br>• **Critère(s) secondaire(s) :**<br>1. Mortalité toute cause à J90 en intention de traiter dans les 2 bras<br>2. Nombre de jours vivant sans ventilation mécanique à J60 (VFD-J60) dans les 2 bras<br>3. Mortalité toute cause à J90 per protocole dans les 2 bras<br>4. Temps entre randomisation et extubation avec succès définie comme une extubation sans ré-intubation dans les 48 heures dans les 2 bras<br>5. Durée de séjour hospitalière définie comme l'intervalle entre la randomisation et la sortie de l'hôpital dans les 2 bras<br>6. Paramètres respiratoires suivants mesurés journalièrement entre l'inclusion et l'arrêt de la sédation lourde (et au maximum J14) dans les 2 bras (PaO2/FiO2, pH, PaCO2, PEP, VT, PEP totale, pression de crête, pression plateau, pression de travail, fréquence respiratoire, temps inspiratoire)<br>7. Dose de benzodiazépines, de propofol, et d'opiacés exprimée en équivalent morphine entre l'inclusion et J14.<br>8. Taux de recours aux thérapeutiques de sauvetage au cours des 7 premiers jours suivant l'inclusion dans les 2 bras (décubitus ventral, curares, monoxyde d'azote inhalé, manœuvres de recrutement, ECMO)<br>9. Densité d'incidence dans les 2 bras des effets indésirables suivants :<br>a. acidose sévère (définie par un pH<7.15 et une PaCO2>45 mm Hg),<br>b. pneumonie acquise sous ventilation mécanique<br>c. cœur pulmonaire aigu défini en échocardiographie par l'association d'un ratio surface du ventricule droit sur gauche > 0,6 et d'un trouble de la cinétique septale<br>d. barotraumatisme (pneumothorax, ou pneumomédiastin, ou emphysème sous cutané ou pneumatocèle de plus de 2 cm en imagerie)<br>e. tout effet indésirable grave<br>10. Score de trouble cognitif T-MOCA dans les 2 bras (MOntreal Cognitive Assessment) évalué par téléphone à J365 de l'inclusion<br>11. Score de qualité de vie SF-36 évalué par téléphone à J365 de l'inclusion

	12. Score de stress post-traumatique dans les 2 bras par le score IES-R à J365 de l'inclusion<br>13. Ratio coût efficacité de la stratégie de ventilation ultraprotectrice en comparaison du groupe contrôle à J90 de l'inclusion
**POPULATION CIBLE**	Cette étude porte sur des sujets adultes atteints de pneumonie à Covid-19, avec SDRA modérément sévère à sévère
**CRITERES D'INCLUSION**	1. Adulte âgé d'au moins 18 ans<br>2. Intubation et ventilation mécanique<br>3. Pneumonie à COVID-19 confirmée par RT-PCR sur prélèvement nasopharyngé ou du tractus respiratoire datant de moins de 7 jours<br>4. Insuffisance respiratoire aiguë non complètement expliquée par une insuffisance ventriculaire gauche ou une surcharge hydrosodée<br>5. Opacités radiologiques pulmonaires bilatérales non complètement expliquées par des épanchements pleuraux ou atélectasies ou des nodules<br>6. Ventilation mécanique invasive avec $PaO_2/FiO_2 \leq 150$ mm Hg et PEP ≥ 5 cm H_2O avec un VT ≤ 6 ml/kg de PPT<br>7. Sédation intraveineuse continue dans le cadre du traitement du SDRA
**CRITERES DE NON-INCLUSION**	*Critères de non-inclusion relatifs à l'histoire de la maladie*<br>1. Ventilation mécanique invasive ou non-invasive depuis plus de 48 heures (oxygénothérapie à haut débit autorisée sans limite), idéalement le plus tôt possible<br>2. Patient précédemment inclus dans le même protocole de recherche<br>*Critères de non-inclusion relatifs à la sévérité de la maladie*<br>3. pH artériel < 7.21 malgré une fréquence respiratoire à 35/min au moment de l'inclusion<br>4. Traitement par assistance extracorporelle (ECMO ou épuration de CO2)<br>5. Pneumothorax ou fistule bronchopleurale<br>*Critères de non-inclusion relatifs aux pathologies associées entraînant des risques particuliers*<br>6. Hypertension intracrânienne (suspectée ou confirmée)<br>*Critères de non-inclusion relatifs aux comorbidités*<br>7. BPCO connue définie par un score de GOLD score ≥ 3<br>8. Insuffisance respiratoire chronique avec indication d'oxygénothérapie au long cours ou assistance ventilatoire au long cours (hypoxémie chronique)<br>9. Obésité morbide définie par un poids supérieur à 1 kg/cm<br>10. Drépanocytose<br>11. Greffe de moelle récente, aplasie post-chimiothérapie<br>12. Brûlure étendue (> 30% de la surface corporelle)<br>13. Cirrhose hépatique grave (Child-Pugh C)<br>14. Décision de limitation des thérapeutiques actives<br>*Critères de non-inclusion relatifs à la réglementation*<br>15. Patient se trouvant en période d'exclusion suite à la participation à une autre recherche impliquant la personne humaine de catégorie 1 ou inclus dans une recherche impliquant la personne humaine de catégorie 1, ou inclus dans une recherche impliquant la personne

	humaine partageant le même critère de jugement principal que la présente étude<br>16. Grossesse, Femmes allaitantes<br>17. Patient majeur protégé au sens de la loi<br>18. Patient non bénéficiaire d'un régime de sécurité sociale<br>19. Consentement de participation non obtenu (soit auprès du patient lui-même, soit auprès d'un de ses proches, soit enfin auprès de la personne de confiance que le patient aurait préalablement désigné par écrit) sauf si recours à la procédure d'urgence en l'absence de proche
**CRITERES DE SORTIE D'ETUDE**	La participation du patient à l'étude s'arrête si le patient (ou sa personne de confiance) retire son consentement
**PROCEDURES**	La stratégie à l'étude est une stratégie conforme aux recommandations internationales [2] avec comme seule différence une réduction du volume courant avec comme cible 4 ml/kg de PPT (ventilation ultraprotectrice).<br>Le traitement de comparaison est une stratégie conforme aux recommandations internationales avec un volume courant à 6 ml/kg de PPT (ventilation protectrice).<br>Les objectifs thérapeutiques ventilatoires dans les 2 bras sont conformes aux modalités actuelles de prise en charge du SDRA :<br>- Pression plateau mesurée par une occlusion télé-inspiratoire de 3 secondes ≤ 30 cm H_2O<br>- PaO_2 comprise entre 60 et 80 mm Hg ou SpO_2 comprise entre 88% et 95% avec les valeurs de PaO_2 prioritaires sur les valeurs de SpO_2<br>- pH artériel compris entre 7,20 et 7,45
**RAPPORT BENEFICES/RISQUES**	*Bénéfices :*<br>Les bénéfices individuels potentiels associés à l'application de la stratégie testée sont un meilleur contrôle de l'agression pulmonaire induite par la ventilation mécanique, une réduction de la durée de la ventilation mécanique, une sortie plus précoce de réanimation et un effet bénéfique sur la mortalité des patients.<br><br>Les bénéfices collectifs sont une réduction de la saturation des lits de réanimation dans le contexte pandémique, si la durée de ventilation et la durée de séjour en réanimation sont réduites par la ventilation ultraprotectrice.<br><br>Le seul risque associé à l'application de la stratégie testée identifié dans une étude pilote sur 34 patients est un surrisque d'épisodes d'acidose respiratoire sévère transitoires observé chez environ 30% des patients dans l'étude pilote<br><br>Les contraintes liées à la participation à l'étude sont les suivantes :<br>- pas de visite, de prélèvement biologique ou d'examen radiologique supplémentaire lié à l'étude<br>- appel téléphonique à J365 de l'inclusion pour les questionnaires de qualité de vie, d'évaluation des troubles cognitifs et du syndrome de stress post-traumatique. Cet appel durera environ 45 minutes
**NOMBRE DE SUJETS**	100 sujets par bras de randomisation soit 200 au total
**DUREE DE L'ETUDE**	Durée de la période d'inclusion : 12 mois<br>Durée de la participation pour chaque sujet : 365 jours ± 10 jours<br>Durée totale de l'étude : 24 mois
**LIEU DE LA RECHERCHE**	11 services de réanimation régionaux
**RETOMBEES ATTENDUES**	L'étude pourrait permettre une avancée thérapeutique majeure dans le traitement des formes graves de pneumonie à Covid-19 avec SDRA, pour

	lequel il n'existe actuellement aucun traitement étiologique, et où le seul traitement efficace est la ventilation artificielle dans l'attente de la guérison. Dans cette optique, la réduction de l'agression pulmonaire induite par la ventilation mécanique (VILI) est un objectif majeur, qui pourrait être démontré en cas de positivité de l'étude. Cette diminution du VILI, si elle est avérée, devrait réduire la fréquence des défaillances d'organe extra-respiratoire notamment rénale, qui ont un poids très fort dans la mortalité du SDRA. Par ailleurs, on attend une réduction du barotraumatisme (identifié chez 5 à 10% des patients sous la forme de pneumothorax en dépit de la ventilation protectrice), et de la durée de séjour en réanimation (un paramètre critique dans le contexte d'une saturation des lits de réanimation en condition épidémique).<br>Cette étude va aussi permettre d'évaluer l'impact à long terme de l'hypercapnie permissive sur la qualité de vie, le syndrome de stress post-traumatique et les altérations cognitives post-réanimation qui sont extrêmement fréquents. Enfin, dans le contexte plus global de maladies infectieuses émergentes à tropisme respiratoire, cette étude pourrait avoir un impact majeur en termes de santé publique pour les raisons suivantes :<br>- Le nombre de patient ventilés avec SDRA (c'est-à-dire à risque d'agression pulmonaire induite par la ventilation mécanique) est amené à augmenter considérablement par plusieurs ordres de magnitude<br>- La stratégie testée est adaptable à des pays en voie de développement ou émergents dans la mesure où elle ne nécessite aucune technologie additionnelle (comme la circulation extracorporelle), et repose seulement sur des adaptations ventilatoires.

LISTE DES ABREVIATIONS

**AMM**	Autorisation de Mise sur le Marché
**ANSM**	Agence Nationale de Sécurité des Médicaments et des produits de santé
**ARC**	Attaché de Recherche Clinique
**BPC**	Bonnes Pratiques Cliniques
**CNIL**	Commission Nationale Informatique et Liberté
**CPP**	Comité de Protection des Personnes
**CRF**	Case Report Form (cahier d'observation)
**EI**	Evénement Indésirable
**EIG**	Evénement Indésirable Grave
**FiO2**	Fraction inspirée en oxygène
**FN**	Fait Nouveau
**HCL**	Hospices Civils de Lyon
**ICH**	International Conference on Harmonisation
**MR**	Méthodologie de Référence
**PaCO2**	Pression partielle en di-oxyde de carbone dans le sang artériel
**PaO2**	Pression partielle en oxygène dans le sang artériel
**PEP**	Pression expiratoire positive
**PPT**	Poids prédit par la taille
**PUI**	Pharmacie à Usage Intérieur
**RCP**	Résumé des Caractéristiques du Produit
**RGPD**	Règlement Général sur la Protection des Données
**SDRA**	Syndrome de détresse respiratoire aiguë
**SUSAR**	Suspected Unexpected Serious Adverse Reaction
**TEC**	Technicien d'Etude Clinique
**VRB**	Volontaires pour les Recherches Biomédicales
**VT**	Volume courant

SOMMAIRE

1 INFORMATIONS GENERALES 12
1.1. Titre 12
1.2. Identifiants du projet et historique des mises à jour 12
1.3. Promoteur 12
1.4. Investigateurs 13
1.4.1. Investigateur coordonnateur 13
1.4.2. Investigateurs associés 13
Pr Frédéric AUBRUN 13
Pr Jean-Christophe RICHARD 13
Pr Thomas RIMMELE 13
Pr Laurent ARGAUD 13
Dr Arnaud FRIGGERI 13
Dr Bertrand DELANNOY 13
Dr Michel MULLER 14
Dr Christian POMMIER 14
Dr Claire DUPUIS 14
Pr Nicolas TERZI 14
Pr Guillaume THIERY 14
1.5. Scientifiques associés 14
1.6. Méthodologiste - Biostatisticien 14
Dr Muriel RABILLOUD 14
1.7. Economiste de la santé 14
Hassan SERRIER 14
1.8. Pharmacien 15
1.9. Comités 15
1.9.1. Comité de pilotage 15
1.9.2. Comité de surveillance indépendant 15
1.9.3. Autres comités 15
2 JUSTIFICATION SCIENTIFIQUE 15
2.1 Etat actuel des connaissances - Rationnel 15
2.2 Hypothèse de la recherche 17
2.3 Justification des choix méthodologiques 17
2.4 Population cible 17
2.5 Rapport bénéfices/risques 17
2.6 Retombées attendues 19
3 OBJECTIFS DE LA RECHERCHE 19
3.1 Objectif principal 19
3.2 Objectifs secondaires 19
4 CONCEPTION DE LA RECHERCHE 20
4.1 Type d'étude 20
4.2 Méthode pour la randomisation 20
4.3 Critères de jugement 21
4.3.1 Critère de jugement principal 21
4.3.2 Critères de jugement secondaires 21
5 CRITERES D'ELIGIBILITE 22
5.1 Critères de pré-inclusion 22
5.2 Critères d'inclusion 22
5.3 Critères de non inclusion 22
5.4 Critères de sortie prématurée 23
5.5 Inclusions concomitantes 23
5.6 Modalités de recrutement et faisabilité 23

6 TRAITEMENTS EXPERIMENTAUX / STRATEGIES EXPERIMENTALES 24
6.1 Traitement à l'étude 24
6.2 Traitement de comparaison 25
6.3 Insu 25
6.4 Traitements associés autorisés et interdits 25
7 ORGANISATION GÉNÉRALE 30
7.1 Calendrier de l'étude 30
7.2 Schéma général et tableau récapitulatif 30
7.3 Déroulement de l'étude 31
7.3.1 Screening – Pré-inclusion 31
7.3.2 Visite d'inclusion / Randomisation 32
7.3.3 Visites de suivi 33
7.3.4 Visite de fin de la recherche 35
7.3.5 Echantillons biologiques 35
7.3.6 Distinction soins et recherches 36
7.4 Règles d'arrêt temporaire ou définitif 36
7.5 Collection d'échantillons biologiques 37
8 ÉVALUATION DE LA SÉCURITÉ 37
8.1 Définitions 37
8.1.1 Evénement indésirable 37
8.1.2 Evénement ou effet indésirable grave (EIG) 37
8.1.3 Effet indésirable (EI) 37
8.1.4 Effet indésirable inattendu 37
8.1.5 Fait nouveau 37
8.2 Responsabilités de l'investigateur 38
8.2.1 Modalités de détection et de recueil des événements indésirables 38
8.2.2 Notification des EIG 38
8.2.3 Evaluation de la causalité 39
8.2.4 Période de notification des EIG, restriction des EI/EIG et événements indésirables d'intérêt particulier 39
8.3 Responsabilités du promoteur 41
8.3.1 Déclaration aux autorités compétentes 41
8.3.2 Description des effets indésirables liés à la recherche (référence de sécurité pour l'évaluation du caractère attendu/inattendu par le promoteur) 41
8.4 Comité de surveillance Indépendant 41
9 Evaluation médico-économique 42
9.1 Principales caractéristiques de l'évaluation 42
9.1.1 Type d'étude 42
9.1.2 Perspective et horizon temporel 42
9.2 Evaluation des coûts 42
9.2.1 Recueil des données de coût 42
9.2.2 Identification, quantification et valorisation des coûts 43
9.3 Présentation et interprétation des résultats 43
10 ASPECTS STATISTIQUES 43
10.1 Nombre de sujets nécessaires 43
10.2 Population d'analyse 44
10.3 Méthode statistiques 44
10.4 Analyses intermédiaires 45
10.5 Méthode de prise en compte des données manquantes 45
10.6 Gestion des modifications apportées au plan d'analyse 45
10.7 Responsable des analyses 45
11 SURVEILLANCE DE LA RECHERCHE 46

12 DROITS D'ACCES AUX DONNEES ET DOCUMENTS SOURCES.............................. 46
12.1 Accès aux données..46
12.2 Documents sources..46
12.3 Confidentialité des données..46
13 CONTROLE ET ASSURANCE DE LA QUALITE 47
14 CONSIDERATIONS ETHIQUES 47
14.1 Autorités compétentes...47
14.2 Modifications substantielles...47
14.3 Information du patient et formulaire de consentement écrit...48
14.4 Déclaration de conformité...48
14.5 Période d'exclusion 48
14.6 Indemnisation des sujets et inscription au fichier national des personnes se prêtant à une recherche interventionnelle sur la personne humaine du 1°..48
15 GESTION ET CONSERVATION DES DONNEES 49
15.1 Cahier d'observation..49
15.2 Gestion des données 49
15.3 CNIL..49
15.4 Archivage 50
16 FINANCEMENT ET ASSURANCE 50
16.1 Budget de l'étude...50
16.2 Assurance...50
17 REGLES RELATIVES A LA PUBLICATION.. 50
18 REFERENCES BIBLIOGRAPHIQUES 51

1 INFORMATIONS GENERALES

1.1. Titre

Ventilation avec ultra faible volume courant chez les patients avec pneumonie à COVID-19 et SDRA modérément sévère à sévère – Etude randomisée contrôlée en ouvert - VT4-COVID

1.2. Identifiants du projet et historique des mises à jour

Code promoteur : 69HCL20_0322
N°IDRCB : 2020-A00869-30
Numéro d'enregistrement clinicaltrials.gov : *NCTXXXXX*
Avis favorable du CPP Ile de France VII le : 14/04/2020
Autorisation de l'ANSM le : 06/04/2020

Historique des versions		
**Version**	**Date**	**Motif de la mise à jour**
1	02/04/2020	DAE – Soumission initiale
2	10/04/2020	DAE _ Réponse aux remarques du CPP
**3**	**12/04/2020**	**DAE _** Réponse aux remarques du CPP n°2 – **VERSION ACCEPTEE**

1.3. Promoteur

i. Identité :

Hospices Civils de Lyon
BP 2251
3 Quai des Célestins
69229 LYON Cedex 02

ii. Signature du protocole au nom du Promoteur :

Dr Lucilla MANSUY, Directrice de la Recherche Clinique et de l'Innovation
Hospices Civils de Lyon, Direction de la Recherche Clinique et de l'Innovation, Siège Administratif, BP 2251, 3 Quai des Célestins, 69229 LYON Cedex 02
Tél : 04 72 40 68 50, Fax : 04 72 40 68 69

iii. Responsable de la recherche au niveau du Promoteur :

Valérie PLATTNER, médecin référent
Hospices Civils de Lyon, Direction de la Recherche Clinique et de l'Innovation, Siège Administratif, BP 2251, 3 Quai des Célestins, 69229 LYON Cedex 02
Tél : 04 72 40 68 40, Fax : 04 72 11 51 90

iv. Responsable de la vigilance des essais au niveau du Promoteur :

Marina NGUON, pharmacien référent
Hospices Civils de Lyon, Direction de la Recherche Clinique et de l'Innovation, Siège Administratif, BP 2251, 3 Quai des Célestins, 69229 LYON Cedex 02
Tél : 04 72 40 68 26, Fax : 04 72 11 51 90

1.4. Investigateurs

1.4.1. Investigateur coordonnateur

Dr YONIS Hodane
Service de Médecine Intensive Réanimation
Hôpital de la Croix Rousse
103 Grande rue de la Croix Rousse 69004 Lyon
Téléphone : 04 72 07 17 62. Fax : 04 72 07 17 74.
Email : hodane.yonis@chu-lyon.fr

1.4.2. Investigateurs associés

Pr Frédéric AUBRUN
Service de Réanimation Chirurgicale
Hôpital de la Croix Rousse
103 Grande rue de la Croix Rousse 69004 Lyon
Téléphone : 04 26 10 92 34 Fax : 04 26 10 28 30
Email : frederic.aubrun@chu-lyon.fr

Pr Jean-Christophe RICHARD
Service de médecine Intensive Réanimation
Hôpital de la Croix-Rousse
103 grande rue de la Croix-Rousse
Téléphone : 04 72 07 17 62 Fax : 04 72 07 17 74.
Email : j-christophe.richard@chu-lyon.fr

Pr Thomas RIMMELE
Service de Réanimation Chirurgicale
Hôpital Edouard Herriot
5 place d'Arsonval 69003 Lyon
Téléphone : 04 72 11 07 84 Fax : 04 72 11 09 59
Email : thomas.rimmele@chu-lyon.fr

Pr Laurent ARGAUD
Service de Médecine Intensive Réanimation
Hôpital Edouard Herriot
5 place d'Arsonval 69003 Lyon
Téléphone : 04 72 11 28 62 Fax : 04 72 11 01 10
Email : laurent.argaud@chu-lyon.fr

Dr Arnaud FRIGGERI
Service de Réanimation Polyvalente
Centre Hospitalier Lyon Sud
Chemin Grand Revoyet 69310 Pierre-Bénite
Téléphone : 04 78 86 56 47 Fax : 04 78 86 30 88
Email : arnaud.friggeri@chu-lyon.fr

Dr Bertrand DELANNOY
Service de Réanimation
Clinique de la Sauvegarde
480 Avenue Ben Gourion, 69009 Lyon
Téléphone : 06 81 30 37 94
Email : bertrand.delannoy@gmail.com

Dr Michel MULLER
Service de Réanimation
Centre hospitalier Annecy Genevois
1 avenue de l'hôpital Metz-Tessy – BP 90074 74374 Pringy Cedex
Téléphone : 04 50 63 60 30
Email : *mmuller@ch-annecygenevois.fr*

Dr Christian POMMIER
Service de réanimation Polyvalente
Centre Hospitalier Saint Joseph-Saint Luc
20 Quai Claude Bernard, 69007 Lyon
Téléphone : 04 78 61 82 09
Email : *cpommier@ch-stjoseph-stluc-lyon.fr*

Dr Claire DUPUIS
Service de Médecine Intensive Réanimation
CHU Gabriel Montpied
58, Bd Montalembert – 63003 Clermont-Ferrand Cedex 1
Téléphone : 04 73 75 07 50
Email : *cdupuis1@chu-clermontferrand.fr*

Pr Nicolas TERZI
Service de Médecine Intensive Réanimation
Hôpital Michallon - CHU Grenoble Alpes
Avenue Maquis du Grésivaudan, 38700 La Tronche
Téléphone : 04-76-76-87-79
Email : *nterzi@chu-grenoble.fr*

Pr Guillaume THIERY
Service de Médecine Intensive Réanimation
Hôpital Nord – CHU Saint-Etienne
Avenue Albert Raimond 42055 SAINT-PRIEST-EN-JAREZ
Téléphone : 04 77 12 78 62
Email : *guillaume.thiery@chu-st-etienne.fr*

1.5. Scientifiques associés

Non applicable

1.6. Méthodologiste - Biostatisticien

Dr Muriel RABILLOUD
Laboratoire Biostatistique-Santé - Service de Biostatistique
162 Avenue Lacassagne 69424 Lyon Cedex 03
Téléphone : 04 72 11 57 22
Email : *muriel.rabilloud@chu-lyon.fr*

1.7. Economiste de la santé

Hassan SERRIER
Hospices Civils de Lyon
Cellule Innovation – DRCI
Evaluation économique en santé – Pôle Santé Publique
162 Avenue Lacassagne 69424 Lyon cedex 3
Téléphone : 04 72 11 54 26 ; E-mail : *hassan.serrier@chu-lyon.fr*

1.8. Pharmacien

Non applicable

1.9. Comités

1.9.1. Comité de pilotage

Le comité de pilotage de l'étude comprend les Dr Hodane Yonis (Médecine Intensive Réanimation, Hôpital de la Croix-Rousse), Laurent Bitker (Médecine Intensive Réanimation, Hôpital de la Croix-Rousse), Mehdi Mezidi (Médecine Intensive Réanimation, Hôpital de la Croix-Rousse), Muriel Rabilloud (Service de Biostatistique, HCL) et Jean-Christophe Richard (Médecine Intensive Réanimation, Hôpital de la Croix-Rousse).

1.9.2. Comité de surveillance indépendant

Un comité de surveillance Independent sera constitué. Il sera composé de 2 experts français de la ventilation artificielle et d'un biostatisticien.

1.9.3. Autres comités

Non applicable

2 JUSTIFICATION SCIENTIFIQUE

2.1 Etat actuel des connaissances - Rationnel

Le syndrome de détresse respiratoire aiguë (SDRA) est la complication la plus sévère de la pneumonie à Covid-19, et a été observée chez 42% des patients de la cohorte de Wuhan [3]. Le management du SDRA secondaire à une pneumonie à Covid-19 repose sur la ventilation mécanique en réanimation, jusqu'à la guérison de l'agression pulmonaire, en l'absence de traitement étiologique validé à ce jour. Toutefois, la mortalité du SDRA compliquant une pneumonie à Covid-19 est extrêmement élevée (52% dans la cohorte de Wuhan [3], 67% dans la cohorte de l'état de Washington [4]). Une des caractéristiques de ce SDRA est la durée de ventilation mécanique particulièrement longue (14 jours en médiane dans la série de 29 patients intubés de notre service [5]), ce qui fait que ces patients sont particulièrement exposés aux effets délétères potentiels de la ventilation mécanique. Ces effets délétères pourraient être impliqués dans la mortalité importante du SDRA à Covid19, de façon similaire au SDRA secondaire à d'autres étiologies. Il a en effet été démontré indubitablement qu'un volume courant (VT) excessif à 12 ml/kg de poids idéal au cours du SDRA, était associé à une surmortalité absolue de l'ordre de 10%, en comparaison avec un VT à 6 ml/kg de PPT [6], qui est devenu depuis le standard of care en ventilation mécanique. Cette étude a par ailleurs fait adopter la calibration du volume courant en fonction d'un poids idéal (basé sur la taille des patients) plutôt que sur leur poids, dans la mesure où le volume pulmonaire aéré disponible pour recevoir le VT est bien mieux prédit par la taille, que par le poids. Il est toutefois probable que ce VT de 6 ml/kg soit encore excessif chez une part substantielle de patients avec SDRA. En effet, malgré une ventilation supposée protectrice (associant petit VT réglé à 6 ml/kg de poids idéal et contrôle de la pression plateau en dessous de 28-30 cm H_2O), 30% des patients avec SDRA présentent des signes anormaux d'hyperinflation intracycle (c'est-à-dire une élévation anormale de l'aération pulmonaire en fin d'insufflation), associés à une élévation des cytokines pro-inflammatoires sur leur liquide de lavage bronchiolo-alvéolaire (LBA) et une durée de ventilation mécanique plus élevée [7]. Dans une étude observationnelle récente sur 482 patients avec SDRA [8], une augmentation de 1 ml/kg dans le VT appliqué à la phase précoce du SDRA était associé à une augmentation de 23% du risque de mortalité en réanimation, suggérant que des variations minimes du VT à la phase initiale du SDRA pouvait impacter le pronostic de la maladie. Réduire le volume courant de 6 à 3-4 ml/kg de poids idéal dans le cadre d'une ventilation ultraprotectrice, associée à une épuration extracorporelle de CO_2 (pour compenser l'élévation du gaz carbonique dans le sang artériel conséquence de la baisse du VT) est associé à une baisse des médiateurs pro-inflammatoires dans le LBA [9], et pourrait baisser la mortalité des patients les plus sévères avec $PaO_2/FiO_2 <$

200 mm de Hg, sur la base d'une analyse post-hoc d'un essai randomisé [10]. Les limites de la ventilation ultraprotectrice associée à l'épuration extracorporelle de CO2 sont les suivantes : faible niveau de preuve, faible disponibilité des épurateurs extracorporels de CO2, coûts très élevés des circuits devant être changés tous les 72 heures, nécessité d'anticoagulation importante compte-tenu des débits sang relativement faibles, iatrogénie liée à l'implantation des canules et au traitement anticoagulant, inapplicabilité de ces stratégies dans les pays émergents…

Dans le contexte spécifique des patients avec pneumonie à Covid-19 et SDRA, nous avons pu observer sur 2 des 4 patients étudiés avec un scanner quantitatif inclus dans un protocole de recherche observationnel (NCT03870009) la présence d'hyperinflation intracycle en quantité substantielle (Figure 1) suggérant que ces patients sont particulièrement à risque d'agression pulmonaire induite par la ventilation mécanique liée à un volume courant encore trop élevé, d'autant qu'ils sont ventilés très longtemps.

Nous avons récemment réalisé une étude pilote multicentrique, de type avant-après [11], sur 34 patients avec SDRA standard (hors Covid-19), visant à évaluer la faisabilité et la sécurité d'une ventilation ultraprotectrice sans épuration extracorporelle, tout en cherchant à maintenir le pH artériel au-dessus de 7,20 en augmentant la fréquence respiratoire et en minimisant l'espace mort instrumental. Dans cette étude, nous avons pu réduire le VT jusqu'à 4 ml/kg chez 65% des patients et jusqu'à 5 ml/kg chez 88% des patients, malgré l'inclusion de patients présentant des acidoses sévères (pH < 7,20 chez 9% des patients à l'inclusion). Cette stratégie a pu être appliquée pendant la totalité de l'évolution du SDRA, sans nécessité d'augmenter le traitement sédatif, et en observant un taux de patient avec cœur pulmonaire aigu plus faible qu'attendu (6% vs. plus de 20% attendu[1])

En ce qui concerne la sécurité, la mortalité à J90 de ces patients était de 41%, en dépit de l'inclusion de patients moribonds, mais 32% des patients de la série ont présenté des épisodes d'acidose mixte sévère transitoires, contrôlés par des adaptations secondaires des réglages ventilatoires. Ce risque serait probablement minimisé en ne proposant pas cette stratégie aux patients avec acidose sévère (pH < 7,21)

Notre hypothèse est que la ventilation ultraprotectrice sans épuration extracorporelle peut réduire la mortalité du SDRA compliquant une pneumopathie à Covid-19, en réduisant les effets délétères de la ventilation mécanique, comme la ventilation mécanique prolongée est à ce jour le seul traitement efficace pour maintenir ces patients en vie.

Figure 1. Scanners quantitatifs obtenus à l'expiration (haut) et à l'inspiration (bas) chez un patient avec SDRA et pneumonie à COVID-19.

Le codage couleur vert correspond à des voxels hyperinflatés (avec densité radiologique < - 900 unité Hounsfield) alors que les parties jaunes correspondent aux voxels normalement aérés (densité radiologique entre -900 et -500 unités Hounsfield) ou faiblement aérés (densité radiologique entre -100 et -500 unités Hounsfield). Chez ce patient, l'hyperinflation intracycle était mesurée à 22% du volume courant et prédominait nettement dans les régions pulmonaires antérieures.

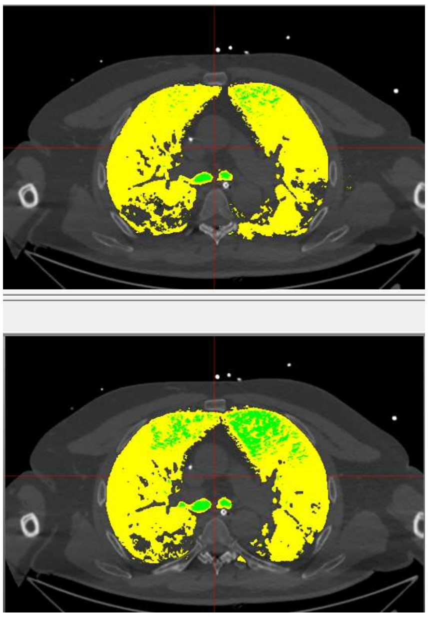

2.2 Hypothèse de la recherche

La ventilation ultraprotectrice sans circulation extracorporelle permet d'obtenir une probabilité supérieure à 50% que le résultat, basé sur la mortalité à J90 (critère prioritaire) et le nombre de jours vivant sans ventilation mécanique à J60 (second critère), soit favorable par rapport à la ventilation protectrice chez les patients présentant une pneumonie à COVID-19 et un SDRA modérément sévère à sévère.

2.3 Justification des choix méthodologiques

Il a été décidé de réaliser une étude randomisée contrôlée en groupe parallèle afin de fournir un niveau de preuve le plus élevé possible dans le contexte de l'urgence épidémique. Il n'est pas possible de réaliser d'aveugle dans la mesure où la connaissance des réglages ventilatoires est absolument nécessaire pour le clinicien en charge du traitement.
Le critère de jugement principal choisi (i.e. critère composite avec la mortalité priorisée, et le nombre de jours vivant sans ventilation mécanique en second critère) est accepté comme critère pertinent d'évaluation du SDRA et a été récemment utilisé dans un essai randomisé contrôlé au cours du SDRA dans une revue de haut niveau [12]. Il permet de réduire de façon considérable le nombre de sujets nécessaires pour répondre à la question par rapport à un critère principal basé sur la mortalité seule et répond à l'urgence de la situation épidémique actuelle. Il permet de hiérarchiser les critères pris en compte en classant les patients d'abord sur la mortalité puis sur le nombre de jours sans ventilation en l'absence de décès, et permet d'éviter l'écueil d'une réponse opposée en terme de bénéfice pour le patient sur les 2 composants (ex. mortalité plus basse mais nombre de jours vivant sans ventilation mécanique plus élevé dans un des bras) [13]. Une différence d'au moins un jour vivant sans ventilation mécanique a été choisie pour le 2ème composant du critère car elle est pertinente :

v. En terme d'accès à une ressource sous tension (la ventilation mécanique) dans le contexte épidémique car elle est nécessairement associée à une prolongation du séjour en réanimation, à la disponibilité d'un respirateur, et souvent à des drogues sédatives massivement utilisées dans le monde entier et en pénurie
vi. En terme d'expérience patient dans la mesure où la ventilation mécanique est extrêmement inconfortable et nécessite des aspirations trachéales pluriquotidiennes (un des gestes les plus douloureux en réanimation dans la grande étude européenne multicentrique sur la douleur et l'inconfort en réanimation [14])

2.4 Population cible

Les patients inclus seront les patients avec pneumonie à COVID-19 et SDRA [2], dans sa forme sévère et modérément sévère (i.e. présentant un rapport PaO2/FiO2 ≤ 150 mm Hg), dans les 48 premières heures suivant le diagnostic de SDRA, afin d'appliquer l'intervention le plus précocement possible, mais en laissant le temps d'organiser les formalités d'inclusion compte-tenu des contraintes liées à la charge en soin dans les services de réanimation dans le contexte épidémique.

2.5 Rapport bénéfices/risques

Bénéfices :
Les bénéfices individuels potentiels associés à l'application de la stratégie testée sont un meilleur contrôle de l'agression pulmonaire induite par la ventilation mécanique, une réduction de la durée de la ventilation mécanique, une sortie plus précoce de réanimation et un effet bénéfique sur la mortalité des patients.
Les bénéfices collectifs sont une réduction de la saturation des lits de réanimation dans le contexte pandémique, si la durée de ventilation et la durée de séjour en réanimation sont réduites par la ventilation ultraprotectrice.

Risques et contraintes :
Les risques associés à l'application de la stratégie testée ont été évalués dans une étude pilote [15] sur 34 patients qui a objectivé :
1. Un surrisque d'épisode d'acidose respiratoire sévère transitoire observé chez environ 30% des patients dans l'étude pilote [15], compte-tenu de la réduction du volume courant. Ces épisodes ont été rapidement contrôlés

par des adaptations ventilatoires incluant la ré-augmentation du VT. Ce risque sera par ailleurs franchement minimisé dans cette étude par la non-inclusion des patients avec pH < 7.21, dans la mesure où le pH à l'inclusion était un facteur de risque indépendant de développer un épisode d'acidose respiratoire sévère.
2. Une augmentation de la posologie du traitement sédatif. Ce risque théorique n'a pas été confirmé dans l'étude pilote [15], dans laquelle aucune variation de la posologie de sédation n'était détectée après l'inclusion. Par ailleurs, la protocolisation de l'arrêt de sédation dans la présente étude dès l'obtention d'un rapport $PaO_2/FiO_2 \geq 150$ mm de Hg en décubitus dorsal, telle qu'elle est réalisée dans les études randomisées les plus récentes [16, 17] aboutit à une réduction considérable du temps passé par les patients avec SDRA sous sédation.
3. Une dégradation de l'oxygénation en lien avec un dérecrutement secondaire à la réduction du volume courant. Cet effet n'a pas été retrouvé dans l'étude pilote (au contraire), en conséquence de l'augmentation du niveau de PEP [15]. Par ailleurs, dans l'étude ARMA [6], le rapport PaO_2/FiO_2 était significativement plus bas à J2 dans le groupe VT 6 ml/kg de PPT que dans le groupe VT 12 ml/kg de PPT (158 ± 73 vs. 176 ± 76 mm Hg), démontrant que les effets bénéfiques d'une stratégie ventilatoire sur l'oxygénation pouvaient être inversement reliés à la mortalité du SDRA. De plus, l'effet sur l'oxygénation en cas de baisse du VT de 6 à 4 ml/lg de PPT à PEP identique était modeste (-7 mm de Hg de PaO_2) dans une autre étude réalisée sur 10 patients [18]. Dans l'hypothèse où une dégradation modeste de l'oxygénation apparaitrait à la baisse du VT, nos données de l'étude pilote montre qu'elle serait aisément compensable par l'augmentation du niveau de PEP [15].
4. Une hyperinflation dynamique par le biais d'une augmentation de la PEP intrinsèque secondaire à l'augmentation de la fréquence respiratoire conséquence de la baisse du volume courant. Ce risque a été exclu dans l'étude pilote.
5. L'apparition d'un cœur pulmonaire aigu (défaillance ventriculaire droite en lien avec une hypertension artérielle pulmonaire responsable d'une élévation de la postcharge ventriculaire droite) en raison de l'effet vasoconstricteur de la $PaCO_2$ sur la circulation artérielle pulmonaire. Une association entre $PaCO_2$ et cœur pulmonaire aigu est en effet retrouvée en analyse univariée dans 3 des 4 grandes séries de patients avec SDRA où la présence d'un cœur pulmonaire aigu était systématiquement recherchée [19–22]. Toutefois, cette association ne persistait en analyse multivariée que dans 2 de ces études [19, 22] , alors qu'une troisième identifiait l'augmentation de la pression motrice comme seule variable respiratoire indépendamment associée au diagnostic de cœur pulmonaire aigu [21]. Enfin, dans l'étude de plus grande ampleur récemment publiée regroupant la plupart des patients des études précédentes [19, 21, 22] pour un total de 572 patients, $PaCO_2$ élevée, pression motrice élevée, hypoxémie et pneumopathie comme cause du SDRA étaient indépendamment liés à la survenue d'un cœur pulmonaire aigu [1].
Alors que ces données observationnelles ne permettent pas de conclure si ces associations sont liées à la gravité du SDRA ou aux réglages ventilatoires, on peut raisonnablement penser que la stratégie ventilatoire appliquée dans la présente étude pourrait théoriquement être responsable d'une augmentation de la postcharge ventriculaire droite secondaire à l'augmentation de la $PaCO_2$, qui sera compensée par un effet bénéfique lié à la baisse de la pression motrice. Cette hypothèse est argumentée par les résultats de notre étude pilote, qui n'a pas identifié de surrisque de cœur pulmonaire aigu, alors que ce diagnostic était systématiquement recherché en échocardiographie au cours des premières 48 heures après l'inclusion.
6. Une majoration de l'agression pulmonaire induite par la ventilation mécanique liée à l'augmentation de la fréquence respiratoire, conséquence de la baisse du volume courant. Cet effet délétère a été retrouvé de façon inconstante chez l'animal [23, 24] ; l'augmentation de la fréquence respiratoire étant parfois associée à une aggravation de l'agression pulmonaire induite par la ventilation mécanique à haut volume [24], alors que d'autres études ont objectivé des résultats inverses [25–27]. Chez les patients avec SDRA, la fréquence respiratoire n'est pas indépendamment liée avec la mortalité dans une large étude multicentrique prospective récente ayant inclus 482 patients ventilés de façon protectrice [8]. Elle n'a pas non plus été identifiée comme indépendamment liée avec la mortalité du SDRA dans les études observationnelles multicentriques de grande ampleur plus anciennes [28, 29]. Par ailleurs, la fréquence respiratoire à J2 était significativement plus élevée dans le groupe VT 6 ml/kg (28 ± 7/min) que dans le groupe VT 12 ml/kg (17 ± 6/min) dans l'étude ARMA [6, 30], alors que la mortalité était significative réduite en faveur du groupe petit VT. Enfin, l'étude pilote n'a pas identifié d'augmentation de la puissance transmise au poumon par le ventilateur [15], alors que sa composante liée au volume courant baissait significativement à VT 4 ml/kg, suggérant au pire l'absence d'effet délétère de l'augmentation de la fréquence, au mieux un effet favorable.
Au total, l'ensemble des données disponibles ne laisse pas suspecter d'effets délétères liés à l'augmentation de la fréquence respiratoire chez les patients inclus dans l'étude.

Contraintes
Les visites seront réalisées quotidiennement dans le service de réanimation pendant tout le séjour.
Il n'y a aucun prélèvement biologique supplémentaire lié à l'étude, en plus de ceux réalisés dans le cadre du soin
Il n'y a aucun examen radiologique supplémentaire lié à l'étude, en plus de ceux réalisés dans le cadre du soin
Un appel téléphonique sera réalisé à J365 de l'inclusion pour les questionnaires de qualité de vie, d'évaluation des troubles cognitifs et du syndrome de stress post-traumatique. Cet appel durera environ 45 minutes.

Evènements indésirables graves attendus
Le seul évènement indésirable attendu véritablement documenté dans l'étude pilote est une acidose respiratoire sévère, qui pourra être rapidement contrôlé par une adaptation ventilatoire [15].
Tous les évènements indésirables graves seront signalés aux promoteurs en temps réel. Il n'est pas attendu d'évènement indésirable tardif.

Au total, la balance bénéfice/risque ne paraît pas défavorable.

2.6 Retombées attendues

L'étude pourrait permettre une avancée thérapeutique majeure dans le traitement des formes graves de pneumonie à Covid-19 avec SDRA, pour lequel il n'existe actuellement aucun traitement étiologique, et où le seul traitement efficace est la ventilation artificielle dans l'attente de la guérison. Dans cette optique, la réduction de l'agression pulmonaire induite par la ventilation mécanique (VILI) est un objectif majeur, qui pourrait être démontré en cas de positivité de l'étude. Cette diminution du VILI, si elle est avérée, devrait réduire la fréquence des défaillances d'organe extra-respiratoire notamment rénale, qui ont un poids très fort dans la mortalité du SDRA. Par ailleurs, on attend une réduction du barotraumatisme (identifié chez 5 à 10% des patients sous la forme de pneumothorax en dépit de la ventilation protectrice), et de la durée de séjour en réanimation (un paramètre critique dans le contexte d'une saturation des lits de réanimation en condition épidémique).
Cette étude va aussi permettre d'évaluer l'impact à long terme de l'hypercapnie permissive sur la qualité de vie, le syndrome de stress post-traumatique et les altérations cognitives post-réanimation qui sont extrêmement fréquents. Enfin, dans le contexte plus global de maladies infectieuses émergentes à tropisme respiratoire, cette étude pourrait avoir un impact majeur en termes de santé publique pour les raisons suivantes :

- Le nombre de patient ventilés avec SDRA (c'est-à-dire à risque d'agression pulmonaire induite par la ventilation mécanique) est amené à augmenter considérablement par plusieurs ordres de magnitude
- La stratégie testée est adaptable à des pays en voie de développement ou émergents dans la mesure où elle ne nécessite aucune technologie additionnelle (comme la circulation extracorporelle), et repose seulement sur des adaptations ventilatoires.

3 OBJECTIFS DE LA RECHERCHE

3.1 Objectif principal

Evaluer le bénéfice de la ventilation ultraprotectrice en comparaison avec la ventilation protectrice sur un critère de jugement composite incluant la mortalité à J90 comme critère prioritaire et le nombre de jours vivants sans ventilation mécanique à J60 comme second critère

3.2 Objectifs secondaires

Objectifs d'efficacité :

1. Tester si la ventilation ultraprotectrice est associée à une baisse de la mortalité toute cause à J90 en comparaison avec le groupe contrôle (analyse en intention de traiter)

2. Tester si la ventilation ultraprotectrice est associée à une augmentation du nombre de jours vivant sans ventilation mécanique à J60 en comparaison avec le groupe contrôle
3. Tester si la ventilation ultraprotectrice est associée à une baisse de la mortalité toute cause à J90 en comparaison avec le groupe contrôle (analyse per protocole)
4. Tester si la ventilation ultraprotectrice est associée à une diminution du temps entre inclusion et extubation avec succès en comparaison avec le groupe contrôle
5. Tester si la ventilation ultraprotectrice est associée à une diminution de la durée de séjour hospitalière depuis l'inclusion en comparaison avec le groupe contrôle
6. Tester si la ventilation ultraprotectrice impacte les paramètres ventilatoires suivants mesurés quotidiennement pendant les 14 premiers jours suivant l'inclusion, en comparaison avec le groupe contrôle (rapport entre la pression partielle en oxygène dans le sang artériel et la fraction inspirée en oxygène (PaO2/FiO2), pH, pression partielle en CO2 dans le sang artériel (PaCO2), pression expiratoire positive (PEP), volume courant (VT), PEP totale, pression de crête, pression plateau, pression de travail, fréquence respiratoire, temps inspiratoire)

Objectifs de sécurité

7. Tester si la ventilation ultraprotectrice n'est pas associée à une augmentation des doses de sédation mesurées quotidiennement pendant les 14 premiers jours suivant l'inclusion, en comparaison avec le groupe contrôle
8. Tester si la ventilation ultraprotectrice est associée à une réduction du taux de recours aux thérapeutiques de sauvetage au cours des 14 premiers jours suivant l'inclusion (décubitus ventral, curares, monoxyde d'azote inhalé, manœuvres de recrutement, ECMO)
9. Tester si la ventilation ultraprotectrice est associée à une réduction de la densité d'incidence des effets indésirables suivants, en comparaison avec le groupe contrôle : acidose mixte sévère, pneumonie acquise sous ventilation mécanique, cœur pulmonaire aigu, barotraumatisme, ou tout effet indésirable grave)

Objectifs centrés patient

10. Tester si la ventilation ultraprotectrice n'est pas associée à une dégradation des performances cognitives à J365 de l'inclusion, en comparaison avec le groupe contrôle
11. Tester si la ventilation ultraprotectrice n'est pas associée à une dégradation de la qualité de vie à J365 de l'inclusion, en comparaison avec le groupe contrôle
12. Tester si la ventilation ultraprotectrice n'est pas associée à une augmentation du taux de patients avec syndrome de stress post-traumatique à J365 de l'inclusion, en comparaison avec le groupe contrôle

Objectifs d'efficience

13. Evaluer l'efficience de la ventilation ultraprotectrice, en comparaison avec le groupe contrôle à J90 de l'inclusion

4 CONCEPTION DE LA RECHERCHE

4.1 Type d'étude

Etude multicentrique inter-régionale, prospective, de supériorité, ouverte, randomisée et contrôlée, avec 2 groupes parallèles et randomisation équilibrée avec un ratio 1 pour 1.
Cette étude se qualifie comme une étude interventionnelle de catégorie 1.

4.2 Méthode pour la randomisation

La randomisation sera centralisée. La liste de randomisation sera générée sur le logiciel Clinsight®, et le bras de traitement auquel le patient est alloué ne sera révélé à l'investigateur qu'après inclusion dans l'étude.
La liste de randomisation est établie par le biostatisticien du Laboratoire Biostatistique-Santé. Elle est conservée pendant 25 ans dans une enveloppe inviolable avec les mentions obligatoires (signature du responsable de la liste, date et numéro de version, titre complet et code du protocole de recherche). Cette liste est transmise au Data Manager responsable du logiciel Clinsight® pour intégration.

Les patients seront randomisés par ordinateur via le logiciel Clinsight® par l'investigateur ou une personne désignée par l'investigateur, dans chacun des bras.

Une stratification par centre sera réalisée.

4.3 Critères de jugement

4.3.1 Critère de jugement principal

Le critère de jugement principal est un score composite incluant la mortalité à J90 comme critère prioritaire et le nombre de jours sans ventilation mécanique à J60 comme second critère, obtenu en comparant chaque patient d'un groupe, à l'ensemble des patients de l'autre groupe [12, 31]. Pour chaque paire, une valeur de +1 (favorable), -1 (défavorable) ou 0 (neutre) sera attribuée à chaque individu. Pour les paires avec un patient vivant et un patient décédé à J90, une valeur de +1 sera attribuée au patient vivant et une valeur de -1 au patient décédé. Si les 2 patients de la paire survivent à J90, le patient avec le plus de jours sans ventilation mécanique à J60 se verra attribuer une valeur de +1 ; et celui avec le moins de jours se verra attribuer une valeur de -1. Si les 2 patients de la paire survivent avec une différence de jours vivant sans ventilation mécanique < 1 jour, ou si les 2 patients de la paire meurent, une valeur de 0 sera attribuée à chacun. Pour chaque patient, le score correspondra à la somme des valeurs qui lui auront été attribuées lors de la comparaison avec tous les patients de l'autre groupe.

4.3.2 Critères de jugement secondaires

1. Mortalité toute cause à J90 en intention de traiter dans les 2 bras
2. Nombre de jours vivant sans ventilation mécanique à J60 (VFD-J60) dans les 2 bras
   Les VFD-J60 J60 sont calculés de la façon suivante [13] à partir du jour de randomisation :
   - VFD = 0 si le patient meurt entre la randomisation et J60 inclus
   - VFD = 60 – x si le sujet est extubé avec succès x jours après la randomisation ; l'extubation avec succès étant une extubation sans ré-intubation dans les 48 heures (ou l'arrêt de la ventilation invasive pendant plus de 48 heures chez les patients trachéotomisés)
   - VFD = 0 si le patient est sous ventilation mécanique invasive pendant plus de 60 jours
3. Mortalité toute cause à J90 per protocole dans les 2 bras
   Les patients du bras intervention seront considérés comme effectivement sous ventilation ultraprotectrice si le volume courant journalier moyen entre la randomisation et l'arrêt de la sédation lourde (et au maximum J14) est inférieur ou égal à 4,2 ml/kg de poids prédit par la taille (PPT)
4. Temps entre randomisation et extubation avec succès définie comme une extubation sans ré-intubation dans les 48 heures (ou arrêt de la ventilation invasive pendant plus de 48 heures chez les patients trachéotomisés) dans les 2 bras
5. Durée de séjour hospitalière définie comme l'intervalle entre la randomisation et la sortie de l'hôpital dans les 2 bras
6. Paramètres respiratoires suivants mesurés de façon journalière entre l'inclusion et l'arrêt de la sédation lourde (et au maximum J14) dans les 2 bras (PaO2/FiO2, pH, PaCO2, PEP, VT, PEP totale, pression de crête, pression plateau, pression de travail, fréquence respiratoire, temps inspiratoire)
7. Dose de benzodiazépines, de propofol, d'opiacés exprimée en équivalent morphine (1µg de sufentanil = 10 µg de fentanyl = 1 mg de morphine) [32] entre l'inclusion et J14. Les traitements antalgiques non opiacés ne seront pas pris en compte, de façon cohérente avec la littérature sur le sujet [32, 33].
8. Taux de recours aux thérapeutiques de sauvetage au cours des 14 premiers jours suivant l'inclusion dans les 2 bras (décubitus ventral, curares, monoxyde d'azote inhalé, manœuvres de recrutement, ECMO)
9. Densité d'incidence dans les 2 bras des effets indésirables suivants :
   a. acidose sévère (définie par un pH<7.15 et une PaCO2>45 mm Hg),
   b. pneumonie acquise sous ventilation mécanique
   c. cœur pulmonaire aigu défini en échocardiographie par l'association d'un ratio surface du ventricule droit sur gauche > 0,6 et d'un trouble de la cinétique septale

   d. barotraumatisme (pneumothorax, ou pneumomédiastin, ou emphysème sous cutané ou pneumatocèle de plus de 2 cm en imagerie)
   e. tout effet indésirable grave
10. Score de trouble cognitif T-MOCA dans les 2 bras (MOntreal Cognitive Assessment) évalué par téléphone à J365 de l'inclusion. Ce score varie de 0 à 30 (score normal à 26 ou plus)
11. Score de qualité de vie SF-36 évalué par téléphone à J365 de l'inclusion
12. Score de stress post-traumatique IES-R dans les 2 bras à J365 de l'inclusion
13. Ratio coût efficacité de la stratégie de ventilation ultraprotectrice en comparaison du groupe contrôle à J90 de l'inclusion

5 CRITERES D'ELIGIBILITE

5.1 Critères de pré-inclusion

Non applicable

5.2 Critères d'inclusion

1. Adulte âgé d'au moins 18 ans
2. Intubation et ventilation mécanique
3. Pneumonie à COVID-19 confirmée par RT-PCR sur prélèvement nasopharyngé ou du tractus respiratoire datant de moins de 7 jours
4. Insuffisance respiratoire aiguë non complètement expliquée par une insuffisance ventriculaire gauche ou une surcharge hydrosodée
5. Opacités radiologiques pulmonaires bilatérales non complètement expliquées par des épanchements pleuraux ou atélectasies ou des nodules
6. Ventilation mécanique invasive avec $PaO_2/FiO_2 \leq 150$ mm Hg et PEP ≥ 5 cm H_2O avec un VT ≤ 6 ml/kg de PPT
7. Sédation intraveineuse continue dans le cadre du traitement du SDRA

5.3 Critères de non inclusion

Critères relatifs à l'histoire de la maladie

1. Ventilation mécanique invasive ou non-invasive depuis plus de 48 heures (oxygénothérapie à haut débit autorisée sans limite)
2. Patient précédemment inclus dans le même protocole de recherche

Critères relatifs à la sévérité de la maladie

3. pH artériel < 7.21 malgré une fréquence respiratoire à 35/min au moment de l'inclusion
4. Traitement par assistance extracorporelle (ECMO ou épuration de CO2)
5. Pneumothorax ou fistule broncho-pleurale

Critères relatifs aux pathologies associées entraînant des risques particuliers

6. Hypertension intracrânienne (suspectée ou confirmée)

Critères relatifs aux comorbidités

7. BPCO connue définie par un score de GOLD ≥ 3
8. Insuffisance respiratoire chronique avec indication d'oxygénothérapie au long cours ou assistance ventilatoire au long cours (hypoxémie chronique)
9. Obésité morbide définie par un poids supérieur à 1 kg/cm
10. Drépanocytose
11. Greffe de moelle récente, aplasie post-chimiothérapie

12. Brûlure étendue (> 30% de la surface corporelle)
13. Cirrhose hépatique grave (Child-Pugh C)
14. Décision de limitation des thérapeutiques actives

    Critères relatifs à la réglementation

15. Patient se trouvant en période d'exclusion suite à la participation à une autre recherche impliquant la personne humaine de catégorie 1 OU inclus dans une recherche impliquant la personne humaine de catégorie 1, OU inclus dans une recherche impliquant la personne humaine partageant le même critère de jugement principal que la présente étude.
16. Grossesse ; Femmes allaitantes
17. Patient majeur protégé au sens de la loi
18. Patient non bénéficiaire d'un régime de sécurité sociale
19. Consentement de participation non obtenu (soit auprès du patient lui-même, soit auprès d'un de ses proches, soit enfin auprès de la personne de confiance que le patient aurait préalablement désigné par écrit) sauf si recours à la procédure d'urgence en l'absence de proche

5.4 Critères de sortie prématurée

La participation du patient à l'étude s'arrête si le patient (ou la personne de confiance) retire son consentement. Dans le cas où l'investigateur interrompt temporairement ou définitivement la participation à l'étude pour toute raison qui servirait au mieux les intérêts du patient en particulier en cas d'événements indésirables graves suspects d'être en lien avec la stratégie ventilatoire testée, le patient reste dans l'analyse pour respecter le principe de l'intention de traiter, et ses données sont collectées jusqu'à J90.

5.5 Inclusions concomitantes

Il est possible d'inclure les patients de l'étude dans des essais thérapeutiques médicamenteux sous réserve qu'ils n'interfèrent pas avec la stratégie ventilatoire testée ET qu'ils ne soient pas de catégorie 1. En effet, la prise en charge optimale des patients de réanimation avec SDRA à COVID-19 ne peut être que globale (associant le traitement étiologique (antiviral ou immunomodulation) et une optimisation de la ventilation mécanique). Dans la mesure où il est urgent d'avoir des études à fort niveau de preuve sur les 2 aspects, il n'est pas licite d'empêcher la co-inclusion des patients de la présente étude avec un protocole médicamenteux, sous réserve qu'il n'existe pas de période d'exclusion pour le protocole associé, qu'il n'interfère pas avec la stratégie ventilatoire testée, et qu'il ne partage pas le même critère de jugement principal que la présente étude. De plus, il ne nous a pas semblé éthique ne pas pouvoir répondre à une question majeure de santé publique dépassant la réanimation, en empêchant l'accès à des traitements antiviraux ou immunomodulateurs à l'essai pour les patients de l'étude.

5.6 Modalités de recrutement et faisabilité

L'épidémie de SARS-cov-2 est actuellement active en France, avec un afflux massif de patients dans les réanimations. La plupart des centres de l'étude sont complètement dédiés à la prise en charge des patients avec pneumonie à Covid-19, et ont tous augmentés leurs capacités de lits, ce qui va assurer un taux d'inclusion très élevé. La durée prévue de la première vague de l'épidémie est de plusieurs semaines malgré le confinement [34] ; les modélisations de l'impérial collège (utilisées pour décider du confinement en France) projetant une durée d'épidémie (malgré le confinement) jusqu'en juillet 2020 [35]. Il est par ailleurs anticipé la survenue de vagues consécutives de moindre importance.
Sur cette base, il est probable que l'étude, si elle débute avant le pic de l'épidémie, permettra l'inclusion très rapide d'une dizaine de patients par centre et par mois, soit l'inclusion de 200 patients (nombre de sujets nécessaires total dans le projet) en 2 mois. Cette hypothèse d'inclusion mensuelle est conservative car la quasi-totalité des services participants a doublé ses capacités en lits. Toutefois et de façon conservative, il a été décidé d'annoncer une durée d'étude de 12 mois, en faisant l'hypothèse que le rythme d'inclusion puisse être gêné par un afflux de patients. La dynamique de l'épidémie permettant d'inclure des patients sur une durée bien plus longue que 2 mois.

Tableau 1. Recrutement attendu par centre

Nom de l'IP	Prénom de l'IP	Ville	Pays	Recrutement attendu par mois
AUBRUN	Frederic	Lyon	France	10
ARGAUD	Laurent	Lyon	France	10
FRIGGERI	Arnaud	Oullins	France	10
DELANNOY	Bertrand	Lyon	France	5
MULLER	Michel	Annecy	France	5
POMMIER	Christian	Lyon	France	5
YONIS	Hodane	Lyon	France	30
RIMMELE	Thomas	Lyon	France	10
DUPUIS	Claire	Clermont-Ferrand	France	5
TERZI	Nicolas	Grenoble	France	10
THIERY	Guillaume	Saint-Etienne	France	10

IP= investigateur principal

En ce qui concerne la faisabilité, une étude pilote multicentrique a justement été réalisée pour répondre à cette question [14].

Les résultats montrent :

- que le volume courant médian passe de 6 ml/kg à l'inclusion à 4,1 ml/kg de PPT à J2 de l'étude
- que 65% des patients à J2 ont un VT<4,2 ml/kg en dépit de l'inclusion de 9% de patients avec acidose sévère à l'inclusion (pH≤7,20), ce qui a justifié l'utilisation d'une valeur de pH inférieure à 7,21 comme critère de non-inclusion (afin d'augmenter la probabilité d'obtenir une ventilation à bas volume dans le bras intervention)
- que 88% des patients à J2 ont un VT<5,2 ml/kg

Un élément important à signaler est que cette étude, a été réalisée avec des centres académiques et non-académiques, la plupart non-experts en ventilation artificielle, ce qui montre l'applicabilité de la stratégie dans un contexte relativement large.

6 TRAITEMENTS EXPERIMENTAUX / STRATEGIES EXPERIMENTALES

6.1 Traitement à l'étude

- **Définition et description de la stratégie / procédure**

  La stratégie à l'étude est une stratégie conforme aux recommandations internationales [2] avec comme seule différence une réduction du volume courant avec comme cible 4 ml/kg de PPT.

  Les réglages initiaux du respirateur après la randomisation sont les suivants :

  a. Utiliser le mode volume assisté contrôlé
  b. Enlever le raccord annelé et le remplacer par un raccord minimisant l'espace mort
  c. Humidifier les gaz inhalés préférentiellement avec un humidificateur chauffant selon les recommandations françaises de prise en charge du SDRA [36].
  d. Réduire le VT de 1 ml/kg de PPT par intervalles ≤ 2heures jusqu'à 4 ml/kg de PPT
  e. Ajuster la fréquence respiratoire pour maintenir le volume minute à son niveau d'avant inclusion jusqu'à un maximum de 35/min.
  f. I/E réglé entre 1/2 et 1/4 pour minimiser la PEP intrinsèque
  g. Ajuster la PEP selon la table PEP-FiO2 (Tableau 2)
  h. Ajuster éventuellement le VT pour obtenir l'objectif de pression de plateau

Tableau 2. Table PEP-FiO_2 [6]

PEP (cm H_2O)	5	5	8	8	10	10	10	12	14	14	14	16	18	20	22	24
FiO_2 (%)	30	40	40	50	50	60	70	70	70	80	90	90	90	100	100	100

FiO_2 = fraction inspirée en oxygène ; PEP = pression expiratoire positive.

- **Justification de la stratégie / procédure choisie**
  Cette stratégie a été testée dans une étude pilote évaluant sa faisabilité et sa sécurité [15]
- **Description des contre-indications**

En cas d'apparition d'une hypertension intracrânienne, la stratégie de ventilation ultraprotectrice à l'étude n'est plus appliquée. Le patient reste dans son groupe pour l'analyse en intention de traiter

6.2 Traitement de comparaison

Le traitement comparateur est la prise en charge habituelle utilisant un volume courant de 6 ml/kg de PPT (ventilation protectrice) suivant les recommandations des experts français [37]. Les réglages initiaux du respirateur après la randomisation sont les suivants :

a. Utiliser le mode volume assisté contrôlé
b. Régler le VT à 6 ml/kg de PPT
c. Humidifier les gaz inhalés préférentiellement avec un humidificateur chauffant selon les recommandations françaises de prise en charge du SDRA [36].
d. Ajuster la fréquence respiratoire pour maintenir le volume minute à son niveau d'avant inclusion jusqu'à un maximum de 35/min.
e. I/E réglé entre 1/2 et 1/4 pour minimiser la PEP intrinsèque
f. Ajuster de la PEP selon la table PEP-FiO2 (Tableau 2)
g. Ajuster éventuellement le VT pour obtenir l'objectif de pression de plateau

6.3 Insu

Il n'y a pas de possibilité de réaliser d'insu dans la mesure où la connaissance des réglages ventilatoires et la connaissance des résultats de la gazométrie artérielle (qui peut donner des informations sur le bras de randomisation) est absolument nécessaire pour le clinicien en charge du traitement. L'évaluation du critère de jugement principal se fera par des techniciens de recherche clinique qui ne seront pas impliqués dans la prise en charge du patient. Par ailleurs, la protocolisation de la ventilation mécanique, des traitements du SDRA, et du sevrage respiratoire permettra de standardiser la prise en charge dans les 2 groupes en minimisant les biais.

6.4 Traitements associés autorisés et interdits

La prise en charge du SDRA sera standardisée dans les 2 bras de la façon suivante. L'algorithme présenté à été utilisé dans l'étude pilote multicentrique [15] incluant des centres académiques et non académiques.

Prise en charge ventilatoire dans les 2 groupes

Les objectifs thérapeutiques ventilatoires dans les 2 bras sont conformes aux modalités actuelles de prise en charge du SDRA [16, 17]:
- Pression plateau (Pplat) mesurée par une occlusion télé-inspiratoire de 3 secondes ≤ 30 cm H2O
- PaO2 comprise entre 60 et 80 mm Hg ou SpO2 comprise entre 88% et 95% avec les valeurs de PaO2 prioritaires sur les valeurs de SpO2
- pH artériel compris entre 7,20 et 7,45

Les alarmes du respirateur seront réglées afin de détecter les asynchronies
- Alarme de VT réglée à 1,5 fois le VT en VAC afin de détecter les doubles déclenchements
- Alarme de fréquence respiratoire réglée à 37/min afin de détecter les auto-déclenchements, les désadaptations du respirateur, et les épisodes de polypnée au cours du sevrage

Traitements adjuvants à visée respiratoire dans les 2 bras

- Gestion de la curarisation
    i. Curarisation initiale pendant au moins 48 heures à la phase initiale du SDRA si $PaO_2/FiO_2 < 150$ mm Hg [2, 17]
    ii. Tentative d'arrêt quotidien ensuite si PaO2/FiO2 ≥ 100 et PEP ≤ 10 cm H2O
- première séance de décubitus ventral si $PaO_2/FiO_2 < 150$ mm Hg de 16 heures au minimum, et poursuite des séances de 16h/24H si $PaO_2/FiO_2 < 150$ mm de Hg **OU** PEP > 10 cm H_2O **OU** $FiO_2 > 60\%$ après le retour en décubitus dorsal [16].
- Gestion de la sédation
    i. La sédation est réalisée avec les produits utilisés habituellement selon la pratique du centre.
    ii. La sédation est utilisée avec un objectif de score RASS [38] entre -4 et -5 pendant les périodes de curarisation.
    iii. La sédation est utilisée avec un objectif de score RASS entre -4 et -5 en décubitus ventral.
    iv. La sédation est utilisée avec un objectif de score RASS entre –3 et -4, réévalué au moins toutes les 4 heures **jusqu'au succès de l'épreuve de sevrabilité de la sédation lourde**
    v. Dans tous les autres cas, le score de RASS cible se situe entre 0 et -2 jusqu'à l'extubation sauf exception (pathologie intracrânienne justifiant une sédation plus profonde par exemple sur décision du clinicien en charge du patient).

Tableau 3. Gestion de la sédation.

Situation clinique	Score RASS cible
**curarisation**	entre -4 et -5
**décubitus ventral**	entre -4 et -5
**sédation lourde**	entre -3 et -4
**après succès de sevrage de la sédation lourde**	entre 0 et -2

Epreuve de sevrage de la sédation lourde (Figure 2Erreur ! Source du renvoi introuvable.)

La sevrabilité de la sédation lourde sera testée quotidiennement à partir de J2 chez les patients avec $PaO_2/FIO_2 > 150$ mm Hg en décubitus dorsal de la façon suivante :

i. Arrêt des curares
ii. diminuer la PEP jusqu'à 5 cm H2O en 20 à 30 minutes, en réglant la FiO2 à 50%, **et en augmentant le VT à 6 ml/kg de PPT si applicable, en baissant la FR pour maintenir la ventilation minute constante,** et en réglant le I/E à 1/2**.**
iii. Si SpO2 < 88% plus de 5 minutes ou fréquence respiratoire (FR) > 35/min, retour aux réglages pré-test.
iv. Si après 30 minutes d'épreuve, $SpO_2 \geq 88\%$ (ou $PaO_2/FiO_2 \geq 150$ mm Hg si SpO_2 non disponible) avec FR ≤ 35/min, PEP 5 cm H_2O, FiO_2 50%, **la cible de RASS est alors 0 -2**
v. La sevrabilité du respirateur doit alors être recherchée quotidiennement (cf. ventilation des patients potentiellement sevrables), et le patient est ventilé en **VAC à 6ml/kg de PPT** ou en AI en cas de réponse aux ordres simples avec comme cible un VT entre 6 et 8 ml/kg de PPT
vi. Reprise de la sédation lourde avec les réglages du VT correspondant au groupe d'allocation :
    - En VAC
        - si asynchronies patient-machine (dépression de la courbe de pression à l'inspiration double déclenchement, désadaptation du respirateur) ET échec du passage en AI (cf.infra)
        - si fréquence respiratoire > 35/min ET échec du passage en AI
        - détresse respiratoire ET échec du passage en AI
    - en aide inspiratoire
        - si le VT est supérieur à 8 ml/kg de PPT malgré la baisse de la pression d'aide à 7 cm H2O
        - si fréquence respiratoire >35/min persistant après ajustement des réglages de l'AI
        - si détresse respiratoire persistant après ajustement des réglages de l'AI

Figure 2. Epreuve de sevrage de la sédation lourde

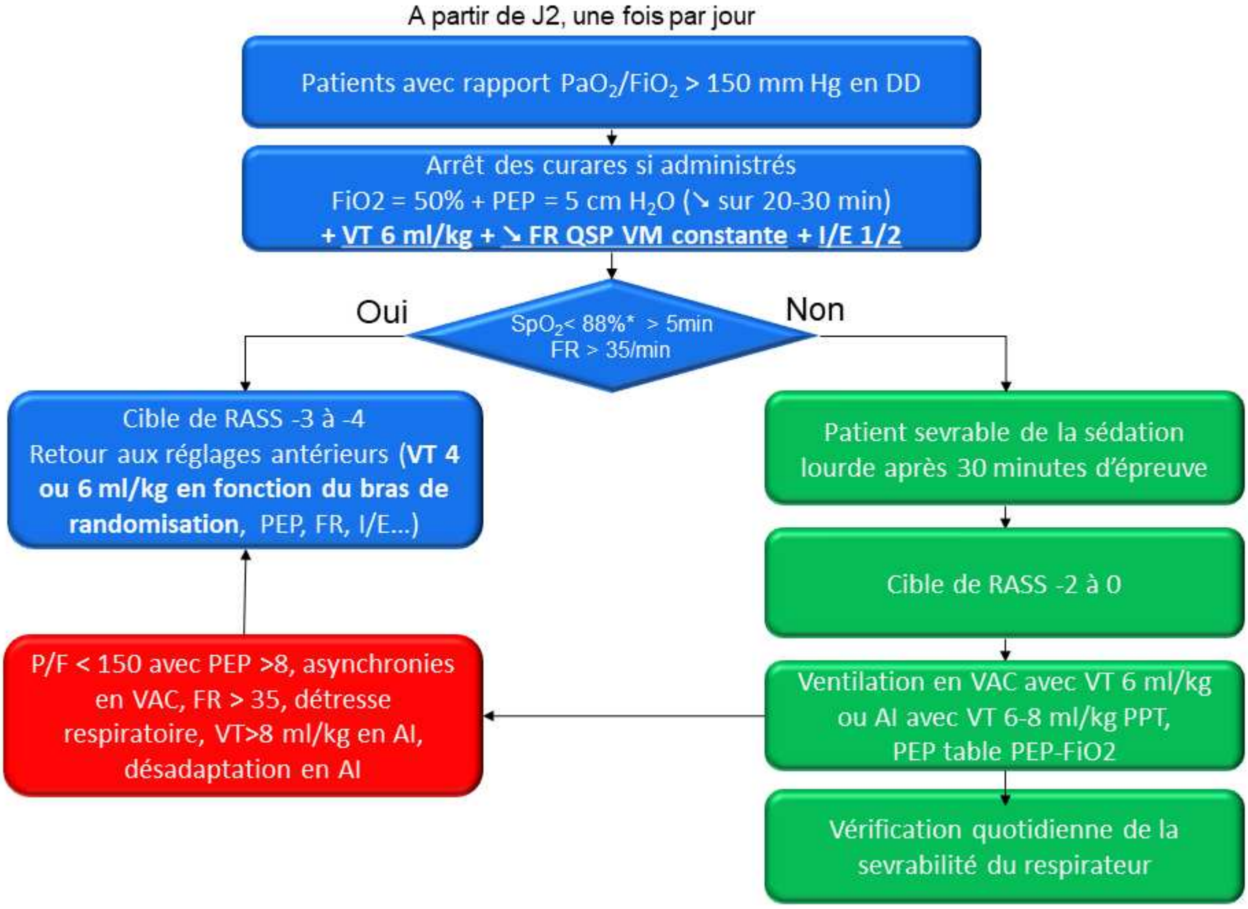

Adaptation de la prise en charge respiratoire de l'inclusion jusqu'à l'arrêt de la sédation lourde

- Si PaO_2 < 60 mm Hg ou SpO_2 < 88% (à réaliser dans l'ordre si persistance après chaque étape):
  a. gestion de la PEP et de la FiO_2 selon la table PEP-FiO_2 (Tableau 2), avec contrôle 15 minutes plus tard de la SpO_2.
  b. décubitus ventral si PaO_2/FiO_2 < 150 mm de Hg **OU** PEP > 10 cm H_2O **OU** FiO_2 > 60%
  c. réintroduction éventuelle des curares si PaO_2/FiO_2 < 150 mm Hg
  d. NO inhalé à la posologie de 10 ppm réévaluée 24 heures plus tard maximum
  e. manœuvres de recrutement non recommandées, laissées au libre choix du clinicien
  f. considérer l'ECMO
- Si PaO_2 > 80 mm Hg ou SpO_2 > 95% (à réaliser dans l'ordre si persistance après chaque étape):
  a. arrêt des manœuvres de recrutement si elles avaient été employées
  b. arrêt du NO inhalé s'il avait été employé
  c. arrêt des curares s'ils avaient été employés après au moins 48 heures d'administration
  d. gestion de la PEP et de la FiO_2 selon la table PEP-FiO_2 (Tableau 2), avec contrôle 15 minutes plus tard de la SpO_2
- Si Pplat > 30 cm H_2O (à réaliser dans l'ordre si persistance après chaque étape):
  a. Si efforts inspiratoires visibles
     i. reprise de la curarisation avec bolus initial
     ii. si Pplat > 30 cm H_2O après le bolus de curares : diminuer le volume courant par paliers de 1 ml/kg de PPT toutes les 5 minutes tant que Pplat > 30 cm H_2O jusqu'à 4 ml/kg de PPT au minimum (si le pH est < 7,2, le VT n'est pas diminué)
  b. Si absence d'efforts inspiratoires visibles
     i. diminuer le volume courant par paliers de 1 ml/kg de PPT toutes les 5 minutes tant que

Pplat > 30 cm H_2O jusqu'à 4 ml/kg de PPT au minimum (si le pH est < 7,2, le VT n'est pas diminué)

- Si pH < 7,20 (à réaliser dans l'ordre si persistance après chaque étape):
  a. adapter la sédation et la curarisation pour obtenir une bonne adaptation entre le patient et le ventilateur mécanique
  b. enlever le raccord annelé s'il est présent. Remplacer l'échangeur de chaleur et d'humidité s'il est présent par un humidificateur chauffant
  c. augmenter la fréquence respiratoire sans dépasser 35 cycles par minute.
  d. perfusion éventuelle de bicarbonates (dose et quantité à la discrétion du médecin en charge)
  e. si le pH reste < 7,15, augmenter le volume courant par palier de 1 ml/kg de PPT jusqu'à obtenir un pH ≥ 7,20 sans dépasser 8 ml/kg de PPT
  f. considérer l'épuration extracorporelle de CO_2 ou l'ECMO
- Si pH > 7,45 (à réaliser dans l'ordre si persistance après chaque étape):
  a. diminuer le VT à 4 ml/kg de PPT
  b. diminuer la fréquence respiratoire.
- Si VT > 4ml/kg de PPT et pH > 7,20 **dans le bras intervention** :
  a. Tenter de diminuer le VT par paliers de 1 ml/kg de PPT jusqu'à 4 ml/kg de PPT (au moins biquotidiennement)
- Si VT > 6ml/kg de PPT et pH > 7,20 **dans le bras contrôle** :
  a. Tenter de diminuer le VT par paliers de 1 ml/kg de PPT jusqu'à 6 ml/kg de PPT (au moins biquotidiennement)
- Si pneumothorax :
  a. Le niveau de PEP est laissé au libre choix du clinicien tant que le pneumothorax et/ou le drain thoracique est présent, les autres recommandations de l'étude devant s'appliquer.
- Si asynchronies patient-machine en VAC (dépression de la courbe de pression à l'inspiration, double déclenchement, désadaptation du respirateur) ou fréquence respiratoire > 35/min ou détresse respiratoire
  i. procédure diagnostic à la recherche de la cause de la détresse respiratoire aiguë.
  ii. Vérifier le réglage du trigger inspiratoire et la présence d'eau dans les tuyaux du respirateur
  iii. Si administration continue de curares : bolus de curares et augmentation de 20% de leur dose
  iv. Si PaO2/FIO2<150 mm Hg ou PEP > 8 cm H2O (à réaliser dans l'ordre si persistance après chaque étape):
     a. Bolus de sédation et de morphinique associé à une reprise de leur administration continue ou une augmentation de leur dose continue
     b. Bolus de curares et reprise de leur administration en continue
  v. Si PaO2/FIO2≥150 mm Hg avec PEP ≤ 8 cm H2O en décubitus dorsal (à réaliser dans l'ordre si persistance après chaque étape) :
     a. Ajuster le rapport I/E (réduire jusqu'à 1/4 si dépression de la courbe de pression à l'inspiration, allonger si double déclenchement sans aller au-delà d'un I/E à ½)
     b. Faire un test d'augmentation du VT jusqu'à 6 ml/kg de PPT **dans le bras intervention** (ne pas faire cette étape si déjà réalisée il y a moins de 24 heures)
     c. Bolus de sédation et de morphinique
     d. si persistance des asynchronies après 15 minutes, passage en Aide Inspiratoire avec un VT cible entre 6 et 8 ml/kg de PPT
     e. si VT > 8 ml/kg de PPT en aide inspiratoire ou fréquence respiratoire >35/min ou détresse respiratoire

        - nouveau bolus de sédation et de morphinique associé à une reprise de leur administration continue ou une augmentation de leur dose continue et **reprise de la sédation lourde** (objectif de RASS -3 à -4)

        - Réduire le VT à 4 ml/kg **dans le bras intervention**

f. si persistance des asynchronies après 15 minutes, bolus de curares et reprise de leur administration en continue

Ventilation des patients potentiellement sevrables du respirateur (Figure 3)

Les patients sont déclarés potentiellement sevrables du respirateur s'ils présentent l'ensemble des critères suivants :

- FiO_2 < 60%
- PEP = 5 cm H_2O
- SpO_2 ≥ 88%
- fréquence respiratoire ≤ 35/min
- réponse aux ordres simples obtenue

La présence de ces critères doit être recherchée quotidiennement et une épreuve de ventilation spontanée est alors réalisée selon les modalités en vigueur dans le centre (pièce en T, aide inspiratoire à 7 cm d'H_2O…).
En cas d'intolérance clinique lors de l'épreuve de ventilation spontanée (FR > 35/min, SpO_2 < 85%, trouble de la conscience, arythmie, fréquence cardiaque > 130/min, tirage, sueurs), les réglages antérieurs sont réappliqués et l'épreuve de sevrage sera réalisée quotidiennement jusqu'à réussite clinique.
En cas de réussite de l'épreuve de sevrage, la décision d'extubation, de trachéotomie, et de réaliser une VNI post-extubation est laissée au libre choix du clinicien.
En cas de ré-intubation après une extubation programmée, les réglages ventilatoires sont laissés au libre choix du clinicien.

Figure 3. Ventilation des patients potentiellement sevrables

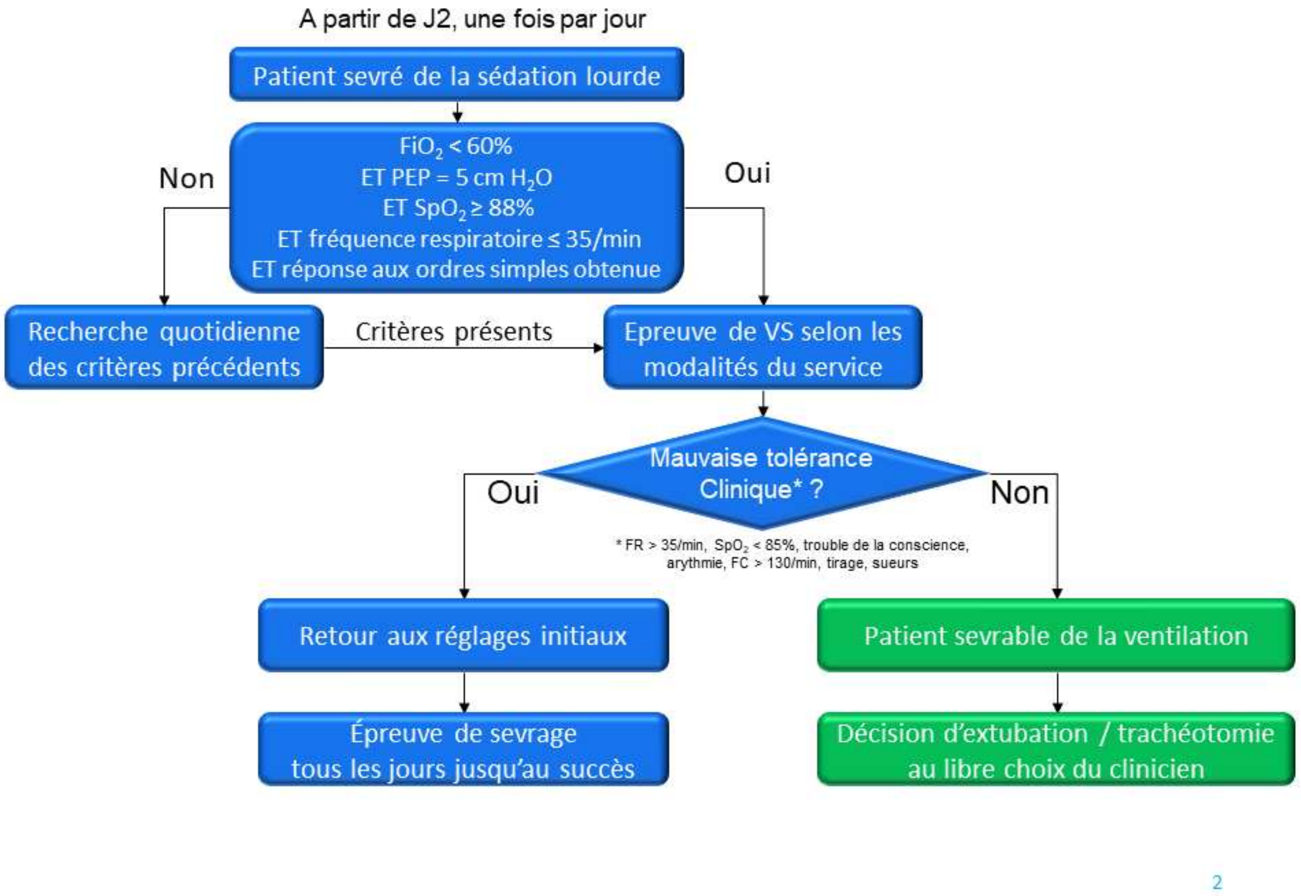

7 ORGANISATION GÉNÉRALE

7.1 Calendrier de l'étude

Durée de la période d'inclusion : 12 mois au maximum
Durée de la participation pour chaque patient : 365 jours ± 10 jours (intervalle de temps toléré pour la visite terminale)
Durée totale de l'étude : = 12 mois + 365 jours = 24 mois
Début des inclusions : T2 2020

Dès la première inclusion, le promoteur doit informer sans délai le CPP et l'ANSM s'il s'agit d'une étude du 1° de la date effective de démarrage de l'étude (date effective de démarrage = date de signature du consentement par la première personne qui se prête à la recherche).
La date de fin d'étude sera transmise par le promoteur au CPP et à l'ANSM pour les recherches du 1° dans un délai de 90 jours. La date de fin de la recherche correspond au terme de la participation de la dernière personne qui se prête à la recherche, ou le cas échéant, au terme défini dans le protocole.

7.2 Schéma général et tableau récapitulatif

Figure 4. Schéma de l'étude

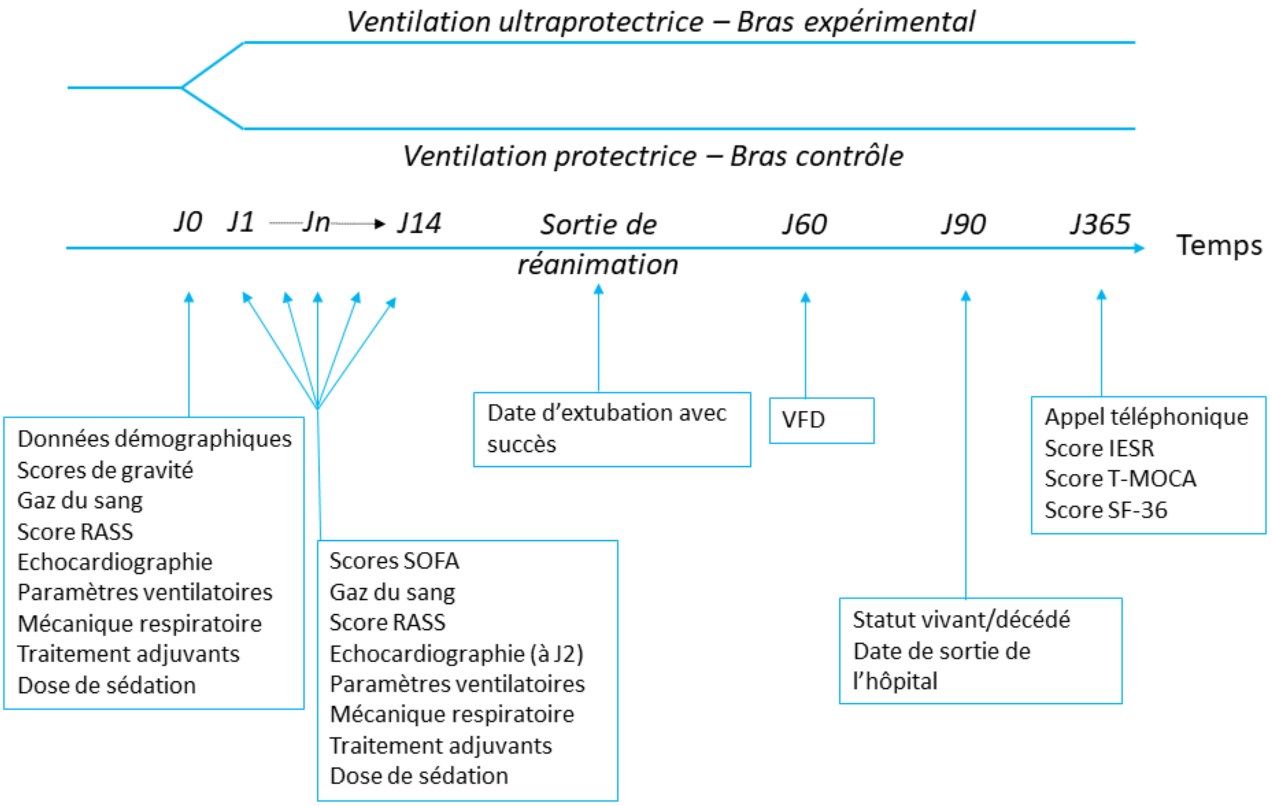

VFD= jour sans ventilation mécanique

Tableau récapitulatif comportant tous les examens du suivi.

Visites	Pré-inclusion (J-2-J0)	Inclusion (J0)	Entre H2 et H6	Entre H8 et H12	Entre H14 et H18	J1 à J14 *	J15 à J28 *	Sortie de réanimation	J60	J90 ± 2	J365 ± 10
Données démographiques	x										
Antécédents médicaux	x										
Radiographie thoracique < 48h	x										
Réglages ventilatoires adaptés	x										
PCR SARS-cov-2	x										
Gaz du sang < 4h	x										
Beta HCG ****	x										
Randomisation		x									
Score de Mc Cabe		x									
IGS II		x									
Score SOFA		x				x					
Poids mesuré		x				x					
Paramètres ventilatoires		x	x	x	x	x					
Paramètres hémodynamiques		x				x					
Score RASS		x	x	x	x	x					
Dose de sédation		x				x					
Echocardiographie		x				x **					
Traitements adjuvants du SDRA		x				x					
Gaz du sang		x	x	x	x	x					
Test de sevrabilité de la sédation lourde						x ***	x				
Epreuve de VS						x	x				
EIG	X	X	X	X	X	x	x	X	X	X	X
VFD									x		
Survie										x	
Score IESR †,*****											x
Score SF36 †,*****											x
Score T-MOCA†,*****											x

VFD = jours vivant sans ventilation mécanique ; EIG = événements indésirables graves ; VS = ventilation spontanée.

* chez les patients ventilés ; ** à J1 ; *** à partir de J2 ; **** chez les femmes en âge de procréer ; ***** entretien téléphonique ; † réalisé spécifiquement pour la recherche

-

7.3 Déroulement de l'étude

7.3.1 Screening – Pré-inclusion

Les patients susceptibles de participer à l'étude seront identifiés au sein de chaque service participant dans le cadre de leur suivi médical régulier. Les proches des patients seront informés par un médecin investigateur de l'existence du protocole (dans la mesure où les patients seront tous dans le coma artificiel). Toute explication nécessaire à la bonne compréhension de l'étude sera donnée aux proches, ainsi qu'une lettre d'information expliquant les objectifs et le déroulement du protocole. Le praticien remettra également un formulaire de consentement en double exemplaire. Il sera laissé le délai de réflexion nécessaire pour décider de sa participation à l'étude.

Le formulaire de consentement doit être signé avant la réalisation de tout examen clinique ou paraclinique nécessité par la recherche.

Si le proche donne son accord de participation, celui-ci et l'investigateur datent et signent nominativement (nom et prénom en clair) deux exemplaires du formulaire de consentement. L'un est conservé par le proche, l'autre est conservé dans le classeur investigateur de l'étude. »

Si le représentant du patient n'est pas présent lors du screening (cas très probable en raison des mesures de confinement), l'inclusion d'un patient éligible pourra être réalisée selon une procédure d'urgence. L'investigateur remplit alors un formulaire confirmant l'absence de présence des proches du patient sur le site. L'information des proches et le recueil de leur consentement (selon la procédure décrite ci-dessus) devra être réalisée dès que possible. En cas de refus de consentement par ses proches, le patient inclus selon la procédure d'urgence sera exclu de l'étude. Le formulaire de consentement (ou le formulaire d'inclusion selon la procédure d'urgence) doit être signé avant la réalisation de toute procédure nécessitée par la recherche.
Une notice d'information et un formulaire de confirmation de participation seront fournis au patient dès qu'il sera en état de donner son consentement. Le formulaire de confirmation de participation sera signé par le patient et une copie lui sera fournie. L'investigateur en conservera l'original. Toutefois, les patients inclus selon la procédure d'urgence, sans proche identifiable, et qui décèderaient sans avoir repris un état de conscience normale, resteront inclus dans l'étude.

Paramètres du screening pré-inclusion

- Récupérer les antécédents médicaux
- Récupérer la RT-PCR SARS-Cov-2 sur prélèvement nasopharyngé ou du tractus respiratoire
- Vérifier que le VT est réglé à une valeur ≤ 6 ml/kg de PPT avec une PEP ≥ 5 cm H2O
- Gaz du sang datant de moins de 4 heures avec FiO2 et PEP correspondant
- Dosage de beta-HCG pour les femmes en âge de procréer
- Radiographie thoracique ou scanner datant de moins de 48 heures
- Récupérer les résultats de l'échographie cardiaque du jour calendaire

Aucun de ces examens n'est spécifiquement réalisé dans le cadre de l'étude

7.3.2 Visite d'inclusion / Randomisation

Données d'admission

- date de naissance (mois/année)
- date d'admission à l'hôpital (jour/mois/année)
- date d'admission en réanimation (jour/mois/année)
- heure d'admission en réanimation (hh:mm)
- sexe
- taille (cm)
- poids mesuré à l'admission en réanimation (kg)
- score IGS II [39] à l'admission en réanimation
- score de Mac Cabe [40]
- origine du patient (domicile, service d'urgence, service de court séjour hors réanimation, service de réanimation, long séjour)
- immunodépression

Données relevées à l'inclusion

- date d'inclusion (jour/mois/année)
- heure d'inclusion (hh:mm)
- date d'intubation (jour/mois/année)
- heure d'intubation (hh:mm)
- facteurs de risque de SDRA parmi les suivants (plusieurs choix possibles) : COVID-19, pneumopathie communautaire, inhalation de liquide gastrique, pneumopathie acquise sous ventilation mécanique, pneumopathie associée aux soins, sepsis intra-abdominal, sepsis extra-abdominal et extrapulmonaire, pancréatite aiguë, contusion pulmonaire, noyade, inhalation de fumée toxique, polytransfusion, traumatisme thoracique, polytraumatisme, circulation extracorporelle, autre

- poids mesuré à l'inclusion (kg)
- paramètres ventilatoires :
  - mode ventilatoire (VAC/AI/APRV/BIPAP)
  - FIO_2 (%)
  - volume courant (ml)
  - fréquence respiratoire (/min)
  - niveau de PEP externe (cm H2O)
  - niveau de PEP totale du système respiratoire (cm H2O)
  - pression de crète du système respiratoire (cm H2O)
  - pression plateau du système respiratoire (cm H2O)
  - Ti/Ttot (1/x)
  - débit inspiratoire (l/min)
- paramètres hémodynamiques
  - dose de noradrénaline (µg/kg/min ou mg/h au choix du centre)
  - dose d'adrénaline (µg/kg/min ou mg/h au choix du centre)
  - dose de dobutamine (µg/kg/min ou mg/h au choix du centre)
- paramètres neurologiques
  - score RASS
  - score de Glasgow
  - dose de midazolam (mg/H
  - dose de propofol (mg/H
  - dose de morphine (mg/H)
  - dose de fentanyl (µg/H)
  - dose de sufentanil (µg/H)
  - type et dose de curare (molécule, dose en mg/h)
- données clinico-biologiques supplémentaires permettant de calculer le score SOFA [41] le jour de l'inclusion (plaquettes, bilirubine, créatinine, diurèse des dernières 24 heures)
- gaz du sang (PaO2, pH, PaCO2, bicarbonates, SaO2)
- position du malade pendant les gaz du sang (décubitus dorsal/décubitus ventral)
- lactates
- traitements adjuvants parmi les suivants au cours des dernières 24 heures (monoxyde d'azote inhalé, décubitus ventral, manœuvres de recrutement, épuration extrarénale, ECMO)
- paramètres échocardiographiques
  - rapport des surfaces ventriculaire droite et gauche
  - présence d'une dyskinésie septale

7.3.3 Visites de suivi

Les visites de suivies sont listées ci-dessous. Toutes les visites jusqu'à la sortie de réanimation correspondent à la prise en charge habituelle. Aucun bilan biologique n'est réalisé spécifiquement dans le cadre du soin
Données relevées à H4 (± 2H), H10 (± 2H), et H16 (± 2H) après l'inclusion

- date et heure de mesure
- paramètres ventilatoires :
  - mode ventilatoire (VAC/AI/APRV/BIPAP)
  - FIO_2 (%)
  - volume courant (ml)
  - fréquence respiratoire (/min)

    - niveau de PEP externe (cm H2O)
    - niveau de PEP totale du système respiratoire (cm H2O) si mesurable
    - pression de crète du système respiratoire (cm H2O)
    - pression plateau du système respiratoire (cm H2O) si mesurable
    - Ti/Ttot (1/x)
    - débit inspiratoire (l/min)
- score RASS
- gaz du sang (PaO2, pH, PaCO2, bicarbonates, SaO2)
- position du malade pendant les gaz du sang (décubitus dorsal/décubitus ventral)

Données relevées à J1 (24 heures (± 2H) après l'inclusion) puis le matin de chaque jour calendaire suivant J1 jusqu'à J14 chez les patients ventilés

- poids (kg)
- paramètres ventilatoires :
  - mode ventilatoire (VAC/AI/APRV/BIPAP)
  - FIO_2 (%)
  - volume courant (ml)
  - fréquence respiratoire (/min)
  - niveau de PEP externe (cm H2O)
  - niveau de PEP totale du système respiratoire (cm H2O) si mesurable
  - pression de crète du système respiratoire (cm H2O)
  - pression plateau du système respiratoire (cm H2O) si mesurable
  - Ti/Ttot (1/x)
  - débit inspiratoire (l/min)
- paramètres hémodynamiques
  - dose de noradrénaline (µg/kg/min ou mg/h au choix du centre)
  - dose d'adrénaline (µg/kg/min ou mg/h au choix du centre)
  - dose de dobutamine (µg/kg/min ou mg/h au choix du centre)
  - diurèse des dernières 24 heures
- paramètres neurologiques
  - score RASS
  - score de Glasgow
  - dose de midazolam (mg/H
  - dose de propofol (mg/H
  - dose de morphine (mg/H)
  - dose de fentanyl (µg/H)
  - dose de sufentanil (µg/H)
  - type et dose de curare (molécule, dose en mg/h)
- données clinico-biologiques supplémentaires permettant de calculer le score SOFA [41] le jour de l'inclusion (plaquettes, bilirubine, créatinine, diurèse des dernières 24 heures)
- gaz du sang (PaO2, pH, PaCO2, bicarbonates, SaO2)
- position du malade pendant les gaz du sang (décubitus dorsal/décubitus ventral)
- lactates
- traitements adjuvants parmi les suivants au cours des dernières 24 heures (monoxyde d'azote inhalé, décubitus ventral, manœuvres de recrutement, épuration extrarénale, ECMO, bicarbonates IV (dose en grammes/24H))
- paramètres échocardiographiques (uniquement à J1)

    - rapport des surfaces ventriculaire droite et gauche
    - présence d'une dyskinésie septale
- Evènement indésirable depuis les dernières 24 heures
    - épisode d'acidose respiratoire défini par un pH<7,15 avec paCO2 > 45 mm Hg
    - nouvelle pneumonie acquise sous ventilation mécanique avec initiation d'antibiothérapie
    - cœur pulmonaire aigu
    - barotraumatisme (pneumothorax, ou pneumomédiastin, ou emphysème sous cutané ou pneumatocèle de plus de 2 cm en imagerie)
    - autre EIG

Données relevées tous les jours entre J15 et J28

- épisode d'acidose respiratoire défini par un pH<7,15 avec paCO2 > 45 mm Hg
- nouvelle pneumonie acquise sous ventilation mécanique avec initiation d'antibiothérapie
- cœur pulmonaire aigu
- barotraumatisme (pneumothorax, ou pneumomédiastin, ou emphysème sous cutané ou pneumatocèle de plus de 2 cm en imagerie)
- autre EIG

Données relevées à la sortie de réanimation

- Date de sortie de réanimation
- Date d'extubation avec succès définie comme une extubation sans ré-intubation dans les 48 heures (ou l'arrêt de la ventilation invasive pendant plus de 48 heures chez les patients trachéotomisés)
- Date d'arrêt des curares en administration continue (définie comme l'absence de reprise de curare en perfusion continue pendant plus de 48h)

Données relevées J60 après l'inclusion

- Nombre de jours vivant sans ventilation mécanique depuis l'inclusion, par recherche de l'information dans le dossier médical du patient. Si le patient est transféré dans un autre hôpital avant sont extubation, sa date d'extubation avec succès sera recherchée par contact avec le médecin en charge.

Données relevées J90 après l'inclusion (±2 jours)

- Mortalité toute cause
- Date de sortie de l'hôpital censurée à J90

7.3.4 Visite de fin de la recherche

La fin de la recherche est définie sur l'un des critères suivants

- *J365*
- *Décès du patient*

Données relevées J365 après l'inclusion (±10 jours)

- Score de trouble cognitif T-MOCA récupéré par appel téléphonique
- Score de qualité de vie SF-36 récupéré par appel téléphonique
- score IES-R récupéré par appel téléphonique

Cet appel téléphonique sera réalisé par l'investigateur principal du centre associé ou une personne du centre désignée par l'investigateur.

7.3.5 Echantillons biologiques

Aucun échantillon biologique ne sera prélevé pour l'étude

7.3.6 Distinction soins et recherches

Procédures effectuées au cours de la recherche	Procédures effectuées dans le cadre du soin	Procédures ajoutées du fait de la recherche
Traitement	Réglages ventilatoires<br>Adaptation de la sédation<br>Curarisation éventuelle<br>Sevrage respiratoire	Ventilation à 4 ml/kg dans le bras expérimental<br>Ventilation à 6 ml/kg dans le bras contrôle
Consultations	Quotidienne de J0 à J14 en réanimation si le patient est ventilé<br>A la sortie de réanimation	Aucune
Echantillons biologiques	Gaz du sang, biologie standard quotidienne	Aucun
Imagerie	Radiographie pulmonaire à la demande<br>Scanner thoracique à la demande	Aucune
Autres (questionnaires…)	Aucun	Questionnaire téléphonique à J365

7.4 Règles d'arrêt temporaire ou définitif

- *Arrêt de participation d'une personne à la recherche :*

Les sujets pourront retirer leur consentement et demander à sortir de l'étude à n'importe quel moment et quelle qu'en soit la raison. En cas de sortie prématurée, l'investigateur doit en documenter les raisons de façon aussi complète que possible.

L'investigateur pourra interrompre temporairement ou définitivement la participation d'un sujet à l'étude pour toute raison qui servirait au mieux les intérêts du sujet en particulier en cas d'événements indésirables graves.

En cas de sujet perdu de vue, l'investigateur mettra tout en œuvre pour reprendre contact avec la personne recueillir si possible la raison de la perte de vue.

En cas de retrait de consentement, les données recueillies jusqu'à la date du retrait seront analysées

Dans l'hypothèse ou des patients aient été inclus à tort dans l'étude (c'est-à-dire ne présentant pas tous les critères d'inclusion ou présentant au moins un critère de non-inclusion), ils seront exclus de l'analyse et seront remplacés par une inclusion supplémentaire pour garantir le nombre de patients analysables prévu.

- *Arrêt d'une partie ou de la totalité de la recherche :*

L'étude peut être interrompue prématurément en cas de survenue d'événements indésirables inattendus, graves nécessitant une revue du profil de la stratégie. De même, des événements imprévus ou de nouvelles informations relatives à la méthode d'investigation, au vu desquels les objectifs de l'étude ne seront vraisemblablement pas atteints, peuvent amener le promoteur à interrompre prématurément l'étude.
Les Hospices Civils de Lyon se réservent le droit d'interrompre l'étude à tout moment, s'il s'avère que les objectifs d'inclusion ne sont pas atteints.
Le Comité de Surveillance Indépendant se réunira tous les 30 patients décédés. Les données de sécurité seront présentées et le CSI donnera son avis sur la poursuite de l'essai. En cas de différence significative de mortalité entre les 2 groupes en défaveur du groupe expérimental nécessitant des mesures urgentes du promoteur, le promoteur en informera les autorités par un fait nouveau et une mesure urgente de sécurité.
En cas d'arrêt prématuré de l'étude pour des raisons de sécurité, l'information sera transmise par le promoteur sans délai à l'ANSM et au CPP.

7.5 Collection d'échantillons biologiques

Non applicable

8 ÉVALUATION DE LA SÉCURITÉ

8.1 Définitions

Selon l'article R1123-46 du code de la santé publique

8.1.1 Evénement indésirable

Toute manifestation nocive survenant chez une personne qui se prête à une recherche impliquant la personne humaine que cette manifestation soit liée ou non à la recherche.

8.1.2 Evénement ou effet indésirable grave (EIG)

Tout événement ou effet indésirable :
- qui entraîne la **mort** ; ou
- qui **met en danger la vie de la personne** qui se prête à la recherche ; ou
- qui nécessite une **hospitalisation ou une prolongation d'hospitalisation** ; ou
- qui provoque une **incapacité ou un handicap importants ou durables** ; ou
- qui se traduit par une **anomalie ou une malformation congénitale** ; ou
- tout **autre événement médicalement important** ne répondant pas aux qualifications énumérées ci-dessus :
  - o mais pouvant être considéré comme **« potentiellement grave »** notamment certaines anomalies biologiques ;
  - o ou **événement médicalement pertinent selon le jugement de l'investigateur** ;
  - o ou encore **un événement nécessitant une intervention médicale** pour prévenir l'évolution vers un des états précités.
  - o Se référer à la section 8.2.3 Evénements Indésirables d'Intérêt Particulier

Par exemple, ces événements peuvent être un traitement intensif aux urgences hospitalières ou au domicile du participant à la recherche pour un bronchospasme allergique, une crise convulsive ou des troubles de la coagulation.

L'expression " mettre la vie en danger" est réservée à une menace vitale immédiate, au moment de l'événement indésirable, et ce, indépendamment des conséquences qu'aurait une thérapeutique correctrice ou palliative.

Certaines circonstances nécessitant une hospitalisation ne relèvent pas du critère de gravité.

8.1.3 Effet indésirable (EI)

Toute réaction nocive et non désirée due à la recherche impliquant une personne humaine dans sa globalité.

8.1.4 Effet indésirable inattendu

Effet indésirable dont la nature, la sévérité, la fréquence ou l'évolution ne concorde pas avec les informations de référence sur la sécurité mentionnés dans le protocole ou dans la brochure pour l'investigateur.

8.1.5 Fait nouveau

Toute nouvelle donnée pouvant conduire à une réévaluation du rapport des bénéfices et des risques de la recherche ou du produit objet de la recherche, à des modifications dans l'utilisation de ce produit, dans la conduite de la recherche, ou des documents relatifs à la recherche, ou à suspendre ou interrompre ou modifier le protocole de la recherche ou des recherches similaires.

8.2 *Responsabilités de l'investigateur*

8.2.1 *Modalités de détection et de recueil des événements indésirables*

Tous les événements indésirables doivent être recherchés, rapportés et enregistrés, traités et évalués de la première visite (inclusion J0) jusqu'à la fin de l'étude et jusqu'à leur résolution.

Tous les événements indésirables seront notés sur les formulaires de recueil des événements indésirables du cahier d'observation. Chaque événement indésirable observé sera consigné individuellement. L'intensité des événements indésirables sera déterminée de la façon suivante :

- **légère (grade 1)** : pas d'interférence sur l'activité au quotidien du patient ;
- **modérée (grade 2)** : interférence modérée sur l'activité quotidienne du patient mais encore acceptable ;
- **sévère (grade 3)** : interférence importante sur l'activité quotidienne du patient et inacceptable ;
- **menace du pronostic vital (grade 4)** ;
- **décès (grade 5)**.

Tous les évènements indésirables doivent être gradés. Tous les événements indésirables **d'intensité sévère, menace du pronostic vital et décès (grade supérieur ou égal à 3) sont considérés comme GRAVE et doivent être notifiés sans délai au promoteur**

Les événements suivant ne sont pas considérés comme des événements indésirables :

- Admission pour raisons sociale ou administrative ;
- Hospitalisation prédéfinie par le protocole ;
- Passage en hôpital de jour programmé dans le cadre du suivi de la pathologie étudiée ou d'une maladie intercurrente déjà connue à l'inclusion ;

8.2.2 *Notification des EIG*

L'investigateur évalue chaque événement indésirable au regard de sa gravité.
L'investigateur doit notifier au promoteur, sans délai et au plus tard dans les 24 heures à compter du jour où il en a connaissance, tous les événements indésirables graves survenus dans l'essai, à l'exception de ceux qui sont recensés dans le protocole comme ne nécessitant pas une notification sans délai.
Cette notification initiale fait l'objet d'un rapport écrit et doit être suivie par un ou des rapport(s) complémentaire(s) écrit(s) détaillé(s) **dans les 8 jours** suivant la première notification.
L'investigateur valide le formulaire de notification d'EIG et l'envoie par mail via l'eCRF à drci.eig-vigilance@chu-lyon.fr , dès lors qu'il a les 4 éléments minimum pour notifier un EIG :

- Un notificateur
- Un sujet
- La **procédure** expérimentale
- Un événement indésirable

L'investigateur doit documenter au mieux l'événement (grâce aux copies des résultats de laboratoires ou des comptes rendus d'examens ou d'hospitalisation renseignant l'événement grave, y compris les résultats négatifs pertinents, **sans omettre de rendre ces documents anonymes** et d'inscrire le numéro et le code du patient), **le diagnostic médical** et établir un **lien de causalité** entre l'événement indésirable grave et **et la (les) procédure(s) expérimentale(s).**

L'investigateur doit suivre le patient ayant présenté un EIG jusqu'à sa résolution, une stabilisation à un niveau jugé acceptable par l'investigateur ou le retour à l'état antérieur, même si le patient est sorti de l'essai et informer le promoteur en complétant l'eCRF, puis en envoyant par mail via eCRF à drci.eig-vigilance@chu-lyon.fr (remplir la partie FU avec nom, date et signature sur l'ECRF).

Si l'eCRF est indisponible, l'investigateur peut scanner le formulaire de notification d'EIG signé et daté et l'envoyer par mail à drci.eig-vigilance@chu-lyon.fr ou par fax au 04 72 11 51 90. Il doit préciser dans l'objet du mail **« VT4-COVID-Critère de gravité-N°centre-patient ».**

8.2.3 Evaluation de la causalité

L'investigateur doit évaluer le lien de causalité des événements indésirables avec la recherche. Le lien de causalité est binaire (raisonnablement relié / non relié).

8.2.4 Période de notification des EIG, restriction des EI/EIG et événements indésirables d'intérêt particulier

L'investigateur doit notifier sans délai au promoteur les évènements indésirables graves **dès *L'INCLUSION* DU PATIENT *(date de signature du 1er consentement)* et jusqu'à la fin de participation du patient**, et sans limite de durée pour les évènements indésirables graves reliés à la recherche.

Compte tenu de la réalisation de l'étude en réanimation sur des patients avec un taux de mortalité très élevé et de multiples complications attendues en réanimation, des restrictions sont mises en place.
Le tableau ci-dessous résume les événements qui ne seront pas collectés dans la section des événements indésirables du CRF, ni notifiés en EIG au promoteur avec le formulaire de notification d'EIG.

<u>Tableau récapitulatif des restrictions des EI/EIG:</u>

**Période de participation**	**<u>Restriction des événements dans le CRF</u> (pas de collecte dans le CRF, ni d'envoi de formulaire de notification d'EIG au promoteur)**	**<u>Restriction des notifications d'EIG au promoteur</u> (collecte uniquement dans le CRF, pas d'envoi de formulaire de notification d'EIG au promoteur)**	**<u>Evénements d'intérêt particulier : A notifier immédiatement au promoteur en EIG</u>**
**Séjour en réanimation à partir de l'inclusion**	- Insuffisance rénale aiguë de grade < 3 selon la classification KDIGO [42]<br>- Troubles métaboliques à l'exclusion de l'acidose respiratoire sévère définie par un pH<7,15 et une PaCO2 > 45 mm Hg<br>- Infections nosocomiales non fatales sauf bactériémies et pneumonies nosocomiales<br>- Chocs de tout type sauf ceux liés à un cœur pulmonaire aigu<br>- Aggravation de l'état respiratoire non fatale<br>- Neuromyopathie de réanimation	- Insuffisance rénale aiguë de grade 3 selon la classification KDIGO [42]<br>- Cœur pulmonaire aigu défini par une dilatation du ventricule droit et une dyskinésie septale en échocardiographie sans signe de gravité (surface VD/surface VG < 1). Cette complication est en effet attendue avec une fréquence de 20% environ [1] dans le bras contrôle, et n'est associé à une surmortalité que dans les formes sévères (surface VD/surface VG≥1)<br>- Thrombophlébite et embolie pulmonaire non fatales	**Tous les décès**<br><br>**Cœur pulmonaire aigu** défini par une dilatation du ventricule droit et une dyskinésie septale en échocardiographie avec signe de gravité **(surface VD/surface VG ≥ 1)**
**Sortie de réanimation et séjour en hospitalisation**	idem	idem	**Tous les décès**<br>**Tous les retours en réanimation**
**A partir de la sortie d'hospitalisation à la fin de participation à la recherche**	**Collecter dans le CRF uniquement les événements correspondant aux critères de jugement de l'étude.**		**Tous les décès**<br>**Tous les retours en réanimation**

8.3 *Responsabilités du promoteur*

8.3.1 *Déclaration aux autorités compétentes*

Le Promoteur déclarera à l'ANSM:

- o en cas de mise en jeu du pronostic vital ou de décès du sujet : toutes les suspicions d'effets indésirables graves inattendus sans délai à compter du jour où le promoteur en a connaissance et les informations complémentaires pertinentes devant être soumises sous forme de rapport de suivi dans un délai de 8 jours dès réception du suivi.
- o pour tous les autres effets indésirables graves inattendus : au plus tard dans un délai de 15 jours à compter du jour où le promoteur en a connaissance, et les informations complémentaires pertinentes devant être soumises sous forme d'un rapport de suivi dans un nouveau délai de 8 jours dès réception du suivi.

Selon l'article R1123-59 du CSP, le promoteur déclarera :
- à l'ANSM et au CPP les faits nouveaux et/ou mesures urgentes de sécurité sans délai à compter du jour où le promoteur en a connaissance, 8 jours dès connaissance d'une nouvelle information de sécurité pertinente pour les suivis.

POUR TOUS
Il établira un également rapport annuel de sécurité (RAS) qui sera transmis à l'ANSM et au CPP sous un délai de 60 jours après la date anniversaire de l'étude.

8.3.2 *Description des effets indésirables liés à la recherche (référence de sécurité pour l'évaluation du caractère attendu/inattendu par le promoteur)*

Les effets indésirables potentiellement liés à la recherche sont les suivants :

- Acidose mixte définie par un pH < 7.15 et une $PaCO_2$ > 45 mm Hg
- Cœur pulmonaire aigu défini par une dilatation du ventricule droit et une dyskinésie septale en échocardiographie, sachant que cette complication survient dans plus de 20% des SDRA traité par ventilation protectrice.

Description des événements indésirables reliés à la maladie étudiée :
Il n'est pas possible de décrire l'ensemble des effets indésirables liés à la maladie étudiée dans la mesure où les patients de réanimation ont de multiples complications au cours du séjour (a fortiori si le séjour est long).

La mortalité du SDRA compliquant une pneumonie à Covid-19 est extrêmement élevée et atteint 50 à 60%.
Par conséquent, les EIG d'évolution fatale seront considérés comme non reliés à la recherche. Néanmoins, si un déséquilibre significatif de mortalité est observé dans le groupe expérimental, le CSI sera consulté et le promoteur mettra en place des mesures urgentes de sécurité si nécessaire (ex : arrêt des inclusions). Le promoteur en informera l'ANSM et le CPP sans délai par un fait nouveau et une mesure urgente de sécurité.

8.4 *Comité de surveillance Indépendant*

Le Comité de Surveillance Indépendant est un comité consultatif chargé de donner au Promoteur d'un essai clinique, un avis sur la conduite de l'essai. A cet effet, il examine l'ensemble des problèmes pouvant apparaître dans l'essai, notamment scientifiques, éthiques et de tolérance, susceptibles de modifier le rapport bénéfice/risque. Suite à cet examen, il transmet par écrit ses recommandations au Promoteur. Ces recommandations peuvent concerner notamment la poursuite, la modification ou l'arrêt de l'essai.
Le promoteur reste décisionnaire des mesures à mettre en œuvre, suite aux recommandations du CSI.
Les modalités de fonctionnement de ce CSI sont décrites dans une charte signée par les membres du CSI en début de recherche.
Il est composé au moins de deux experts cliniciens et d'un méthodologiste/biostastisticien.
Le CSI se réunira tous les 30 décès.

9 Evaluation médico-économique

9.1 Principales caractéristiques de l'évaluation

La ventilation ultraprotectrice sans épuration extracorporelle doit permettre de réduire la mortalité et la durée de ventilation mécanique du SDRA compliquant une pneumopathie à Covid-19, en réduisant les effets délétères de la ventilation mécanique. En plus de l'amélioration attendue de la santé des patients, cette stratégie innovante pourrait entrainer une diminution des coûts de prise en charge imputable notamment de la diminution de la durée du séjour en réanimation. Aucune évaluation médico-économique n'a été menée à ce jour pour montrer l'intérêt de la ventilation ultraprotectrice par rapport à la ventilation protectrice chez les patients atteints de pneumonie à Covid-19, avec SDRA modérément sévère à sévère. Dans le contexte de la diffusion de l'utilisation de cette stratégie innovante en routine des soins en France, il est important d'en évaluer l'impact sur la prise en charge de ces patients. C'est pourquoi nous proposons de réaliser, en objectif secondaire, une évaluation médico-économique afin d'étudier l'efficience de la stratégie basée sur une ventilation ultraprotectrice.

9.1.1 Type d'étude

Nous faisons l'hypothèse que la stratégie innovante, basée sur la ventilation ultraprotectrice, en comparaison à la stratégie de prise en charge standard, basée sur la ventilation protectrice, permettra de diminuer la mortalité des patients et la durée de ventilation mécanique du SDRA.

Compte-tenu de ces hypothèses de travail, nous mènerons une étude de type coût-efficacité. La stratégie innovante sera comparée à la stratégie de prise en charge standard sur la double dimension des coûts et de l'efficacité. Le critère d'efficacité retenu est la mortalité. Ce critère de jugement terminal est conforme aux recommandations de la Haute Autorité de Santé [43] et pertinent au vu des hypothèses de recherche. Cependant il ne prend pas en compte la qualité de vie fortement impactée par la ventilation mécanique. L'administration d'un questionnaire EQ-5D à des patients sous ventilation dans un service de réanimation, et donc le recours à une étude coût-utilité, ne semblent pas pertinents. Afin de prendre en compte indirectement l'impact sur la qualité, nous mènerons également une étude coût-efficacité avec le nombre jours vivants sans ventilation mécanique comme critère d'efficacité.

9.1.2 Perspective et horizon temporel

La perspective retenue est celle de la collectivité conformément aux recommandations. Nous retenons un horizon temporel de 90 jours, permettant de prendre en compte à la fois l'impact sur les bénéfices (mortalité, nombres de jours vivant sans ventilation) mais également sur les consommations de soins (séjour en réanimation essentiellement).

9.2 Evaluation des coûts

La prise en charge étant essentiellement hospitalière, seuls les coûts directs hospitaliers seront pris en compte.

9.2.1 Recueil des données de coût

Les données de l'Etude Nationale de Coûts (ENC), nécessaires à la valorisation des coûts des séjours hospitaliers seront recueillies auprès des Départements d'Information Médicale (DIM) de chaque centre selon le circuit suivant :

- Le Service d'Evaluation Economique en Santé informera les DIM de la sollicitation à venir et contactera les investigateurs principaux des centres en précisant les éléments suivants : identifiant étude des patients, mois et année de naissance, dates d'entrée et de sortie d'hospitalisation (données disponibles dans l'eCRF).
- L'investigateur du centre identifiera ses patients et se mettra en relation avec son DIM qui effectuera l'extraction des données sur les séjours des patients correspondants.
- Ces données seront à nouveau pseudonymisées (seulement identifiant étude du patient) par le centre (investigateur ou DIM) avant d'être transmises de façon sécurisée au Service d'Evaluation Economique en Santé.

9.2.2 Identification, quantification et valorisation des coûts

Les hospitalisations seront dénombrées à partir des Résumés d'Unité Médicale des séjours des patients fournis par les Départements d'Information Médicale des établissements concernés selon la version des Groupes Homogènes de Malades en vigueur. Ces séjours hospitaliers seront valorisés au coût de production en utilisant les données de coûts de l'ENC.
Les coûts ne seront pas actualisés puisque l'horizon temporel ne dépasse pas un an.

9.3 Présentation et interprétation des résultats

Une fois que le coût moyen et l'efficacité moyenne par patient auront été calculés pour chacune des deux stratégies étudiées, les résultats seront représentés dans un plan coût-efficacité.
Ce graphique, qui représente en abscisse le différentiel de coût et en ordonnée le différentiel d'efficacité, permet de voir si une des deux stratégies est dominée.
Si la stratégie innovante est moins coûteuse et plus efficace que la stratégie de référence, elle sera considérée comme efficiente. A l'inverse, si elle est plus coûteuse et moins efficace, nous pourrons conclure à sa non-efficience.
Dans tous les autres cas de figure, il nous faudra mettre en relation les deux dimensions de coût et d'efficacité en estimant le ratio différentiel coût-efficacité (RDCE) :

$$\text{RDCE} = \frac{\text{Coût de la stratégie innovante - Coût de la stratégie de référence}}{\text{Efficacité de la stratégie innovante - Efficacité de la stratégie de référence}}$$

Si la stratégie innovante est moins coûteuse et moins efficace, le ratio fournira une information sur la baisse d'efficacité qu'il faut consentir pour diminuer les coûts.
Si la stratégie innovante est plus coûteuse et plus efficace, le ratio nous fournira une information sur le coût supplémentaire engendré par la stratégie innovante par rapport à la stratégie de référence par gain d'efficacité.
Dans cette étude, le RDCE pourra être interprété comme le coût supplémentaire de la stratégie basée sur l'utilisation de la ventilation ultraprotectrice par jour vivant, ou jour vivant sans ventilation mécanique, supplémentaire en comparaison à la stratégie basée une ventilation protectrice pour la prise en charge des patients atteints de pneumonie à Covid-19, avec SDRA modérément sévère à sévère.

10 ASPECTS STATISTIQUES

10.1 Nombre de sujets nécessaires

Le calcul du nombre de sujets nécessaire a été effectué en simulant la mortalité à 90 j et le nombre de jours sans ventilation à 60 jours chez les patients du groupe ventilation ultraprotectrice et chez les patients du groupe ventilation protectrice sous les hypothèses suivantes :

- Mortalité à 90 j attendue dans le groupe ventilation protectrice égale à 45 % et réduction attendue de 11% dans le groupe ventilation ultraprotectrice (mortalité à 90 j égale à 40%)
- Nombre de jours sans ventilation à 60 jours égal en moyenne à 28±16,5 j dans le groupe ventilation protectrice et à 34,5±13 j dans le groupe ventilation ultraprotectrice

La mortalité attendue chez les patients COVID + présentant un SDRA modérément sévère à sévère est plus élevée que celle des patients non COVID. Elle se situe entre 40 et 50%.
Le nombre de jours sans ventilation à 60 j attendu provient des résultats d'un essai randomisé comparant la ventilation ultraprotectrice avec extraction de CO_2 et la ventilation protectrice standard, dans le sous-groupe des patients avec un ratio $PaO_2/FiO_2 < 150$ mm Hg [8]. Pour être conservateur, nous avons choisi de réduire la différence observée entre les 2 groupes de 50% (nombre de jours sans ventilation à 60 j attendu dans le groupe ventilation ultraprotectrice égal à 34,5±13 j au lieu de 41±13 j).

Sur chaque jeu de données simulées, les comparaisons par paire ont permis de calculer le score correspondant au critère de résultat principal pour chaque patient. Les scores ont été ensuite comparés entre les 2 groupes par un test de Mann et Whitney.
Les simulations ont été répétées 1000 fois pour un nombre de patients par groupe fixé et la puissance a été calculée comme la proportion de test avec une valeur de p <0,05 en test bilatéral.
Les résultats des simulations ont montré que l'inclusion de 100 patients par groupe permettra de conclure à une différence entre les 2 groupes avec une puissance d'environ 81%.
Les simulations ont été réalisées en parallèle par 2 statisticiens avec le logiciel de SAS, version 9.4 (Copyright (c) 2002-2003 by SAS Institute Inc., Cary, NC, USA) et le logiciel R, version 3.6.1 (R Core Team (2012). Les résultats obtenus étaient similaires.

10.2 Population d'analyse

L'analyse du critère de résultat principal et des critères secondaires sera réalisée en intention de traiter.
La population en intention de traiter est définie comme l'ensemble des patients inclus dans l'étude selon le bras alloué lors de la randomisation, quels que soient les critères d'éligibilité et la quantité de traitement reçue, qu'ils soient évaluables ou non évaluables pour les critères de jugement. La description de la population à l'inclusion se fera en intention de traiter.
Une analyse secondaire en per protocole du critère de résultat principal et des critères secondaires sera également réalisée.
La population per protocole est définie comme la population ITT dont seront exclus les patients avec déviation majeure au protocole.
Les cas de déviation majeure seront revus avant le gel de base. Ils seront spécifiés dans le plan d'analyse statistique. Les patients seront considérés dans le groupe correspondant à la ventilation réellement administrée.

10.3 Méthode statistiques

10.3.1 Description des caractéristiques des patients

Les caractéristiques des patients seront décrites et comparées entre les 2 groupes pour vérifier l'efficacité de la randomisation.
Les données quantitatives seront décrites dans chaque groupe, à l'aide des statistiques descriptives suivantes : l'effectif, le nombre de valeurs manquantes, la moyenne, l'écart type, la médiane, le premier et le troisième quartile ainsi que le minimum et le maximum. Ces statistiques seront considérées comme les statistiques usuelles pour l'analyse de variables quantitatives. Les variables quantitatives pourront être catégorisées en utilisant un seuil issu de la littérature médicale.
Les variables qualitatives seront décrites dans chaque groupe, à l'aide des statistiques descriptives suivantes : les fréquences absolue et relative pour chaque niveau de la variable et les valeurs manquantes (les valeurs manquantes seront dénombrées mais ne seront pas incluses dans le dénominateur du calcul des fréquences). Ces statistiques seront considérées comme les statistiques usuelles pour l'analyse de variables qualitatives.
Les caractéristiques quantitatives seront comparées entre les 2 groupes par le test t de Student ou par le test non paramétrique de Mann et Whitney. Les caractéristiques qualitatives seront comparées entre les 2 groupes par le test du chi2 ou le test exact de Fisher.

10.3.2 Analyse du critère de résultat principal

Le critère de résultat principal correspond au score établi pour chaque patient à partir de la comparaison à tous les patients de l'autre groupe sur la mortalité à 90 j en priorité et sur le nombre de jours sans ventilation à 60 jours en second critère.
La distribution du score dans chacun des groupes sera décrite par la moyenne et l'écart type ainsi que par les quartiles et les valeurs minimum et maximum.
Les scores seront comparés entre les 2 groupes par le test non paramétrique de Mann et Whitney.
Le bénéfice de la ventilation ultraprotectrice sera quantifié par la probabilité qu'un patient ayant reçu la ventilation ultraprotectrice ait un résultat favorable par rapport à un patient ayant reçu la ventilation protectrice. Cette probabilité sera estimée par la proportion de paires pour lesquelles le résultat sera favorable

pour le patient ayant reçu la ventilation ultraprotectrice [44]. Cette estimation sera donnée avec un intervalle de confiance à 95%.

10.3.3 Analyse des critères de résultat secondaires
Les critères de résultat qualitatifs tels que la mortalité à 90 j seront décrits dans les 2 groupes par la proportion d'événements et comparés par le test du chi2 ou le test exact de Fisher.
Les critères de résultat quantitatifs tels que le nombre jours sans ventilation à 60 j seront décrits dans les 2 groupes par la moyenne et l'écart type ou les quartiles selon la forme de la distribution. Ils seront comparés entre les 2 groupes par le test t de Student ou le test non paramétrique de Mann et Whitney.

Toutes les analyses seront réalisées avec le logiciel de statistique SAS, version 9.4 (Copyright (c) 2002-2003 by SAS Institute Inc., Cary, NC, USA). Les comparaisons seront définies statistiquement significatives pour une valeur de $p<0.05$ en bilatéral.

10.3.4 Analyse des données médico-économiques
Dans la mesure où l'étude médico-économique sera menée en parallèle de l'étude clinique, les effets et les coûts seront obtenus à partir de données échantillonnées provenant des mêmes patients et les variables de coûts ne seront pas analysées comme des estimations ponctuelles mais de façon stochastique, à l'aide des méthodes statistiques usuelles. Les données de coûts seront décrites en termes de moyennes, de variances et d'intervalles de confiance. Il conviendra de vérifier la normalité ou la non-normalité des distributions de coût par le test de Kolmogorov-Smirnov. Si les données de coût s'avèrent être non-normales, il s'agira de recourir aux méthodes non paramétriques standards (tel que le test U de Mann-Whitney), aux méthodes consistant à rapprocher de la normalité la distribution des coûts ou à la méthode du bootstrap.
Dans le cadre d'une analyse stochastique, l'incertitude est une incertitude d'échantillonnage. Dans ce contexte, il conviendra d'utiliser toute l'information contenue dans les données individuelles et de recourir aux intervalles de confiance du ratio coût-efficacité pour prendre en compte ce type d'incertitude. On privilégiera la méthode paramétrique fondée sur le théorème de Fieller et l'approche non paramétrique par le bootstrap qui produisent de meilleurs résultats en termes de probabilité de couverture.
Pour étudier la robustesse des résultats de l'analyse coût-efficacité, une analyse de sensibilité déterministe sera menée en modifiant certains paramètres de l'étude, permettant de mesurer l'impact sur le résultat final de la variation de ces paramètres, que ce soit les coûts ou l'efficacité. Un diagramme de Tornado sera utilisé pour visualiser l'influence des variables sur les résultats en fonction des incertitudes sur les valeurs utilisées. En complémente de l'analyse de sensibilité déterministe, une analyse de sensibilité probabiliste sera également menée.

10.4 *Analyses intermédiaires*
Aucune analyse intermédiaire n'est prévue.

10.5 *Méthode de prise en compte des données manquantes*
La fréquence des données manquantes et leurs causes seront comparées par groupe de traitement.
Aucune méthode de remplacement des données n'est prévue a priori.

10.6 *Gestion des modifications apportées au plan d'analyse*
Un plan d'analyse statistique détaillé sera rédigé avant le gel de la base des données. Il tiendra compte de toute modification du protocole ou de tout événement inattendu survenu au cours de l'étude et ayant un impact sur les analyses présentées ci-dessus. Les analyses planifiées pourront être complétées en cohérence avec les objectifs de l'étude.
Toute modification apportée par la suite au plan d'analyse statistique devra être justifiée et donnera lieu à une nouvelle version du document. Ces déviations au plan d'analyse seront reportées dans le rapport final de l'étude. L'ensemble des documents sera conservé dans le dossier de l'étude.

10.7 *Responsable des analyses*
Dr Muriel Rabilloud
Service de Biostatistique et Bioinformatique des HCL
Pôle de Santé Publique

11 SURVEILLANCE DE LA RECHERCHE

Le comité de pilotage se réunira tous les mois, fera le point des inclusions, et diffusera à l'ensemble des investigateurs un compte-rendu de réunion.
Le comité de surveillance indépendant analysera tous les incidents graves et le déroulement général de l'étude. Il se réunira au moins tous les 6 mois. Il rendra son rapport au comité de pilotage et au promoteur.

12 DROITS D'ACCES AUX DONNEES ET DOCUMENTS SOURCES

12.1 Accès aux données

Conformément aux BPC :

- le promoteur est chargé d'obtenir l'accord de l'ensemble des parties impliquées dans la recherche afin de garantir l'accès direct à tous les lieux de déroulement de la recherche, aux données sources, aux documents sources et aux rapports dans un but de contrôle de qualité et d'audit par le promoteur ;

- les investigateurs mettront à disposition des personnes chargées du suivi, du contrôle de qualité ou de l'audit de la recherche impliquant la personne humaine, les documents et données individuelles strictement nécessaires à ce contrôle, conformément aux dispositions législatives et réglementaires en vigueur (articles L.1121-3 et R.5121-13 du code de la santé publique).

12.2 Documents sources

Les documents sources sont définis comme tout document ou objet original permettant de prouver l'existence ou l'exactitude d'une donnée ou d'un fait enregistrés au cours de l'étude clinique. Ils seront conservés pendant 25 ans par l'investigateur ou par l'hôpital s'il s'agit d'un dossier médical hospitalier.
Les documents sources de cette étude sont les suivants

- Dossier médical
- Feuille de surveillance de réanimation
- Examens biologiques réalisés en réanimation

12.3 Confidentialité des données

Conformément aux dispositions concernant la confidentialité des données auxquelles ont accès les personnes chargées du contrôle de qualité d'une recherche impliquant le personne humaine (article L.1121-3 du code de la santé publique), conformément aux dispositions relatives à la confidentialité des informations concernant notamment les essais, les personnes qui s'y prêtent et les résultats obtenus (article R. 5121-13 du code de la santé publique), les personnes ayant un accès direct aux données prendront toutes les précautions nécessaires en vue d'assurer la confidentialité des informations relatives aux essais, aux personnes qui s'y prêtent et notamment en ce qui concerne leur identité ainsi qu'aux résultats obtenus.

Ces personnes, au même titre que les investigateurs eux-mêmes, sont soumises au secret professionnel (selon les conditions définies par les articles 226-13 et 226-14 du code pénal).
Pendant la recherche impliquant la personne humaine ou à son issue, les données recueillies sur les personnes qui s'y prêtent et transmises au promoteur par les investigateurs (ou tous autres intervenants spécialisés) seront codées.

Elles ne doivent en aucun cas faire apparaître en clair les noms des personnes concernées ni leur adresse.

Modalités de codage des sujets :
Seules la première lettre du nom du sujet et la première lettre de son prénom seront enregistrées, accompagnées d'un numéro codé propre à l'étude indiquant l'ordre d'inclusion des sujets.

Le promoteur s'assurera que chaque personne qui se prête à la recherche a donné son accord par écrit pour l'accès aux données individuelles la concernant et strictement nécessaires au contrôle de qualité de la recherche.

13 CONTROLE ET ASSURANCE DE LA QUALITE

Un Attaché de Recherche Clinique (ARC) mandaté par le promoteur s'assurera de la bonne réalisation de l'étude, du recueil des données générées par écrit, de leur documentation, enregistrement et rapport, en accord avec les Procédures Opératoires Standards mises en application au sein de la DRCI des Hospices Civils de Lyon et conformément aux Bonnes Pratiques Cliniques ainsi qu'aux dispositions législatives et réglementaires en vigueur.

L'investigateur et les membres de son équipe acceptent de se rendre disponibles lors des visites de Contrôle de Qualité effectuées à intervalles réguliers par l'Attaché de Recherche Clinique. Lors de ces visites, les éléments suivant pourront être revus en fonction du niveau de monitoring adapté à l'étude et déterminé conformément aux POS du Promoteur :
Etude du 1°, selon le plan de monitoring

- consentement éclairé
- respect du protocole de l'étude et des procédures qui y sont définies
- qualité des données recueillies dans le cahier d'observation : exactitude, données manquantes, cohérence des données avec les documents sources
- gestion des traitements expérimentaux
- déclaration des événements indésirables graves.

Toute visite fera l'objet d'un rapport de monitoring par compte-rendu écrit adressé à l'investigateur du centre visité et à la structure de coordination de la recherche.

D'autre part, Les investigateurs s'engagent à accepter les audits d'assurance qualité effectués par des personnes mandatées par le promoteur ainsi que les inspections effectuées par les Autorités Compétentes. Toutes les données, tous les documents et rapports peuvent faire l'objet d'audits et d'inspections réglementaires sans que puisse être opposé le secret médical.

14 CONSIDERATIONS ETHIQUES

14.1 Autorités compétentes

Le protocole, la notice d'information et le formulaire de consentement de l'étude seront soumis pour avis au Comité de Protection des Personnes **Ile de France VII.**
La notification de l'avis favorable du CPP sera transmise au promoteur de l'étude et à l'ANSM. Une demande d'autorisation d'étude sera également adressée par le promoteur à l'ANSM.
Le promoteur s'engage à ce que le démarrage de l'étude ne se fasse qu'après obtention de l'avis favorable du CPP et de l'autorisation d'étude de l'ANSM.

14.2 Modifications substantielles

En cas de modification substantielle apportée au protocole par l'investigateur, elle sera approuvée par le promoteur. Ce dernier devra obtenir préalablement à sa mise en œuvre un avis favorable du CPP et une autorisation de l'ANSM dans le cadre de leurs compétences respectives. Un nouveau consentement des personnes participant à la recherche sera recueilli si nécessaire.

14.3 *Information du patient et formulaire de consentement écrit*

Dans la mesure où les patients éligibles seront très certainement tous sous sédation et curarisation (prise en charge habituelle du SDRA modérément sévère à sévère), aucun ne sera susceptible de pouvoir donner son consentement. Si les proches sont présents (cas peu probable en raison du confinement), ils seront informés de façon complète et loyale, en des termes compréhensibles, des objectifs et des contraintes de l'étude, des risques éventuels encourus, des mesures de surveillance et de sécurité nécessaires, de leurs droits de refuser de participer à l'étude ou de la possibilité de se rétracter à tout moment. Toutes ces informations figurent sur un formulaire d'information et de consentement qui sera remis au proche du patient. Le consentement libre, éclaire et écrit du proche du patient sera recueilli par l'investigateur, ou un médecin qui le représente avant l'inclusion définitive dans l'étude. Une copie du formulaire d'information et de consentement signé par les deux parties sera remise au proche du patient, l'investigateur en conservera l'original.

Si le représentant du patient n'est pas présent lors du screening (cas le plus probable en raison du confinement), l'inclusion d'un patient éligible pourra être réalisée selon une procédure d'urgence. L'investigateur remplit alors un formulaire confirmant l'absence de présence des proches du patient sur le site. L'information des proches et le recueil de leur consentement devra être réalisée dès que possible. En cas de refus de consentement par ses proches, le patient inclus selon la procédure d'urgence sera exclu de l'étude. Toutefois, les patients inclus selon la procédure d'urgence, sans proche identifiable, et qui décèderaient sans avoir repris un état de conscience normale, resteront inclus dans l'étude.

Une fois que le patient sera en état de donner son consentement, une notice d'information et un formulaire de confirmation de participation lui seront fournis. Le formulaire de confirmation de participation sera signé par le patient et une copie lui sera fournie. L'investigateur en conservera l'original.

14.4 *Déclaration de conformité*

Le promoteur et l'investigateur s'engagent à ce que cette recherche soit conduite :
- conformément au protocole,
- conformément aux bonnes pratiques cliniques françaises et internationales actuellement en vigueur,
- conformément aux dispositions législatives et réglementaires actuellement en vigueur en France et au niveau international.

14.5 *Période d'exclusion*

Le patient peut participer à une autre recherche sous réserve :
- qu'elle ne soit pas de catégorie1,
- ET qu'elle ne partage pas le même critère de jugement principal que la présente étude
- ET qu'elle n'interfère pas avec la stratégie ventilatoire testée (jugement de l'investigateur). Il s'agirait d'études testant des paramètres ventilatoires empêchant d'appliquer la ventilation ultraprotectrice ou la ventilation protectrice.

En conséquence, une période d'exclusion de 90 jours suivant l'inclusion dans la présente étude est prévue.

14.6 *Indemnisation des sujets et inscription au fichier national des personnes se prêtant à une recherche interventionnelle sur la personne humaine du 1°*

Il n'est pas prévu d'indemnisation pour les patients traités.

15 GESTION ET CONSERVATION DES DONNEES

15.1 Cahier d'observation

Le cahier d'observation ne comportera que les données nécessaires à l'analyse en vue de publication. Les autres données relatives au patient et nécessaires à son suivi en dehors de l'étude, seront colligées dans son dossier médical. Ce cahier d'observation se présentera sous forme électronique.

Les données de l'étude seront recueillies dans un cahier d'observation électronique (eCRF). Cet eCRF, spécifique à l'étude, sera développé par un datamanager des Hospices Civils de Lyon sur le logiciel Ennov Clinical® 7.5.720. Ce logiciel respecte les recommandations de la FDA sur les systèmes informatisés pour la gestion des essais cliniques (Guidance for Computerized Systems Used in Clinical Trials) ainsi que les recommandations FDA sur la signature électronique (21CFR part 11).
Le CRF n'inclura que les données nécessaires à la réalisation du protocole et à la publication scientifique. Les autres données des patients nécessaires à leur suivi en dehors de cette étude seront collectées dans le dossier médical du patient.
Les données de l'étude seront informatisées de façon codée, conformément à la loi informatique et liberté. Les sujets/patients de l'étude seront identifiés par leur numéro unique d'inclusion dans l'étude et par la première lettre de leur nom et de leur prénom. Une liste d'identification des participants à la recherche sera conservée dans le classeur investigateur.
Les données doivent être complétées, au fur et à mesure de leur collecte, par les personnes autorisées (investigateur et personnes apparaissant sur la délégation de tâches) et disposant de leurs propres identifiants, conformément à la loi informatique et liberté. Le remplissage du cahier d'observation via internet permet au centre de coordination et au promoteur de l'étude de visualiser rapidement et à distance les données. Lors de la saisie, les données sont immédiatement vérifiées grâce à des contrôles de cohérence. La personne en charge du remplissage doit valider et justifier toute modification de valeur dans le CRF. Les entrées et modifications font l'objet d'un audit trail.
L'investigateur est responsable de l'exactitude, de la qualité et de la pertinence de toutes les données saisies. A ce titre, chaque page du CRF patient doit être datée et signée électroniquement par l'investigateur, signifiant ainsi son accord et sa responsabilité vis-à-vis des données collectées.

15.2 Gestion des données

Les données des patients nécessaires à l'étude seront recueillies dans un cahier d'observation électronique (e-CRF). Ce e-CRF développé via le logiciel Clinsight® sera spécifique à l'étude.
Le code patient (cf le paragraphe « confidentialité des données ») sera la seule information sur le e-CRF, qui permettra de rattacher les données au patient.
L'e-CRF ne comportera que les données nécessaires à l'analyse de l'efficacité et de la sécurité des patients, en vue de publication (informations requises par le protocole). Les autres données relatives au patient et nécessaires à son suivi en dehors de l'étude, seront colligées dans son dossier médical. Les données devront être recueillies au fur et à mesure qu'elles sont obtenues. Une explication devra être apportée pour chaque donnée manquante.
En fin d'étude, une impression papier sera demandée, authentifiée (datée et signée) par l'investigateur. Une copie du document authentifié à destination du promoteur devra être archivée par l'investigateur.

15.3 CNIL

Cette étude entre dans le cadre de la « Méthodologie de Référence » (MR-001) en application des dispositions de l'article 54 alinéa 5 de la loi n°78-17 du 6 janvier 1978 modifiée relative à l'informatique, aux fichiers et aux libertés. Ce changement a été homologué par décision du 5 janvier 2006 et modifiée le 21 juillet 2016. Les Hospices Civils de Lyon, promoteur de l'étude, ont signé un engagement de conformité à cette « Méthodologie de Référence ».

15.4 *Archivage*

Les documents suivants seront archivés par le nom de l'étude sous la responsabilité de l'investigateur coordonnateur et des investigateurs associés au niveau de chaque centre pendant 25 ans :
- Protocole et annexes, amendements éventuels,
- Formulaires d'information et consentements originaux signés
- Données individuelles (copies authentifiées de données brutes)
- Documents de suivi et courriers relatifs à la recherche

Le Promoteur est également responsable d'organiser la conservation des analyses statistiques et du rapport final de l'étude pendant la durée réglementaire d'archivage.
Aucun déplacement ou destruction ne pourra être effectué sans l'accord du promoteur. Au terme des 25 ans, le promoteur sera consulté pour destruction. Toutes les données, tous les documents et rapports pourront faire l'objet d'audit ou d'inspection.

16 FINANCEMENT ET ASSURANCE

16.1 *Budget de l'étude*

249 Keuros
Les frais liés à cette recherche sont les suivants :
- Intervention propre à l'étude
- Examens relatifs au protocole
- Recrutement de personnel ;
- Contrôle qualité par un ARC mandaté par le promoteur ;
- Chef de projet - montage de l'étude ;
- Assurance de la recherche ;
- Pharmacovigilance de l'essai ;
- Gestion des données ;
- Frais divers.

16.2 *Assurance*

Le promoteur a souscrit pour toute la durée de l'étude une assurance garantissant sa propre responsabilité civile ainsi que celle de tout médecin impliqué dans la réalisation de l'étude. Il assurera également l'indemnisation intégrale des conséquences dommageables à la recherche pour la personne qui s'y prête et ses ayants droit, sauf preuve à sa charge que le dommage n'est pas imputable à sa faute ou à celle de tout intervenant, sans que puisse être opposé le fait d'un tiers ou le retrait volontaire de la personne qui avait initialement consenti à se prêter à la recherche.
Le contrat d'assurance a été souscrit avant le démarrage de l'étude auprès de la Société Hospitalière d'Assurance Mutuelle, 18 rue Edouard Rochet, 69008 Lyon, sous le numéro 159077.

17 REGLES RELATIVES A LA PUBLICATION

Les communications et rapports scientifiques correspondant à cette étude seront réalisés sous la responsabilité de l'investigateur principal de l'étude avec l'accord des investigateurs associés. Les coauteurs du rapport et des publications seront les investigateurs et les cliniciens impliqués, au prorata de leur contribution à l'étude, ainsi que le biostatisticien et les chercheurs associés.
Les règles de publications suivront les recommandations internationales (N Engl J Med, 1997; 336 :309-315).
L'étude sera enregistrée sur un registre des essais cliniques en libre accès (clinicaltrials.gov) avant l'inclusion du 1er patient.

18 REFERENCES BIBLIOGRAPHIQUES

1. Mekontso Dessap A, Boissier F, Charron C, et al (2016) Acute cor pulmonale during protective ventilation for acute respiratory distress syndrome: prevalence, predictors, and clinical impact. Intensive Care Med 42:862–870. https://doi.org/10.1007/s00134-015-4141-2

2. ARDS Definition Task Force, Ranieri VM, Rubenfeld GD, et al (2012) Acute respiratory distress syndrome: the Berlin Definition. JAMA 307:2526–2533. https://doi.org/10.1001/jama.2012.5669

3. Wu C, Chen X, Cai Y, et al (2020) Risk Factors Associated With Acute Respiratory Distress Syndrome and Death in Patients With Coronavirus Disease 2019 Pneumonia in Wuhan, China. JAMA Intern Med. https://doi.org/10.1001/jamainternmed.2020.0994

4. Arentz M, Yim E, Klaff L, et al (2020) Characteristics and Outcomes of 21 Critically Ill Patients With COVID-19 in Washington State. JAMA. https://doi.org/10.1001/jama.2020.4326

5. Bitker L, Mezidi M, Dhelft F, et al (submitted) Characteristics and outcomes of critically ill patients with CoViD-19: report from a tertiary French ICU

6. (2000) Ventilation with lower tidal volumes as compared with traditional tidal volumes for acute lung injury and the acute respiratory distress syndrome. The Acute Respiratory Distress Syndrome Network. N Engl J Med 342:1301–1308. https://doi.org/10.1056/NEJM200005043421801

7. Terragni PP, Rosboch G, Tealdi A, et al (2007) Tidal hyperinflation during low tidal volume ventilation in acute respiratory distress syndrome. Am J Respir Crit Care Med 175:160–6

8. Needham DM, Yang T, Dinglas VD, et al (2015) Timing of low tidal volume ventilation and intensive care unit mortality in acute respiratory distress syndrome. A prospective cohort study. Am J Respir Crit Care Med 191:177–185. https://doi.org/10.1164/rccm.201409-1598OC

9. Terragni PP, Del Sorbo L, Mascia L, et al (2009) Tidal Volume Lower than 6 ml/kg Enhances Lung Protection: Role of Extracorporeal Carbon Dioxide Removal. Anesthesiology 111:826–835. https://doi.org/10.1097/ALN.0b013e3181b764d2

10. Bein T, Weber-Carstens S, Goldmann A, et al (2013) Lower tidal volume strategy (≈3 ml/kg) combined with extracorporeal CO2 removal versus “conventional” protective ventilation (6 ml/kg) in severe ARDS : The prospective randomized Xtravent-study. Intensive Care Med 39:847–856. https://doi.org/10.1007/s00134-012-2787-6

11. Richard JC, Marque S, Gros A, et al (2019) Feasibility and safety of ultra-low tidal volume ventilation without extracorporeal circulation in moderately severe and severe ARDS patients. Intensive Care Med. https://doi.org/10.1007/s00134-019-05776-x

12. Beitler JR, Sarge T, Banner-Goodspeed VM, et al (2019) Effect of Titrating Positive End-Expiratory Pressure (PEEP) With an Esophageal Pressure-Guided Strategy vs an Empirical High PEEP-Fio2 Strategy on Death and Days Free From Mechanical Ventilation Among Patients With Acute Respiratory Distress Syndrome: A Randomized Clinical Trial. JAMA 321:846–857. https://doi.org/10.1001/jama.2019.0555

13. Yehya N, Harhay MO, Curley MAQ, et al (2019) Reappraisal of Ventilator-Free Days in Critical Care Research. Am J Respir Crit Care Med 200:828–836. https://doi.org/10.1164/rccm.201810-2050CP

14. Puntillo KA, Max A, Timsit J-F, et al (2014) Determinants of procedural pain intensity in the intensive care unit. The europain® study. Am J Respir Crit Care Med 189:39–47. https://doi.org/10.1164/rccm.201306-1174OC

15. Richard JC, Marque S, Gros A, et al (2019) Feasibility and safety of ultra-low tidal volume ventilation without extracorporeal circulation in moderately severe and severe ARDS patients. Intensive Care Med 45:1590–1598. https://doi.org/10.1007/s00134-019-05776-x

16. Guerin C, Reignier J, Richard J-C, et al (2013) Prone positioning in severe acute respiratory distress syndrome. N Engl J Med 368:2159–2168. https://doi.org/10.1056/NEJMoa1214103

17. Papazian L, Forel JM, Gacouin A, et al (2010) Neuromuscular blockers in early acute respiratory distress syndrome. N Engl J Med 363:1107–16. https://doi.org/10.1056/NEJMoa1005372

18. Retamal J, Libuy J, Jiménez M, et al (2013) Preliminary study of ventilation with 4 ml/kg tidal volume in acute respiratory distress syndrome: feasibility and effects on cyclic recruitment - derecruitment and hyperinflation. Crit Care 17:R16. https://doi.org/10.1186/cc12487

19. Vieillard-Baron A, Schmitt JM, Augarde R, et al (2001) Acute cor pulmonale in acute respiratory distress syndrome submitted to protective ventilation: incidence, clinical implications, and prognosis. Crit Care Med 29:1551–5

20. Osman D, Monnet X, Castelain V, et al (2009) Incidence and prognostic value of right ventricular failure in acute respiratory distress syndrome. Intensive Care Med 35:69–76. https://doi.org/10.1007/s00134-008-1307-1

21. Boissier F, Katsahian S, Razazi K, et al (2013) Prevalence and prognosis of cor pulmonale during protective ventilation for acute respiratory distress syndrome. Intensive Care Med 39:1725–1733. https://doi.org/10.1007/s00134-013-2941-9

22. Lhéritier G, Legras A, Caille A, et al (2013) Prevalence and prognostic value of acute cor pulmonale and patent foramen ovale in ventilated patients with early acute respiratory distress syndrome: a multicenter study. Intensive Care Med 39:1734–1742. https://doi.org/10.1007/s00134-013-3017-6

23. Hotchkiss JR, Blanch L, Murias G, et al (2000) Effects of decreased respiratory frequency on ventilator-induced lung injury. Am J Respir Crit Care Med 161:463–468. https://doi.org/10.1164/ajrccm.161.2.9811008

24. Retamal J, Borges JB, Bruhn A, et al (2015) High respiratory rate is associated with early reduction of lung edema clearance in an experimental model of ARDS. Acta Anaesthesiol Scand. https://doi.org/10.1111/aas.12596

25. Rich PB, Reickert CA, Sawada S, et al (2000) Effect of rate and inspiratory flow on ventilator-induced lung injury. J Trauma 49:903–911

26. Rich PB, Douillet CD, Hurd H, Boucher RC (2003) Effect of ventilatory rate on airway cytokine levels and lung injury. J Surg Res 113:139–145

27. Vaporidi K, Voloudakis G, Priniannakis G, et al (2008) Effects of respiratory rate on ventilator-induced lung injury at a constant PaCO2 in a mouse model of normal lung. Crit Care Med 36:1277–1283. https://doi.org/10.1097/CCM.0b013e318169f30e

28. Brun-Buisson C, Minelli C, Bertolini G, et al (2004) Epidemiology and outcome of acute lung injury in European intensive care units. Results from the ALIVE study. Intensive Care Med 30:51–61

29. Sakr Y, Vincent JL, Reinhart K, et al (2005) High tidal volume and positive fluid balance are associated with worse outcome in acute lung injury. Chest 128:3098–3108. https://doi.org/10.1378/chest.128.5.3098

30. Hough CL, Kallet RH, Ranieri VM, et al (2005) Intrinsic positive end-expiratory pressure in Acute Respiratory Distress Syndrome (ARDS) Network subjects. Crit Care Med 33:527–532

31. Buyse M (2010) Generalized pairwise comparisons of prioritized outcomes in the two-sample problem. Stat Med 29:3245–3257. https://doi.org/10.1002/sim.3923

32. Kahn JM, Andersson L, Karir V, et al (2005) Low tidal volume ventilation does not increase sedation use in patients with acute lung injury. Crit Care Med 33:766–71

33. Cheng IW, Eisner MD, Thompson BT, et al (2005) Acute effects of tidal volume strategy on hemodynamics, fluid balance, and sedation in acute lung injury. Crit Care Med 33:63–70; discussion 239-240

34. Tran Kiem C, Bosetti P, Salje H, et al (2020) Evolution attendue du nombre de lits nécessaires en service de réanimation. Groupe de modélisation de l'épidémie COVID

35. Ferguson N, Laydon D, Nedjati-Gilani G, et al (2020) Impact of non-pharmaceutical interventions (NPIs) to reduce COVID19 mortality and healthcare demand

36. Richard JC, Girault C, Leteurtre S, et al (2005) Ventilatory management of acute respiratory distress syndrome (ARDS) in adult patients and children (excluding neonates). Réanimation 14:323–32

37. Papazian L, Aubron C, Brochard L, et al (2019) Formal guidelines: management of acute respiratory distress syndrome. Ann Intensive Care 9:69. https://doi.org/10.1186/s13613-019-0540-9

38. Sessler CN, Gosnell MS, Grap MJ, et al (2002) The Richmond Agitation-Sedation Scale: validity and reliability in adult intensive care unit patients. Am J Respir Crit Care Med 166:1338–1344. https://doi.org/10.1164/rccm.2107138

39. Le Gall JR, Lemeshow S, Saulnier F (1993) A new Simplified Acute Physiology Score (SAPS II) based on a European/North American multicenter study. JAMA 270:2957–2963. https://doi.org/10.1001/jama.1993.03510240069035

40. McCabe WR, Jackson GG (1962) Gram-Negative Bacteremia: I. Etiology and Ecology. Arch Intern Med 110:847–855. https://doi.org/10.1001/archinte.1962.03620240029006

41. Vincent JL, Moreno R, Takala J, et al (1996) The SOFA (Sepsis-related Organ Failure Assessment) score to describe organ dysfunction/failure. On behalf of the Working Group on Sepsis-Related Problems of the European Society of Intensive Care Medicine. Intensive Care Med 22:707–710

42. Khwaja A (2012) KDIGO clinical practice guidelines for acute kidney injury. Nephron Clin Pract 120:c179-184. https://doi.org/10.1159/000339789

43. Choix méthodologiques pour l'évaluation économique à la HAS. In: Haute Autorité de Santé. https://www.has-sante.fr/jcms/r_1499251/fr/choix-methodologiques-pour-l-evaluation-economique-a-la-has. Accessed 31 Mar 2020

44. Acion L, Peterson JJ, Temple S, Arndt S (2006) Probabilistic index: an intuitive non-parametric approach to measuring the size of treatment effects. Stat Med 25:591–602. https://doi.org/10.1002/sim.2256
