# Supplementary material for: Open-label randomized controlled trial of ultra-low tidal ventilation without extracorporeal circulation in patients with COVID-19 pneumonia and moderate to severe ARDS: study protocol for the VT4COVID trial
Source: Trials. 2021 Oct 11;22:692. doi: 10.1186/s13063-021-05665-z (PMC8503716; doi:10.1186/s13063-021-05665-z)
Supplement: Supplementary file 3 — Additional file 3. WHO Trial Registration Dataset. [file 13063_2021_5665_MOESM3_ESM.docx]

| 1. **Primary Registry and Trial Identifying Number** | **ClinicalTrials.gov:**  **NCT04349618** |
| --- | --- |
| 1. **Date of Registration in Primary Registry** | **April 16, 2020.** |
| 1. **Secondary Identifying Numbers** |  |
| - - Identifiers assigned by the sponsor | **Hospices Civils de Lyon: 69HCL20_0322** |
| - - Identifiers issued by ethics committees | **IDRCB : 2020-A00869-30** |
| 1. **Source(s) of Monetary or Material Support** Major source(s) of monetary or material support for the trial (e.g. funding agency, foundation, company, institution). | **French Ministry of Health (PHRCI 2020) and Hospices Civils de Lyon** |
| 1. **Primary Sponsor** | **Hospices Civils de Lyon** |
| 1. **Secondary Sponsor(s)** | **None** |
| 1. **Contact for Public Queries** | **Valérie PLATTNER**  **E-mail:** [**valerie.plattner@chu-lyon.fr**](mailto:valerie.plattner@chu-lyon.fr)  **Tel.: 04 72 40 68 40**  **Address : Direction de la Recherche Clinique – Hospices Civils de Lyon. 3 quai des Célestins 69229 Lyon cedex 02 , FRANCE** |
| 1. **Contact for Scientific Queries** | **Principal Investigator**  **Dr Hodane YONIS**  **E-mail:** [**hodane.yonis@chu-lyon.fr**](mailto:hodane.yonis@chu-lyon.fr)  **Tel.: 04 72 07 17 62**  **Address : Service de Médecine Intensive Réanimation.103 grande rue de la Croix-Rousse. 69004 Lyon, France**  **Scientific contact :**  **Pr Jean-Christophe RICHARD**  **E-mail:** [**j-christophe.richard@chu-lyon.fr**](mailto:j-christophe.richard@chu-lyon.fr)  **Tel.: 04 72 07 17 62**  **Address : Service de Médecine Intensive Réanimation.103 grande rue de la Croix-Rousse. 69004 Lyon, FRANCE** |
| 1. **Public Title** | **Ultraprotective ventilation without extracorporeal circulation during COVID 19 pneumonia** |
| 1. **Scientific Title** | **Ultraprotective ventilation without extracorporeal circulation during COVID 19 pneumonia (VT4COVID)** |
| 1. **Countries of Recruitment** | **France** |
| 1. **Health Condition(s) or Problem(s) Studied** | **COVID-19 pneumonia under invasive mechanical ventilation** |
| 1. **Intervention(s)** | **Control group : protective ventilation (i.e. tidal volume 6 mL.kg^-1^ predicted body weight) applied until success of a deep sedation weaning trial**  **Intervention group : ultra-low tidal volume ventilation (i.e. tidal volume 4 mL.kg^-1^ predicted body weight) applied until success of a deep sedation weaning trial** |
| 1. **Key Inclusion and Exclusion Criteria** Inclusion and exclusion criteria for participant selection, including age and sex. Other selection criteria may relate to clinical diagnosis and co-morbid conditions; exclusion criteria are often used to ensure patient safety.  If the study is conducted in healthy human volunteers not belonging to the target population (e.g. a preliminary safety study), enter "healthy human volunteer". | **Inclusion criteria:**  - adults aged 18 years or older  - acute respiratory distress syndrome according to the Berlin definition  - COVID-19 pneumonia confirmed by a positive SARS-Cov-2 RT-PCR performed less than 7 days before inclusion  - invasive mechanical ventilation with PaO_2_/FiO_2_ ≤ 150 mmHg and positive end-expiratory pressure ≥ 5 cmH_2_O and with a tidal volume ≤ 6 mL.kg^-1^ predicted body weight  **Exclusion criteria:**  - onset of invasive or non-invasive ventilation more than 48 hours before inclusion  - previous inclusion in present study  - arterial pH < 7.21 despite respiratory rate set to 35 min^-1^ at the time of inclusion  - patient under any extracorporeal CO_2_ removal technique or extracorporeal membrane oxygenation  - pneumothorax or bronchopleural fistula  - suspected intracranial hypertension  - severe chronic obstructive pulmonary disease defined by a GOLD score ≥ 3  - chronic respiratory failure requiring long term oxygen or non-invasive ventilation  - obesity with body weight over height ratio greater than 1 kg.cm^-1^  - sickle cell disease  - bone marrow transplant < 6 months or neutropenia  - burn injury with extension greater than 30% of body surface area  - cirrhosis with Child-Pugh score C  - advance directives to withhold or withdraw life sustaining treatment  - Patient under an exclusion period relative to participation to another clinical trial, or current inclusion into another clinical trial sharing the same primary endpoint as the present study, or inclusion into a clinical trial involving unlicensed new drugs or medical devices. Modification of this criterion was accepted by the research ethics committee on January 22^th^, 2021, and co-inclusion into a clinical trial involving unlicensed new drugs or medical devices was no longer forbidden.  - pregnancy  - patient under a legal protective measure.  - lack of affiliation to social security as required by French regulation  - lack of written informed consent by patient or next of kins |
| 1. **Study Type** Study type consists of: | **Interventional multicenter prospective open labelled, randomized controlled superiority trial, with two parallel groups and balanced randomization using** **a randomization list stratified by center, with a 1:1 ratio, using random blocks of size 4 and 6** |
| 1. **Date of First Enrollment** | **15/04/2020** |
| 1. **Sample Size** |  |
| - - Number of participants that the trial plans to enroll in total. | **200.** To account for premature exits from the study (withdrawal of informed consent for example) we have planned to add 10% more patients (10 supplementary patients in each group for a total of 220 patients). |
| - - Number of participants that the trial has enrolled. | **220** |
| 1. **Recruitment Status** Recruitment status of this trial: | **Complete on 04/13/2021** |
| 1. **Primary Outcome(s)** |  |
| - - The name of the outcome | **A composite score based on 90-day all-cause mortality as first criterion and the number of ventilator-free days at day-60 after inclusion as second criterion** |
| - - The metric or method of measurement used (be as specific as possible) | **The composite score will be obtained by comparing each patient of one group to all patients of the other group. For each pair, a value of +1 (favorable), -1 (unfavorable) or 0 (neutral) will be given to each patient. The score will be built as following:**   - **a value of +1 will be given for a patient alive at day-90 paired with a patient deceased at day-90** - **a value of +1 will be given for a patient alive at day-90 paired with a patient alive at day-90 but with a lower number of VFD.** - **a value of 0 will be given for pairs with both patients deceased at day-90** - **a value of 0 will be given in case of identical number of VFD for the pairs of patients alive at day-90** - **a value of -1 will be given for a deceased patient in comparisons to patients alive at day-90** - **a value of -1 will be given for a patient alive at day-90 paired with a patient alive at day-90 but with a higher number of VFD**   **For a given patient the score will correspond to the sum of values resulting to the comparison to all patients of the other group.**  **VFD will be computed as follows from the day of inclusion :**   - **VFD = 0 if the patient dies between inclusion and day-60** - **VFD = 60-x if the patient is successfully weaned from invasive mechanical ventilation, with x being the number of days from inclusion to last successful extubation. Successful weaning from mechanical ventilation will be defined as extubation without reintubation within at least 48 hours (or weaning from mechanical ventilation for at least 48 hours for patients with tracheostomy)** - **VFD = 0 if the patient is mechanically ventilated for more than 60 days after inclusion** |
| - - The timepoint(s) of primary interest | **Day 60 after inclusion for ventilator free days (1^st^ component of the composite score and day 90 after inclusion for mortality (2^nd^ component of the composite score** |
| 1. **Key Secondary Outcomes** Secondary outcomes are outcomes which are of secondary interest or that are measured at timepoints of secondary interest. A secondary outcome may involve the same event, variable, or experience as the primary outcome, but measured at timepoints other than those of primary interest.  As for primary outcomes, for each secondary outcome provide: |  |
| - - The name of the outcome   - The metric or method of measurement used   - The timepoint(s) of interest | **90-day all-cause mortality**  **Percentage of included patients**  **90-day after inclusion** |
| - - The name of the outcome   - The metric or method of measurement used   - The timepoint(s) of interest | **Ventilator-free days at day-60 after inclusion**  **Days**  **60-day after inclusion** |
| - - The name of the outcome   - The metric or method of measurement used   - The timepoint(s) of interest | **Time from inclusion to successful extubation**  **Days**  **Mechanical ventilation** |
| - - The name of the outcome   - The metric or method of measurement used   - The timepoint(s) of interest | **Length of hospital stay from inclusion**  **Days**  **Hospital stay** |
| - - The name of the outcome   - The metric or method of measurement used   - The timepoint(s) of interest | **Value of daily sedation dose (midazolam, propofol and opioid)**  **Dose in mg**  **First 14 days after inclusion** |
| - - The name of the outcome   - The metric or method of measurement used   - The timepoint(s) of interest | **Rate of use of rescue therapy (i.e. muscle relaxant, prone position, inhaled nitric oxide, recruiting maneuvers, ECMO)**  **Percentage of included patients**  **First 14 days after inclusion** |
| - - The name of the outcome   - The metric or method of measurement used   - The timepoint(s) of interest | **Severe mixed acidosis, ventilator associated pneumonia, acute cor pulmonale, barotrauma and any serious adverse events**  **Percentage of included patients**  **First 28 days after inclusion** |
| - - The name of the outcome   - The metric or method of measurement used   - The timepoint(s) of interest | **Montreal Cognitive Assessment (T-MoCA) test**  **Score ranging from 0 to 30**  **Day-365 after inclusion** |
| - - The name of the outcome   - The metric or method of measurement used   - The timepoint(s) of interest | **SF-36 score**  **Score ranging from 0 to 100**  **Day-365 after inclusion** |
| - - The name of the outcome   - The metric or method of measurement used   - The timepoint(s) of interest | **IES-R score**  **Score ranging from 0 to 88**  **Day-365 after inclusion** |
| - - The name of the outcome   - The metric or method of measurement used   - The timepoint(s) of interest | **Incremental cost-effectiveness ratios of the innovative strategy compared to the reference strategy**  **Ratio**  **Day-90 after inclusion.** |
| 1. **Ethics Review**: |  |
| - - Status | **Approved** |
| - - Date of approval | **April 6th, 2020** |
| - - Name and contact details of Ethics committee(s) | **CPP Ile de France 7 CHU de BICETRE – 78 rue du Général Leclerc – 94275 LE KREMLIN BICETRE CEDEX** |
| 1. **Completion date** | **07/12/2021** |
| 1. **Summary Results** It consists of: |  |
| - - Date of posting of results summaries | **Planned on fourth quarter of 2021** |
| - - Date of the first journal publication of results | **Planned on fourth quarter of 2021** |
| - - URL hyperlink(s) related to results and publications | **Planned on fourth quarter of 2021** |
| - - Baseline Characteristics: Data collected at the beginning of a clinical study for all participants and for each arm or comparison group. These data include demographics, such as age and sex, and study-specific measures. | **Will be included in journal publication of the study results** |
| - - Participant flow: Information to document the progress and numbers of research participants through each stage of a study in a flow diagram or tabular format. | **Will be included in journal publication of the study results** |
| - - Adverse events: An unfavorable change in the health of a participant, including abnormal laboratory findings, and all serious adverse events and deaths that happen during a clinical study or within a certain time period after the study has ended. This change may or may not be caused by the intervention being studied. | **Will be included in journal publication of the study results** |
| - - Outcome measures: A table of data for each primary and secondary outcome measure and their respective measurement of precision (eg a 95% confidence interval) by arm (that is, initial assignment of participants to arms or groups) or comparison group (that is, analysis groups), including the result(s) of scientifically appropriate statistical analyses that were performed on the outcome measure data, if any. | **Will be included in journal publication of the study results** |
| - - URL link to protocol file(s) with version and date | **Is included in the current publication** |
| - - Brief summary | **Will be included in journal publication of the study results** |
| 1. **IPD sharing statement** Statement regarding the intended sharing of deidentified individual clinical trial participant-level data (IPD). Should indicate whether or not IPD will be shared, what IPD will be shared, when, by what mechanism, with whom and for what types of analyses. It consists of: |  |
| - - Plan to share IPD (Yes, No) | **IPD of the whole dataset will be shared on reasonable request.** |
| - - Plan description | **Plan description will be shared on reasonable request.** |
