# Supplementary material for: Open-label randomized controlled trial of ultra-low tidal ventilation without extracorporeal circulation in patients with COVID-19 pneumonia and moderate to severe ARDS: study protocol for the VT4COVID trial
Source: Trials. 2021 Oct 11;22:692. doi: 10.1186/s13063-021-05665-z (PMC8503716; doi:10.1186/s13063-021-05665-z)
Supplement: Supplementary file 4 — Additional file 4. Study sites. [file 13063_2021_5665_MOESM4_ESM.docx]

Study sites

1. Service de Médecine Intensive Réanimation. Hôpital de la Croix-Rousse – Hospices Civils de Lyon, Lyon (FRANCE)
2. Service de Médecine Intensive Réanimation. Hôpital Michallon - CHU Grenoble Alpes, Grenoble (FRANCE)
3. Service de Réanimation Polyvalente. Centre Hospitalier Lyon Sud – Hospices Civils de Lyon, Lyon (FRANCE)
4. Service de Médecine Intensive Réanimation. CHU Gabriel Montpied, Clermont-Ferrand (FRANCE)
5. Service de Médecine Intensive Réanimation. Hôpital Edouard Herriot – Hospices Civils de Lyon, Lyon (FRANCE)
6. Service de Médecine Intensive Réanimation. Hôpital Nord – CHU Saint-Etienne, Saint-Etienne (FRANCE)
7. Service de Réanimation. Clinique de la Sauvegarde, Lyon (FRANCE)
8. Service de réanimation Polyvalente. Centre Hospitalier Saint Joseph-Saint Luc, Lyon (FRANCE)
9. Service de Réanimation Chirurgicale. Hôpital Edouard Herriot – Hospices Civils de Lyon, Lyon (FRANCE)
10. Service de Réanimation. Centre Hospitalier Annecy Genevois, Pringy (FRANCE)
11. Service de Réanimation Chirurgicale. Hôpital de la Croix-Rousse – Hospices Civils de Lyon, Lyon (FRANCE)
