# Supplementary material for: Open-label randomized controlled trial of ultra-low tidal ventilation without extracorporeal circulation in patients with COVID-19 pneumonia and moderate to severe ARDS: study protocol for the VT4COVID trial
Source: Trials. 2021 Oct 11;22:692. doi: 10.1186/s13063-021-05665-z (PMC8503716; doi:10.1186/s13063-021-05665-z)
Supplement: Supplementary file 5 — Additional file 5. Protocol summarized version. [file 13063_2021_5665_MOESM5_ESM.docx]

SEVRAGE DE LA SEDATION LOURDE


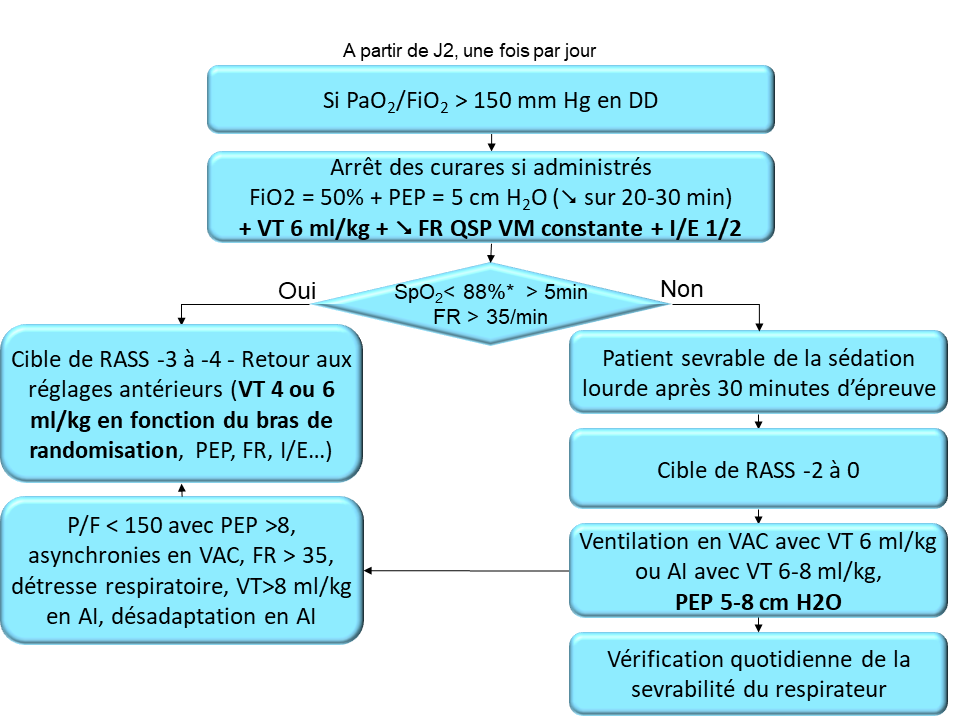


SEVRABILITE DU RESPIRATEUR


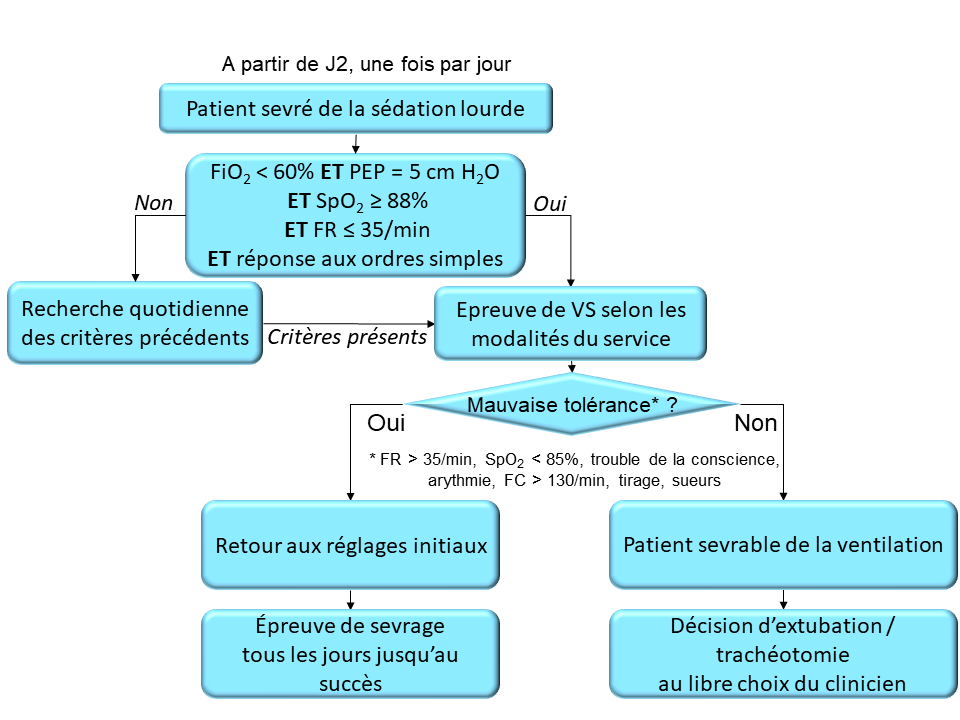


Contacts

- Investigateur coordonnateur : Dr Hodane YONIS

Téléphone : 04 26 10 92 71.

Email : hodane.yonis@chu-lyon.fr

- Investigateur associé : Pr Jean-Christophe RICHARD

Téléphone : 04 26 10 92 72.

Email : [j-christophe.richard@chu-lyon.fr](mailto:j-christophe.richard@chu-lyon.fr)

- Coordinatrice d’études cliniques : Loredana BABOI

Tel : 04 26 10 92 65. Fax : 04 72 07 17 74.

Email : [loredana.baboi@chu-lyon.fr](mailto:loredana.baboi@chu-lyon.fr)

VT4-COVID

Ventilation ultraprotectrice chez les patients avec pneumonie à COVID-19 et SDRA modérément sévère à sévère

# CRITERES D’INCLUSION

1. Adulte âgé d’au moins 18 ans

2. Intubation et ventilation mécanique

3. Pneumonie à COVID-19 confirmée par RT-PCR de moins de 7 j

4. IRA hypoxémique non complètement expliquée par une insuffisance ventriculaire gauche ou une surcharge hydrosodée

5. Opacités pulmonaires bilatérales non expliquées par des épanchements pleuraux ou des atélectasies ou des nodules

6. Ventilation mécanique invasive avec PaO2/FiO2 ≤ 150 mm Hg et PEP ≥ 5cm H2O avec un VT ≤ 6 ml/kg de PPT

7. Sédation intraveineuse continue

# CRITERES D’EXCLUSION

1. Ventilation mécanique invasive ou non-invasive > 48 heures
2. Patient précédemment inclus dans le même protocole
3. pH artériel < 7.21 malgré une FR à 35/min
4. Traitement par ECMO ou épuration extracorporelle de CO2
5. Pneumothorax ou fistule bronchopleurale
6. Hypertension intracrânienne (suspectée ou confirmée)
7. BPCO connue définie par un score de GOLD score ≥ 3
8. Insuffisance respiratoire chronique avec indication d’oxygénothérapie ou VNI long cours (hypoxémie chronique)
9. Obésité morbide définie par un poids supérieur à 1 kg/cm
10. Drépanocytose
11. Greffe de moelle récente, aplasie post-chimiothérapie
12. Brûlure étendue (> 30% de la surface corporelle)
13. Cirrhose hépatique grave (Child-Pugh C)
14. Décision de limitation des thérapeutiques actives
15. Patient participant à une autre recherche de catégorie 1, ou inclus dans une recherche partageant le même critère de jugement principal que la présente étude
16. Grossesse ou allaitement
17. Patient majeur protégé au sens de la loi
18. Patient non bénéficiaire d’un régime de sécurité sociale
19. Consentement de participation non obtenu sauf si recours à la procédure d’urgence en l’absence de proche

OBJECTIFS VENTILATOIRES DANS LES 2 BRAS

- Pression plateau ≤ 30 cm H_2_O (occlusion télé-inspiratoire 3s)

- 60 ≤ PaO_2_ ≤ 80 mm Hg ou 88% ≤ SpO_2_ ≤ 95%

- 7,20 ≤ pH artériel ≤ 7,45

REGLAGES DU RESPIRATEUR A L’INCLUSION

a. mode volume assisté contrôlé

b. enlever le raccord annelé et le remplacer par un raccord minimisant l’espace mort

c. humidification des gaz par humidificateur chauffant (sans ECH)

d. BRAS VENTILATION ULTRAPROTECTRICE : ⭨ le VT de 1 ml/kg de poids prédit par la taille (PPT) par intervalles ≤ 2heures jusqu’à 4 ml/kg de PPT.

BRAS VENTILATION PROTECTRICE : Régler le VT à 6 ml/kg de PPT

PPT (kg) = Taille (cm) - 152,4) × 0.91 + 50 chez l’homme

PPT (kg) = Taille (cm) - 152,4) × 0.91 + 45,5 chez la femme

e. fréquence respiratoire (FR) ajustée jusqu’à 35/min maximum pour maintenir ou se rapprocher du volume minute pré- inclusion.

f. I/E entre 1/2 et 1/4 pour minimiser la PEP intrinsèque.

g. ajustement de la PEP selon la **table PEP-FiO_2_** pour obtenir les objectifs d’oxygénation

| PEP | 5 | 5 | 8 | 8 | 10 | 10 | 10 | 12 | 14 | 14 | 14 | 16 | 18 | 20 | 22 | 24 |
| --- | --- | --- | --- | --- | --- | --- | --- | --- | --- | --- | --- | --- | --- | --- | --- | --- |
| FiO_2_ | 30 | 40 | 40 | 50 | 50 | 60 | 70 | 70 | 70 | 80 | 90 | 90 | 90 | 100 | 100 | 100 |

- **Si la PEP et la FIO_2_ ne sont pas compatibles avec la table** (i. e. juste après randomisation ou modification urgente de la PEP ou de la FiO2 après une ⭨ de la SPO2), l’ajustement de la PEP et de la FiO_2_ se fait par modification toutes les 5 minutes pour aboutir à la PEP et la FIO_2_ indiquées par la table.

**- ATTENTION, à la montée de PEP, si Pplat > 30 cm H2O, revenir au niveau de PEP précédent**

**-SI LA PEP EST MAL TOLEREE, NE PAS SUIVRE LA TABLE**

DE l’INCLUSION JUSQU’A SEVRABILITE DE LA SEDATION LOURDE

# REGLAGES DES ALARMES DU RESPIRATEUR

Les alarmes seront réglées afin de détecter les asynchronies

- Alarme de VT réglée à 1,5 fois le VT en VAC afin de détecter les doubles déclenchements
- Alarme de FR réglée à 37/min pour détecter auto-déclenchements, désadaptations du respirateur, et polypnée

# TRAITEMENTS RESPIRATOIRES ADJUVANTS

1. Gestion de la curarisation
   - Curarisation initiale pendant au moins 48 heures à la phase initiale du SDRA avec PaO_2_/FiO_2_ < 150 mm Hg
   - Tentative d’arrêt quotidien après J2 si PaO2/FiO2 ≥ 100 mm Hg et PEP ≤ 10 cm H2O
2. DV : première séance de 16 h si PaO_2_/FiO_2_ < 150 mm Hg, et poursuite des séances de 16h/24H si PaO_2_/FiO_2_ < 150 mm de Hg **OU** PEP > 10 cmH_2_O **OU** FiO_2_ > 60% après le retour en DD.
3. Sédation réévaluée toutes les 4H avec objectif de RASS

| Situation clinique | Score RASS cible |
| --- | --- |
| curarisation | entre -4 et -5 |
| décubitus ventral | entre -4 et -5 |
| sédation lourde | entre -3 et -4 |
| après sevrage de la sédation lourde | entre 0 et -2 |

# Si PaO_2_ < 60 mm Hg ou SpO_2_ < 88%

a. gestion de la PEP et de la FiO_2_ selon la table PEP-FiO_2_, avec contrôle 15 min plus tard de la SpO_2_

b. DV si PaO_2_/FiO_2_<150 mm Hg OU PEP > 10 cm H_2_O OU FiO_2_ > 60%

c. réintroduction éventuelle des curares si PaO_2_/FiO_2_<150 mm Hg

d. NO inhalé 10 ppm réévalué 24 heures plus tard maximum

e. manœuvres de recrutement non recommandées

f. considérer l’ECMO

# Si PaO_2_ > 80 mm Hg ou SpO_2_ > 95%

a. arrêt du NO inhalé si administré

b. gestion de la PEP et de la FiO_2_ selon la table PEP-FiO_2_, avec contrôle 15 minutes plus tard de la SpO_2_

# Si Pplat > 30 cm H_2_O

1. reprise de la curarisation avec bolus initial si efforts inspiratoires visibles
2. ⭨ le VT par paliers de 1 ml/kg toutes les 5 min tant que Pplat > 30 cmH_2_O jusqu’à 4 ml/kg (si pH<7,2, le VT n’est pas diminué)
3. ⭨ la PEP jusqu’à 5 cm d’H_2_O pour maintenir Pplat ≤ 30 cm H_2_O

# Si pH < 7,20

a. adapter la sédation / curarisation pour obtenir une bonne adaptation patient- respirateur

b. enlever le raccord annelé s’il est présent

c. ⭧ la FR sans dépasser 35/min.

d. perfusion éventuelle de bicarbonates

e. si le pH reste < 7,15, ⭧ le VT par palier de 1 ml/kg de PPT jusqu’à obtenir un pH ≥ 7,20 sans dépasser 8 ml/kg de PPT.

f. considérer l’épuration extracorporelle de CO_2_ ou l’ECMO.

# Si pH > 7,45

a. **bras ventilation ultraprotectrice** : ⭨ le VT à 4 ml/kg de PPT

**bras ventilation protectrice** : ⭨le VT à 6 ml/kg de PPT

b. ⭨ la fréquence respiratoire

# Si VT > 4ml/kg de PPT ET pH > 7,20 (V. ultraprotectrice)

Tenter de diminuer le VT par paliers de 0,5 à 1 ml/kg de PPT jusqu’à 4 ml/kg de PPT (au moins biquotidiennement)

# Si VT > 6ml/kg de PPT ET pH > 7,20 (V. protectrice)

Tenter de diminuer le VT par paliers de 0,5 à 1 ml/kg de PPT jusqu’à 6 ml/kg de PPT (au moins biquotidiennement)

# Si pneumothorax

Le niveau de PEP est laissé au libre choix du clinicien, les autres recommandations de l’étude devant s’appliquer.

# Si FR > 35 /min ou asynchronies patient-machine

A. **Si administration continue de curares**: bolus de curares et augmentation de 20% de leur dose

B. **Si PaO2/FIO2<150 mm Hg OU PEP > 8 cm H2O**

1. Bolus de sédation et de morphinique associé à une reprise de leur administration continue ou ⭧de leur dose continue
2. Bolus de curares et reprise de leur administration en continu

C. **Si PaO2/FIO2≥150 mm Hg avec PEP ≤ 8 cm H2O en DD**

1. Ajuster le rapport I/E (réduire jusqu’à 1/4 si dépression de la courbe de pression à l’inspiration, allonger si double déclenchement sans aller au-delà d’un I/E à ½)
2. Bolus de sédation et de morphinique
3. Si persistance des asynchronies après 15 min OU FR>35/min, passage en Aide Inspiratoire (VT cible 6 à 8 ml/kg de PPT)
4. si VT > 8 ml/kg de PPT en aide inspiratoire ou FR >35/min ou détresse respiratoire

- nouveau bolus de sédation et de morphinique ET **reprise de la sédation lourde** (objectif de RASS -3 à -4)

- ⭨ le VT à 4ml/kg **dans le bras Ventilation Ultraprotectrice**

1. Si persistance des asynchronies après 15 min ou FR>35/min, bolus de curares et reprise de leur administration continue
