# Supplementary material for: Open-label randomized controlled trial of ultra-low tidal ventilation without extracorporeal circulation in patients with COVID-19 pneumonia and moderate to severe ARDS: study protocol for the VT4COVID trial
Source: Trials. 2021 Oct 11;22:692. doi: 10.1186/s13063-021-05665-z (PMC8503716; doi:10.1186/s13063-021-05665-z)
Supplement: Supplementary file 6 — Additional file 6. Case report form. [file 13063_2021_5665_MOESM6_ESM.pdf]

Hospices Civils de Lyon

ETUDE VT4-COVID

Ventilation avec ultra faible volume courant chez les patients avec pneumonie à COVID-19 et SDRA modérément sévère à sévère – Etude randomisée contrôlée en ouvert

CAHIER D'OBSERVATION

V1.2 du 20/04/2020

Code patient |___|___| - |___|___| - |___|___|___|

Numéro de centre   Initiales   Numéro d'inclusion

Investigateur Coordonnateur :

YONIS Hodane, PH
Service de Médecine Intensive Réanimation
Groupement Hospitalier Nord
Hôpital de la Croix Rousse
103 Grande rue de la Croix Rousse
69004 Lyon
Téléphone : 04 72 07 17 62 ; Fax : 04 72 07 17 74.
Hodane.yonis@chu-lyon.fr

Promoteur :

Hospices Civils de Lyon
Délégation à la Recherche Clinique et à l'Innovation
3, quai des Célestins BP 2251
69229 Lyon Cedex 02

HCL HOSPICES CIVILS DE LYON	V1 : SCREENING
Code patient	ELIGIBILITE

Critères d'inclusion

CI1	Adulte âgé d'au moins 18 ans	□0 Non □1 Oui
CI2	Intubation et ventilation mécanique	□0 Non □1 Oui
CI3	Pneumonie à COVID-19 confirmée par RT-PCR sur prélèvement nasopharyngé ou du tractus respiratoire datant de moins de 7 jours	□0 Non □1 Oui
CI4	Insuffisance respiratoire aiguë non complètement expliquée par une insuffisance ventriculaire gauche ou une surcharge hydrosodée	□0 Non □1 Oui
CI5	Opacités radiologiques pulmonaires bilatérales non complètement expliquées par des épanchements pleuraux ou atélectasies ou des nodules	□0 Non □1 Oui
CI6	Ventilation mécanique invasive avec $PaO_2/FiO_2 \leq 150$ mm Hg et PEP ≥ 5 cm H_2O avec un VT ≤ 6 ml/kg de PPT	□0 Non □1 Oui
CI7	Sédation intraveineuse continue dans le cadre du traitement du SDRA	□0 Non □1 Oui

Critères de non-inclusion

Critères de non-inclusion relatifs à l'histoire de la maladie

CNI1	Ventilation mécanique invasive ou non-invasive depuis plus de 48 heures (oxygénothérapie à haut débit autorisée sans limite)	□0 Non □1 Oui
CNI2	Patient précédemment inclus dans le même protocole de recherche	□0 Non □1 Oui

Critères de non-inclusion relatifs à la sévérité de la maladie

CNI3	pH artériel < 7.21 malgré une fréquence respiratoire à 35/min au moment de l'inclusion	□0 Non □1 Oui
CNI4	Traitement par assistance extracorporelle (ECMO ou épuration de CO2)	□0 Non □1 Oui
CNI5	Pneumothorax ou fistule broncho-pleurale	□0 Non □1 Oui

HCL HOSPICES CIVILS DE LYON	*Visite d'inclusion*	
Code patient	ELIGIBILITE	

Critères de non-inclusion relatifs aux pathologies associées entraînant des risques particuliers

CNI6	Hypertension intracrânienne (suspectée ou confirmée)	□0 Non □1 Oui

Critères de non-inclusion relatifs aux comorbidités

CNI7	BPCO connue définie par un score de GOLD ≥ 3	□0 Non □1 Oui
CNI8	Insuffisance respiratoire chronique avec indication d'oxygénothérapie au long cours ou assistance ventilatoire au long cours (hypoxémie chronique)	□0 Non □1 Oui
CNI9	Obésité morbide définie par un poids supérieur à 1 kg/cm	□0 Non □1 Oui
CNI10	Drépanocytose	□0 Non □1 Oui
CNI11	Greffe de moelle récente, aplasie post-chimiothérapie	□0 Non □1 Oui
CNI12	Brûlure étendue (> 30% de la surface corporelle)	□0 Non □1 Oui
CNI13	Cirrhose hépatique grave (Child-Pugh C)	□0 Non □1 Oui
CNI14	Décision de limitation des thérapeutiques actives	□0 Non □1 Oui

Critères de non-inclusion relatifs à la réglementation

CNI15	Patient se trouvant en période d'exclusion suite à la participation à une autre recherche impliquant la personne humaine de catégorie 1 OU inclus dans une recherche impliquant la personne humaine de catégorie 1 OU inclus dans une recherche impliquant la personne humaine partageant le même critère de jugement principal que la présente étude.	□0 Non □1 Oui
CNI16	Grossesse ou femme en cours d'allaitement	□0 Non □1 Oui
CNI17	Patient majeur protégé au sens de la loi	□0 Non □1 Oui
CNI18	Patient non bénéficiaire d'un régime de sécurité sociale	□0 Non □1 Oui
CNI19	Consentement de participation non obtenu (soit auprès du patient lui-même, soit auprès d'un de ses proches, soit enfin auprès de la personne de confiance que le patient aurait préalablement désigné par écrit) sauf si recours à la procédure d'urgence en l'absence de proche	□0 Non □1 Oui

SI L'UNE DES REPONSES EST OUI, LE PATIENT NE PEUT PAS ETRE INCLUS DANS L'ETUDE

Le patient est-il incluable dans l'essai ? □0 Non □1 Oui

Code patient	CONSENTEMENT

SIGNATURE DU CONSENTEMENT

Recueil du consentement ☐$_1$Personne de confiance/Proche ☐$_2$Procédure d'urgence ☐$_3$Patient

En cas de procédure d'urgence

Date à laquelle l'attestation de procédure d'urgence a été signée

une personne de confiance a-t-elle pu être contactée *a postériori* ? ☐$_0$Non ☐$_1$Oui

Si oui, signature du consentement ? ☐$_0$Non ☐$_1$Oui

Date du recueil du consentement |__|__|/|__|__|/|__|__|__|__|

SIGNATURE DU FORMULAIRE DE CONFIRMATION PAR LE PATIENT

Le patient a-t-il retrouvé ses facultés pour donner son accord en cours d'étude ? ☐$_0$Non ☐$_1$Oui

Si oui, Le patient s'oppose-t-il à participer au protocole ? ☐$_0$Non ☐$_1$Oui

Si non, Date de signature du formulaire de confirmation |__|__|/|__|__|/|__|__|__|__|

HCL HOSPICES CIVILS DE LYON	**Ventilation innovante comparée à une ventilation standard chez les patients avec pneumonie à COVID-19 et SDRA modérément sévère à sévère**<br>**Etude randomisée contrôlée en ouvert**
Code patient	***Randomisation***

PATIENT
Initiales (Nom-Prénom) |___|___|
Date de naissance (mm/aaaa) |___|___|/|___|___|___|___|
Consentement ☐$_0$Non ☐$_1$Oui
Date de signature du consentement/attestation de procédure d'urgence |__|__|/|__|__|/|__|__|__|__|
**RANDOMISATION**
Date de randomisation |__|__|/|__|__|/|__|__|__|__|
Personne responsable de la randomisation |____|
Strate |__|__|__|
Centre |__|__|__|
**RÉSULTAT DE LA RANDOMISATION**
☐ Bras EXPERIMENTAL : Ventilation innovante<br><br>☐ Bras DE REFERENCE : Ventilation standard

HCL HOSPICES CIVILS DE LYON

Visite d'inclusion

Code patient	

Paramètres du screening pré-inclusion	
Date de naissance |__|__|/|__|__|__|__|	Sexe du patient ☐$_1$Homme ☐$_2$Femme
⍰-HCG ☐$_0$Négatif ☐$_1$Positif ☐$_2$Non applicable ☐$_3$Non fait	
Antécédent(s) médicaux ? ☐$_0$ Non ☐$_1$ Oui	→ Merci de compléter le tableau
Date RT-PCR SARS-Cov-2 |__|__|/|__|__|/|__|__|__|__|	
Résultat de la RT-PCR SARS-Cov-2 ☐$_1$Positif ☐$_0$Négatif ☐$_9$Indéterminé	
Date dernier/ère scanner ou radiographie thoracique : |__|__|/|__|__|/|__|__|__|__|	

Gaz du sang datant de moins de 4 heures		
pH |__|.|__|__|	PaCO2 (mmHg) | | | | | |	PaO2 (mmHg) |__|__|__|.|__|__|
Bicarbonates (mmol/L) |_|_|.|_|_|	SaO2 (%) | | | | | |	Lactates (g/L) |__|__|__|.|__|__|
Position du malade pendant les gaz du sang	☐$_1$ Décubitus dorsal	☐$_2$ Décubitus ventral
Réglage du VT à une valeur ≤ 6 ml/kg de PPT avec une PEP ≥ 5 cm H_2O	☐$_0$ Non ☐$_1$ Oui	

Paramètres ventilatoires correspondant au gaz du sang (avant la mise en place de la stratégie ventilatoire)		
Mode ventilatoire ☐$_1$ VAC ☐$_2$ AI ☐$_3$ APRV ☐$_4$ BIPAP ☐$_5$ Aide inspiratoire	Volume courant (ml) |__|__|__|	Fréq. respiratoire (/min) |__|__|
Niveau de PEP externe (cm H2O) |__|__|	Niveau de PEP totale du syst. respiratoire (cm H2O) |__|__|	
Pression plateau du syst. Respiratoire (cm H2O) |__|__|	Pression de crête du syst. respiratoire (cm H2O) |__|__|	
Débit inspiratoire (l/min) |__|__|__|	I/E |__|__| . |__|	FiO2 (%) |__|__|__|
PaO2/FiO2 |__|__| . |__|		

Données d'admission	
Date d'admission à l'hôpital |__|__|/|__|__|/|__|__|__|__|	
Origine du patient ☐$_1$Domicile ☐$_2$Service d'urgence ☐$_3$Service court séjour hors réanimation ☐$_1$Service de réanimation ☐$_0$ Service long séjour hors réanimation ☐$_9$Autre, précisez :	
Taille (cm) |__|__|__|	Poids mesuré à l'admission en réanimation (kg) |__|__|__|.|__|
DATE et HEURE d'admission en réanimation |__|__|/|__|__|/|__|__|__|__| |__|__|:|__|__|	
Poids mesuré à l'inclusion (kg) |_|_|_|.|_|	Le patient présente-t-il une immunosuppression ? ☐$_0$ Non ☐$_1$ Oui
Score de Mac Cabe ☐$_1$ Pas de maladie mortelle ☐$_2$ Maladie mortelle à 5 ans ☐$_3$ Maladie mortelle à 1	

Antécédents

Code patient	

Maladie chronique	☐0 Non ☐1 Oui
Maladie mortelle	☐0 Non ☐1 Oui
Insuffisance cardiaque stade III NYHA	☐0 Non ☐1 Oui
Insuffisance respiratoire sous oxygène à domicile	☐0 Non ☐1 Oui
Cancer non métastasé	☐0 Non ☐1 Oui
Hypertension portale	☐0 Non ☐1 Oui
Insuffisance cardiaque stade IV NYHA	☐0 Non ☐1 Oui
Insuffisance respiratoire déjà ventilé **insuffisance respiratoire chronique sous ventilation au long cours**	☐0 Non ☐1 Oui
Cancer métastasé	☐0 Non ☐1 Oui
Décompensation hémorragique de cirrhose	☐0 Non ☐1 Oui

	Visite d'inclusion
Code patient	

Données relevées à l'inclusion

Date et heure d'intubation (JJ/MM/AAAA) |__|__|/|__|__|/|__|__|__|__| |__|__|:|__|__|	
Facteurs de risque de SDRA	COVID-19<br>Pneumopathie communautaire<br>Inhalation de liquide gastrique<br>Pneumopathie acquise sous ventilation mécanique<br>Pneumopathie associée aux soins,<br>sepsis intra-abdominal<br>Sepsis extra-abdominal et extrapulmonaire<br>Pancréatite aiguë<br>Contusion pulmonaire<br>Noyade<br>Inhalation de fumée toxique<br>Polytransfusion<br>Traumatisme thoracique<br>Polytraumatisme<br>Circulation extracorporelle<br>Autre, précisez :

Visite d'inclusion

Code patient	

Paramètres hémodynamiques (avant la mise en place de la stratégie ventilatoire)		
Dose de noradrénaline	|__|__|__|__|	□1 µg/kg/min □2 mg/h
Dose d'adrénaline	|__|__|__|__|	□1 µg/kg/min □2 mg/h
Dose de dobutamine	|__|__|__|__|	□1 µg/kg/min □2 mg/h

Paramètres neurologiques (avant la mise en place de la stratégie ventilatoire)	
Score RASS	|__||__|
Score de Glasgow	|__|__|

Doses de sédation *(avant la mise en place de la stratégie ventilatoire)*	
Dose de midazolam (mg/H) |__|__|__|__|	Dose de propofol (mg/H) |__|__|__|__|
Dose de morphine (mg/H) |__|__|__|__|	Dose de fentanyl (µg/H) |__|__|__|__|
Dose de sufentanil (µg/H) |__|__|__|__|	
Type et dose de curare (molécule, dose en mg/h) ______________________ |__|__|__|__|	

Paramètres échocardiographiques (avant la mise en place de la stratégie ventilatoire)	
Date de la dernière échographie cardiaque	|__|__|/|__|__|/|__|__|__|__|
Rapport des surfaces ventriculaire droite et gauche	☐0 Non ☐1 Oui
Présence d'une dyskinésie septale	☐0 Non ☐1 Oui

Visite d'inclusion

Code patient	

Données clinico-biologiques (avant la mise en place de la stratégie ventilatoire)			
Plaquettes $10^3/mm^3$		|__|__|__|	☐ ND
Bilirubine	☐ µmol/L ☐ mg/dL	|__|__|__|__|.|__|	☐ ND
Créatinine	☐ µmol/L ☐ mg/dL	|__|__|__|.|__|__|	☐ ND
Diurèse des dernières 24 heures (mL)		|__|__|__|__|	☐ ND

IGS II et Score de SOFA (avant la mise en place de la stratégie ventilatoire)		
Score IGS II à l'admission en réanimation	|__|__|	→ Pour le calcul
Score de SOFA à l'inclusion	|__|__|	→ Pour le calcul

Traitements adjuvants du SDRA au cours des dernières 24 heures

☐1 Monoxyde d'azote inhalé

☐2 Décubitus ventral

☐3 Manœuvres de recrutement

☐4 Epuration extrarénale

☐5 ECMO

Evénements indésirables

Evénements indésirables entre la signature du consentement* et l'application de la stratégie de l'étude ? ☐0 Non ☐1 Oui

** ou signature de l'attestation de procédure d'urgence*

Si oui, merci de compléter la rubrique des événements indésirables

Procédure expérimentale de l'étude

Date/Heure de début de la procédure expérimentale de l'étude |__|__|/|__|__|/|__|__|__|__| |__|__|:|__|__|

SCORE IGS2
Admission Réanimation

Code patient	

Age (ans)	|__|__|
Fréquence cardiaque (bpm)	|__|__|__|
TA Systolique (mmHg)	|__|__|__|
Température (°C)	|__|__|.|__|
PaO_2/FiO_2 (seulement si V.M. ou CPAP)	|__|__|
Diurèse (L/jour)	|__|__|__|__|
Urée	☐$_0$ mmol/L ☐$_1$ g/L |__|__|.|__|
Globules Blancs ($10^{-3}/mm^2$)	|__|__|.|__|__|.
Kaliémie (mEq/L)	|__|__|.|__|
Natrémie (mEq/L)	|__|__|__|
HCO_3^- (mEq/L)	|__|__|
Bilirubine (µmol/L)	|__|__|__|
Score de Glasgow	|__|__|
Maladies chroniques	☐ Cancer métastasique ☐ Maladie Hématologique maligne ☐ SIDA ☐ NA
Type d'admission	☐ Maladie médical ☐ Chirurgie urgente ☐ Malade chirurgical programmé<br>☐ NK

HCL HOSPICES CIVILS DE LYON	***SCORE DE SOFA INCLUSION***
Code patient	

Respiratoire	PaO_2/FiO_2 (mmHg)	|__|__|
Neurologique	Score de Glasgow	|__|__|
Cardiovasculair	☐ Dopamine>15 µg/kg/min OU adrénaline >0,1 µg/kg/min OU Noradrénaline >0,1 µg/kg/min<br>□ PAM ≥ 70 mmHg<br>□ PAM < 70 mmHg<br>□ Dopamine ≤ 5 µg/kg/min ou Dobutamine Toute dose)(<br>☐ Dopamine>5 µg/kg/min OU adrénaline ≤ 0,1 µg/kg/min OU Noradrénaline ≤0,1 µg/kg/min	
Hépatique	Bilirubine (µmol/L)	|__|__|__|
Coagulation	Plaquette ($X10^3$/µl)	|__|__|__|
Rénal	Créatinine (mg/dL ou µmol/L) ou diuèrèse (mL)	|__|__|__|__|

Visites de suivi : de H2 à H18

Code patient	

	H4 ± 2h	*H10± 2h*	*H16 ± 2h*
Date ET heure de la mesure	|__|__|/|__|__|/|__|__|__|__| |__|__| : |__|__|	|__|__|/|__|__|/|__|__|__|__| |__|__| : |__|__|	|__|__|/|__|__|/|__|__|__|__| |__|__| : |__|__|
Paramètres ventilatoires			
Mode ventilatoire	□1 VAC □2 AI □3 APRV □4 BIPAP<br>□5 Aide inspiratoire	□1 VAC □2 AI □3 APRV □4 BIPAP<br>□5 Aide inspiratoire	□1 VAC □2 AI □3 APRV □4 BIPAP<br>□5 Aide inspiratoire
FIO_2 (%)	|__|__|__|	|__|__|__|	|__|__|__|
Volume courant (ml)	|__|__|	|__|__|	|__|__|
Fréquence respiratoire (/min)	|__|__|	|__|__|	|__|__|
Niveau de PEP externe (cm H2O)	|__|__|	|__|__|	|__|__|
Pression plateau du système respiratoire (cm H2O)	|__|__|	|__|__|	|__|__|
Pression de crête du système respiratoire (cm H2O)	|__|__|	|__|__|	|__|__|
Niveau de PEP totale du système respiratoire (cm H2O)	|__|__| ☐ Non mesurable	|__|__| ☐ Non mesurable	|__|__| ☐ Non mesurable
I/E	|__|__|	|__|__|	|__|__|
Débit inspiratoire (l/min)	|__|__|	|__|__|	|__|__|
Débit oxygène (L/min)	|__|__|	|__|__|	|__|__|

Visites de suivi : de H2 à H18

Code patient	

	H4 ± 2h	*H10± 2h*	*H16 ± 2h*
Score RASS	|__| |__|	|__| |__|	|__| |__|
Gaz du sang			
PaO2 (mmHg)	|__|__|__|.|__|__|	|__|__|__|.|__|__|	|__|__|__|.|__|__|
PaCO2 (mmHg)	|__|__|__|.|__|__|	|__|__|__|.|__|__|	|__|__|__|.|__|__|
pH	|__|.|__|__|	|__|.|__|__|	|__|.|__|__|
Bicarbonates (mmol/L)	|__|__|.|__|__|	|__|__|.|__|__|	|__|__|.|__|__|
SaO2 (%)	|__|__|__|.|__|__|	|__|__|__|.|__|__|	|__|__|__|.|__|__|
Position du malade pendant les gaz du sang	☐1 Décubitus dorsal<br>☐2 Décubitus ventral	☐1 Décubitus dorsal ☐2<br>Décubitus ventral	☐1 Décubitus dorsal<br>☐2 Décubitus ventral

Visites de suivi : de H2 à H18

Code patient	

	H4 ± 2h	*H10± 2h*	*H16 ± 2h*
Evénements indésirables			
Episode d’acidose respiratoire défini par un pH<7,15 avec paCO2 > 45 mm Hg	□$_0$ Non □$_1$ Oui		
Nouvelle pneumonie acquise sous ventilation mécanique avec initiation d’antibiothérapie	□$_0$ Non □$_1$ Oui		
Cœur pulmonaire aigu	□$_0$ Non □$_1$ Oui		
Barotraumatisme (pneumothorax, ou pneumomédiastin, ou emphysème sous cutané ou pneumatocèle de plus de 2 cm en imagerie)	□$_0$ Non □$_1$ Oui		
Autre événement indésirable	□$_0$ Non □$_1$ Oui		
**Si oui, merci de compléter la rubrique d’événements indésirables**			
Poursuite de la réanimation	□$_0$ Non □$_1$ Oui	□$_0$ Non □$_1$ Oui	□$_0$ Non □$_1$ Oui
**En cas de sortie de réanimation, merci de compléter la page « Sortie de réanimation » et, le cas échéant, la page sortie d’étude**			

Suivi J1

Code patient	

Données relevées 24 heures après l'inclusion (J1)	
Date |__|__|/|__|__|/|__|__|__|__|	Heure |__|__| : |__|__|
Poids |__|__|__|.|__|	
Paramètres ventilatoires	
Mode ventilatoire □1 VAC □2 AI □3 APRV □4 BIPAP □5 Aide inspiratoire	FIO_2 (%) |__|__|__| [21;100]
Volume courant (ml) |__|__|__|	Fréquence respiratoire (/min) |__|__|
Niveau de PEP externe (cm H2O)	|__|__|
Niveau de PEP totale du système respiratoire (cm H2O)	|__|__| ☐ Non mesurable
Pression de crête du système respiratoire (cm H2O)	|__|__|
Pression plateau du système respiratoire (cm H2O)	|__|__| ☐ Non mesurable
I/E) |__|__| . |__|	Débit inspiratoire (l/min) |__|__|__|
Paramètres hémodynamiques	
Dose de noradrénaline |__|__|__|__|	□1 µg/kg/min □2 mg/h
Dose d'adrénaline |__|__|__|__|	□1 µg/kg/min □2 mg/h
Dose de dobutamine |__|__|__|__|	□1 µg/kg/min □2 mg/h

Suivi J1

Code patient	

Paramètres neurologiques	
Score RASS	|__| |__|
Score de Glasgow	|__|__|
**Doses de sédation**	
Dose de midazolam (mg/H) |__|__|__|__|	Dose de propofol (mg/H) |__|__|__|__|
Dose de morphine (mg/H) |__|__|__|__|	Dose de fentanyl (µg/H) |__|__|__|__|
Dose de sufentanil (µg/H) |__|__|__|__|	
Type et dose de curare (molécule, dose en mg/h) __________________________ |__|__|__|__|	

Données clinico-biologiques		
Plaquettes	$10^3/mm^3$	|__|__|__|
Bilirubine	☐ µmol/L ☐ mg/dL	|__|__|__|__|.|__| ☐ ND
Créatinine	☐ µmol/L ☐ mg/dL	|__|__|__|.|__|__| ☐ ND
Diurèse des dernières 24 heures (mL)		|__|__|__|__|
Score SOFA		|__|__|

Gaz du sang	
PaO2 (mmHg) |__|__|__|.|__|__| ☐ ND	PaCO2 (mmHg) |__|__|__|.|__|__| ☐ ND
pH |__|.|__|__|	Bicarbonates (mmol/L) |__|__|.|__|__|
SaO2 (%) |__|__|__|.|__|__| ☐ ND	Lactates (mmol/L) |__|__|__|.|__|__| ☐ ND
Position du malade pendant les gaz du sang	☐1 Décubitus dorsal ☐2 Décubitus ventral

HCL HOSPICES CIVILS DE LYON	**_Suivi J1_**
Code patient	

Traitements adjuvants parmi les suivants au cours des dernières 24 heures	
□1 Monoxyde d'azote inhalé	□4 Epuration extrarénale
□2 Décubitus ventral	□5 ECMO
□3 Manœuvres de recrutement	□6 Bicarbonates IV (dose en grammes/24H))

Paramètres échocardiographiques	
Rapport des surfaces ventriculaire droite et gauche	□0 Non □1 Oui
Présence d'une dyskinésie septale	□0 Non □1 Oui

Intubation(s) et extubation(s)

*Le patient a-t-il été extubé/ré-intubé depuis l'inclusion ?** □0 Non □1 Oui

Si oui, combien de fois |__|

**y compris les auto-extubations*

Evènement indésirable depuis les dernières 24 heures	
Episode d'acidose respiratoire défini par un pH<7,15 avec paCO2 > 45 mm Hg	□0 Non □1 Oui
Nouvelle pneumonie acquise sous ventilation mécanique avec initiation d'antibiothérapie	□0 Non □1 Oui
Cœur pulmonaire aigu	□0 Non □1 Oui
Barotraumatisme (pneumothorax, ou pneumomédiastin, ou emphysème sous cutané ou pneumatocèle de plus de 2 cm en imagerie)	□0 Non □1 Oui
Autre événement indésirable	□0 Non □1 Oui
Si oui, merci de compléter la rubrique d'événements indésirables	
Poursuite de la réanimation	□0 Non □1 Oui

	**DE J2 À J14**
Code patient	**<u>À partir de J2</u>**, les données seront relevées le **<u>matin</u>**

Date |__|__|/|__|__|/|__|__|__|__|	Heure |__|__| : |__|__|
Poids |__|__|__|.|__|	
Paramètres ventilatoires	
Mode ventilatoire □1 VAC □2 AI □3 APRV □4 BIPAP □5 Aide inspiratoire	FIO_2 (%) |__|__|__| [21;100]
Volume courant (ml) |__|__|__|	Fréquence respiratoire (/min) |__|__|
Niveau de PEP externe (cm H2O)	|__|__|
Niveau de PEP totale du système respiratoire (cm H2O)	|__|__| ☐ Non mesurable
Pression de crête du système respiratoire (cm H2O)	|__|__|
Pression plateau du système respiratoire (cm H2O)	|__|__| ☐ Non mesurable
I/E |__|__| . |__|	Débit inspiratoire (l/min) |__|__|__|
Paramètres hémodynamiques	
Dose de noradrénaline |__|__|__|__|	□1 µg/kg/min □2 mg/h
Dose d'adrénaline |__|__|__|__|	□1 µg/kg/min □2 mg/h
Dose de dobutamine |__|__|__|__|	□1 µg/kg/min □2 mg/h

	**DE J2 À J14**
Code patient	

Paramètres neurologiques	
Score RASS	|__| |__|
Score de Glasgow	|__|__|
Doses de sédation	
Dose de midazolam (mg/H) |__|__|__|__|	Dose de propofol (mg/H) |__|__|__|__|
Dose de morphine (mg/H) |__|__|__|__|	Dose de fentanyl (µg/H) |__|__|__|__|
Dose de sufentanil (µg/H) |__|__|__|__|	
Type et dose de curare (molécule, dose en mg/h) ________________________ |__|__|__|__|	
Données clinico-biologiques	
Plaquettes $10^3/mm^3$ |__|__|__| ☐ ND	
Bilirubine ☐ µmol/L ☐ mg/dL	|__|__|__|__|.|__| ☐ ND
Créatinine ☐ µmol/L ☐ mg/dL	|__|__|__|.|__|__| ☐ ND
Diurèse des dernières 24 heures (mL)	|__|__|__|__| ☐ ND
Score SOFA	
Gaz du sang	
PaO2 (mmHg) |__|__|__|.|__|__| ☐ ND	PaCO2 (mmHg) |__|__|__|.|__|__| ☐ ND
pH |__|.|__|__|	Bicarbonates (mmol/L) |__|__|.|__|__|
SaO2 (%) |__|__|__|.|__|__| ☐ ND	Lactates (mmol/L) |__|__|__|.|__|__| ☐ ND
Position du malade pendant les gaz du sang	☐$_1$ Décubitus dorsal ☐$_2$ Décubitus ventral

HCL	**DE J2 À J14**
Code patient	

Traitements adjuvants parmi les suivants au cours des dernières 24 heures	
☐1 Monoxyde d'azote inhalé	☐4 Epuration extrarénale
☐2 Décubitus ventral	☐5 ECMO
☐3 Manœuvres de recrutement	☐6 Bicarbonates IV (dose en grammes/24H))

Intubation(s) et extubation(s)
*Le patient a-t-il été extubé/ré-intubé depuis la dernière visite ?** ☐0 Non ☐1 Oui<br>*Si oui, combien de fois I__I*<br>**y compris les auto-extubations*

Evènement indésirable depuis les dernières 24 heures	
Episode d'acidose respiratoire défini par un pH<7,15 avec paCO2 > 45 mm Hg	☐0 Non ☐1 Oui
Nouvelle pneumonie acquise sous ventilation mécanique avec initiation d'antibiothérapie	☐0 Non ☐1 Oui
Cœur pulmonaire aigu	☐0 Non ☐1 Oui
Barotraumatisme (pneumothorax, ou pneumomédiastin, ou emphysème sous cutané ou pneumatocèle de plus de 2 cm en imagerie)	☐0 Non ☐1 Oui
Autre événement indésirable	☐0 Non ☐1 Oui
Si oui, merci de compléter la rubrique d'événements indésirables	
Poursuite de la réanimation	☐0 Non ☐1 Oui

Visites de suivi : de J15 à J18

Code patient				
	J15	***J16***	***J17***	***J18***
Date				
*Le patient a-t-il été extubé/réintubé depuis l'inclusion ?**<br>*Si oui, combien de fois*	☐$_0$ Non ☐$_1$ Oui<br>|__|	☐$_0$ Non ☐$_1$ Oui<br>|__|	☐$_0$ Non ☐$_1$ Oui<br>|__|	☐$_0$ Non ☐$_1$ Oui<br>|__|
En cas d'extubation/ré-intubation, merci de compléter le tableau				
Episode d'acidose respiratoire défini par un pH<7,15 avec paCO2 > 45 mm Hg	☐$_0$ Non ☐$_1$ Oui	☐$_0$ Non ☐$_1$ Oui	☐$_0$ Non ☐$_1$ Oui	☐$_0$ Non ☐$_1$ Oui
Nouvelle pneumonie acquise sous ventilation mécanique avec initiation d'antibiothérapie	☐$_0$ Non ☐$_1$ Oui	☐$_0$ Non ☐$_1$ Oui	☐$_0$ Non ☐$_1$ Oui	☐$_0$ Non ☐$_1$ Oui
Cœur pulmonaire aigu	☐$_0$ Non ☐$_1$ Oui	☐$_0$ Non ☐$_1$ Oui	☐$_0$ Non ☐$_1$ Oui	☐$_0$ Non ☐$_1$ Oui
Barotraumatisme (pneumothorax, ou pneumomédiastin, ou emphysème sous cutané ou pneumatocèle de plus de 2 cm en imagerie)	☐$_0$ Non ☐$_1$ Oui	☐$_0$ Non ☐$_1$ Oui	☐$_0$ Non ☐$_1$ Oui	☐$_0$ Non ☐$_1$ Oui
Autre événement indésirable	☐$_0$ Non ☐$_1$ Oui<br>*Précisez :* ______________	☐$_0$ Non ☐$_1$ Oui<br>*Précisez :* ______________	☐$_0$ Non ☐$_1$ Oui<br>*Précisez :* ______________	☐$_0$ Non ☐$_1$ Oui<br>*Précisez :* ______________
**Si oui, merci de compléter la rubrique d'événements indésirables**				

Visites de suivi : de J19 à J22

Code patient				
	J19	***J20***	***J21***	***J22***
Date				
*Le patient a-t-il été extubé/réintubé depuis l'inclusion ?**<br>*Si oui, combien de fois*	☐$_0$ Non ☐$_1$ Oui<br>I__I	☐$_0$ Non ☐$_1$ Oui<br>I__I	☐$_0$ Non ☐$_1$ Oui<br>I__I	☐$_0$ Non ☐$_1$ Oui<br>I__I
En cas d'extubation/ré-intubation, merci de compléter le tableau				
Episode d'acidose respiratoire défini par un pH<7,15 avec paCO2 > 45 mm Hg	☐$_0$ Non ☐$_1$ Oui	☐$_0$ Non ☐$_1$ Oui	☐$_0$ Non ☐$_1$ Oui	☐$_0$ Non ☐$_1$ Oui
Nouvelle pneumonie acquise sous ventilation mécanique avec initiation d'antibiothérapie	☐$_0$ Non ☐$_1$ Oui	☐$_0$ Non ☐$_1$ Oui	☐$_0$ Non ☐$_1$ Oui	☐$_0$ Non ☐$_1$ Oui
Cœur pulmonaire aigu	☐$_0$ Non ☐$_1$ Oui	☐$_0$ Non ☐$_1$ Oui	☐$_0$ Non ☐$_1$ Oui	☐$_0$ Non ☐$_1$ Oui
Barotraumatisme (pneumothorax, ou pneumomédiastin, ou emphysème sous cutané ou pneumatocèle de plus de 2 cm en imagerie)	☐$_0$ Non ☐$_1$ Oui	☐$_0$ Non ☐$_1$ Oui	☐$_0$ Non ☐$_1$ Oui	☐$_0$ Non ☐$_1$ Oui
Autre événement indésirable	☐$_0$ Non ☐$_1$ Oui<br>*Précisez :*	☐$_0$ Non ☐$_1$ Oui<br>*Précisez :*	☐$_0$ Non ☐$_1$ Oui<br>*Précisez :*	☐$_0$ Non ☐$_1$ Oui<br>*Précisez :*
**Si oui, merci de compléter la rubrique d'événements indésirables**				

Visites de suivi : de J23 à J26

Code patient				
	J23	***J24***	***J25***	***J26***
Date				
*Le patient a-t-il été extubé/réintubé depuis l'inclusion ?** *Si oui, combien de fois*	☐$_0$ Non ☐$_1$ Oui I__I	☐$_0$ Non ☐$_1$ Oui I__I	☐$_0$ Non ☐$_1$ Oui I__I	☐$_0$ Non ☐$_1$ Oui I__I
En cas d'extubation/ré-intubation, merci de compléter le tableau				
Episode d'acidose respiratoire défini par un pH<7,15 avec paCO2 > 45 mm Hg	☐$_0$ Non ☐$_1$ Oui	☐$_0$ Non ☐$_1$ Oui	☐$_0$ Non ☐$_1$ Oui	☐$_0$ Non ☐$_1$ Oui
Nouvelle pneumonie acquise sous ventilation mécanique avec initiation d'antibiothérapie	☐$_0$ Non ☐$_1$ Oui	☐$_0$ Non ☐$_1$ Oui	☐$_0$ Non ☐$_1$ Oui	☐$_0$ Non ☐$_1$ Oui
Cœur pulmonaire aigu	☐$_0$ Non ☐$_1$ Oui	☐$_0$ Non ☐$_1$ Oui	☐$_0$ Non ☐$_1$ Oui	☐$_0$ Non ☐$_1$ Oui
Barotraumatisme (pneumothorax, ou pneumomédiastin, ou emphysème sous cutané ou pneumatocèle de plus de 2 cm en imagerie)	☐$_0$ Non ☐$_1$ Oui	☐$_0$ Non ☐$_1$ Oui	☐$_0$ Non ☐$_1$ Oui	☐$_0$ Non ☐$_1$ Oui
Autre événement indésirable	☐$_0$ Non ☐$_1$ Oui *Précisez :*	☐$_0$ Non ☐$_1$ Oui *Précisez :*	☐$_0$ Non ☐$_1$ Oui *Précisez :*	☐$_0$ Non ☐$_1$ Oui *Précisez :*
**Si oui, merci de compléter la rubrique d'événements indésirables**				

Visites de suivi : de J27 à J28

Code patient		
	J27	***J28***
Date		
*Le patient a-t-il été extubé/réintubé depuis l'inclusion ?** *Si oui, combien de fois*	☐$_0$ Non ☐$_1$ Oui I__I	☐$_0$ Non ☐$_1$ Oui I__I
En cas d'extubation/ré-intubation, merci de compléter le tableau		
Episode d'acidose respiratoire défini par un pH<7,15 avec paCO2 > 45 mm Hg	☐$_0$ Non ☐$_1$ Oui	☐$_0$ Non ☐$_1$ Oui
Nouvelle pneumonie acquise sous ventilation mécanique avec initiation d'antibiothérapie	☐$_0$ Non ☐$_1$ Oui	☐$_0$ Non ☐$_1$ Oui
Cœur pulmonaire aigu	☐$_0$ Non ☐$_1$ Oui	☐$_0$ Non ☐$_1$ Oui
Barotraumatisme (pneumothorax, ou pneumomédiastin, ou emphysème sous cutané ou pneumatocèle de plus de 2 cm en imagerie)	☐$_0$ Non ☐$_1$ Oui	☐$_0$ Non ☐$_1$ Oui
Autre événement indésirable	☐$_0$ Non ☐$_1$ Oui *Précisez :* _____________	☐$_0$ Non ☐$_1$ Oui *Précisez :* _____________
**Si oui, merci de compléter la rubrique d'événements indésirables**		

HCL HOSPICES CIVILS DE LYON	***Sortie de réanimation***
Code patient	

Données relevées à la sortie de réanimation	
Date de sortie de réanimation	|__|__|/|__|__|/|__|__|__|__|
Statut du patient	☐0 Vivant<br>☐1 Décédé (toute cause)
Date d'extubation avec succès<br><br>*définie comme une extubation sans ré-intubation dans les 48 heures ou l'arrêt de la ventilation invasive pendant plus de 48 heures chez les patients trachéotomisés*	|__|__|/|__|__|/|__|__|__|__|
Date d'arrêt des curares en administration continue<br><br>*définie comme l'absence de reprise de curare en perfusion continue pendant plus de 48h*	|__|__|/|__|__|/|__|__|__|__|

HCL	***Suivi J60 post-inclusion***
Code patient	

Données relevées J60 après l'inclusion	
Date J60	|__|__|/|__|__|/|__|__|__|__|
☐0 Non réalisé	Motif :
Le patient a-t-il été extubé/ré-intubé depuis J28 ?	☐0 Oui ☐1 Non
Si oui, nombre d'extubations	|__|
En cas d'extubation/ré-intubation, merci de compléter le tableau ___________	
Le patient a-t-il à nouveau séjourné en réanimation depuis la dernière visite ?	☐0 Oui ☐1 Non
Statut du patient	☐0 Vivant<br>☐1 Décédé (toute cause)

	Suivi J90 post-inclusion (+/- 2 jours)
Code patient	

Données relevées J90 après l'inclusion	
Date J90	|__|__|/|__|__|/|__|__|__|__|
☐$_0$ Non réalisé	Motif :
Le patient a-t-il à nouveau séjourné en réanimation depuis la dernière visite ?	☐$_0$ Oui ☐$_1$ Non
Statut du patient	☐$_0$ Vivant<br>☐$_1$ Décédé (toute cause)
Date de sortie de l'hôpital	|__|__|/|__|__|/|__|__|__|__|

Suivi J365 post-inclusion (+/- 10 jours)

Code patient	

Données relevées J365 après l'inclusion	
Date J365 |__|__|/|__|__|/|__|__|__|__|	☐$_0$ Non réalisé<br>Motif :
Le patient a-t-il à nouveau séjourné en réanimation depuis la dernière visite ?	☐$_0$ Oui ☐$_1$ Non
Statut du patient	☐$_0$ Vivant<br>☐$_1$ Décédé (toute cause)
Score de trouble cognitif T-MOCA	Merci de compléter le questionnaire
Score de qualité de vie SF-36	Merci de compléter le questionnaire
Score IES-R	Merci de compléter le questionnaire

Sortie d'étude

Code patient	

Sortie d'étude	☐$_0$ Normale ☐$_1$ Prématurée
Date de sortie d'étude	|__|__|/|__|__|/|__|__|__|__|

Pour toute sortie prématurée, indiquez la raison	
☐$_1$	Retrait de consentement
☐$_2$	Interruption temporaire ou définitive de la participation d'un sujet à l'étude pour toute raison qui servirait au mieux les intérêts du sujet en particulier en cas d'événements indésirables graves
☐$_3$	Sujet perdu de vue → Date du dernier contact :
☐$_4$	Screen failure
☐$_5$	Décès → Date du décès :
☐$_6$	Autre, précisez

EXTUBATION(S)-REINTUBATION(S)

Code patient	

EXTUBATION	DATE EXTUBATION	RE-INTUBATION	SI OUI, DATE DE RE-INTUBATION	SI NON, PRECISEZ
☐1 Décision médicale<br>☐2 Auto-extubation	|__|__|/|__|__|/|__|__|__|__|	☐0 Oui ☐1 Non	☐1 *Identique* *Si non, date*|__|__|/|__|__|/|__|__|__|__|	☐1 Extubation avec succès<br>☐2 Arrêt thérapeutique (Echec)<br>☐9 Autre :
☐1 Décision médicale<br>☐2 Auto-extubation	|__|__|/|__|__|/|__|__|__|__|	☐0 Oui ☐1 Non	☐1 *Identique* *Si non, date*|__|__|/|__|__|/|__|__|__|__|	
☐1 Décision médicale<br>☐2 Auto-extubation	|__|__|/|__|__|/|__|__|__|__|	☐0 Oui ☐1 Non	☐1 *Identique* *Si non, date*|__|__|/|__|__|/|__|__|__|__|	
☐1 Décision médicale<br>☐2 Auto-extubation	|__|__|/|__|__|/|__|__|__|__|	☐0 Oui ☐1 Non	☐1 *Identique* *Si non, date*|__|__|/|__|__|/|__|__|__|__|	
☐1 Décision médicale<br>☐2 Auto-extubation	|__|__|/|__|__|/|__|__|__|__|	☐0 Oui ☐1 Non	☐1 *Identique* *Si non, date*|__|__|/|__|__|/|__|__|__|__|	
☐1 Décision médicale<br>☐2 Auto-extubation	|__|__|/|__|__|/|__|__|__|__|	☐0 Oui ☐1 Non	☐1 *Identique* *Si non, date*|__|__|/|__|__|/|__|__|__|__|	
☐1 Décision médicale<br>☐2 Auto-extubation	|__|__|/|__|__|/|__|__|__|__|	☐0 Oui ☐1 Non	☐1 *Identique* *Si non, date*|__|__|/|__|__|/|__|__|__|__|	
☐1 Décision médicale<br>☐2 Auto-extubation	|__|__|/|__|__|/|__|__|__|__|	☐0 Oui ☐1 Non	☐1 *Identique* *Si non, date*|__|__|/|__|__|/|__|__|__|__|	
☐1 Décision médicale<br>☐2 Auto-extubation	|__|__|/|__|__|/|__|__|__|__|	☐0 Oui ☐1 Non	☐1 *Identique* *Si non, date*|__|__|/|__|__|/|__|__|__|__|	

Evénement indésirable

<table>
<tr><td>Code patient</td><td colspan="5"></td></tr>
<tr><td>Symptômes</td><td colspan="2"></td><td>Diagnostic</td><td colspan="2"></td></tr>
<tr><td>Intensité</td><td></td><td>Causalité</td><td></td><td>Si non relié à la recherche, lié à</td><td></td></tr>
<tr><td>Date de début</td><td></td><td>Evolution</td><td></td><td>Si séquelle(s), précisez</td><td></td></tr>
<tr><td>Date de fin</td><td></td><td>Date de décès</td><td colspan="3"></td></tr>
<tr><td>Action prise</td><td colspan="5"></td></tr>
<tr><td>EIG</td><td></td><td>Date à laquelle l’investigateur est informé de l’EIG</td><td></td><td>Poids du patient (Kg)</td><td></td></tr>
<tr><td>Date de déclaration initiale</td><td colspan="5"></td></tr>
<tr><td>Date de FU 1</td><td></td><td>Date de FU2</td><td></td><td>Date de FU3</td><td></td><td>Date de FU4</td><td></td></tr>
<tr><td>Gravité</td><td colspan="5"></td></tr>
<tr><td>Date de l’extubation</td><td></td><td>Date de sortie de réanimation</td><td colspan="3"></td></tr>
<tr><td rowspan="2">En cas d’hospitalisation ou de prolongation d’hospitalisation</td><td>Date de début d’hospitalisation</td><td></td><td>Procédure de l’étude</td><td>Date de début
Date de fin</td><td></td></tr>
<tr><td>Date de début de la gravité</td><td></td><td colspan="2">Date de fin d’hospitalisation</td><td></td></tr>
<tr><td colspan="6">Traitement concomitant avant la date d’EIG</td></tr>
<tr><td>Nom</td><td>Voie d’administration</td><td>Dose</td><td>Date début</td><td>Date de fin</td><td>Causalité</td></tr>
<tr><td></td><td></td><td></td><td></td><td></td><td></td></tr>
<tr><td></td><td></td><td></td><td></td><td></td><td></td></tr>
</table>

+ TEST MOCA
+ Questionnaire de santé SF-36
+ Questionnaire IES-R
+ Formulaire d’EIG
