# Supplementary material for: Open-label randomized controlled trial of ultra-low tidal ventilation without extracorporeal circulation in patients with COVID-19 pneumonia and moderate to severe ARDS: study protocol for the VT4COVID trial
Source: Trials. 2021 Oct 11;22:692. doi: 10.1186/s13063-021-05665-z (PMC8503716; doi:10.1186/s13063-021-05665-z)
Supplement: Supplementary file 7 — Additional file 7. TMoCA questionnaire (French version). [file 13063_2021_5665_MOESM7_ESM.pdf]

Version 3.0 5-Min MoCA French Adapted from Wong et al. by Dujardin et al.

MoCA ® – 5 min pour administration par téléphone – version française- v3

Apprentissage		Jambe	Coton	École	Tomate	Blanc	
Lire la liste de mots. Le patient doit répéter le plus possible de mots. Faire deux essais, même si le 1er est réussi. Faire un rappel à 5 minutes	1er essai						/5 1 point par mot correctement rappelé au 1er essai
	2ème essai						

Langage	/9
Nommer un maximum de mots commençant par la lettre « T » en 1 minute	≤2 = 0<br>]2,4] = 3<br>]4,8] = 6<br>>8 = 9

Orientation	[ ] Date	[ ] Mois	[ ] Année	[ ] Jour	[ ] Ville	[ ] Paris	/6

Demander :
Quelle est la date complète d'aujourd'hui ? Dans quelle ville êtes-vous actuellement ? Par rapport à Paris, cette ville se situe-t-elle au Nord, au Sud, à l'Est ou à l'Ouest ?
On donne un point par bonne réponse

Mémoire		Jambe	Coton	École	Tomate	Blanc	/10
Rappel des mots précédemment appris	Sans indice						- 2 points par mot rappelé sans indice<br>- 1 point par mot rappelé avec indice ou en choix multiple
	Indice catégoriel						
	Choix multiple						

Score	Ajouter un point si scolarité ≤ 12	/30

© Z. Nasreddine MD www.mocatest.org

Administré par : ______________________
