# Supplementary material for: Open-label randomized controlled trial of ultra-low tidal ventilation without extracorporeal circulation in patients with COVID-19 pneumonia and moderate to severe ARDS: study protocol for the VT4COVID trial
Source: Trials. 2021 Oct 11;22:692. doi: 10.1186/s13063-021-05665-z (PMC8503716; doi:10.1186/s13063-021-05665-z)
Supplement: Supplementary file 8 — Additional file 8. IES-R questionnaire (French version). [file 13063_2021_5665_MOESM8_ESM.docx]

| **IES-R version française**  ***Nom patient : Date passation :***  ***Instructions***.  Voici une liste de difficultés que les gens éprouvent parfois à la suite d’un événement stressant.  Veuillez lire chaque item et indiquer à quel point vous avez été bouleversé(e) par chacune de ces difficultés *au cours des 7 derniers jours* en ce qui concerne l’événement :  …………………………………………………………………………………………………………………………………… Dans quelle mesure avez-vous été affecté(e) ou bouleversé(e) par ces difficultés ? | | | | | |
| --- | --- | --- | --- | --- | --- |
|  | Pas du tout | Un peu | Moyen-nement | Passa-blement | Extrême-ment |
| 1. Tout rappel de l’événement ravivait mes sentiments face à l’événement | 0 | 1 | 2 | 3 | 4 |
| 2. Je me réveillais la nuit | 0 | 1 | 2 | 3 | 4 |
| 3. Différentes choses m’y faisait penser | 0 | 1 | 2 | 3 | 4 |
| 4. Je me sentais irritable et en colère | 0 | 1 | 2 | 3 | 4 |
| 5. Quand j’y repensais ou qu’on me le rappelait, j’évitais de me laisser bouleverser | 0 | 1 | 2 | 3 | 4 |
| 6. Sans le vouloir, j’y repensais | 0 | 1 | 2 | 3 | 4 |
| 7. J’ai eu l’impression que l’événement n’était jamais arrivé ou n’était pas réel | 0 | 1 | 2 | 3 | 4 |
| 8. Je me suis tenu loin de ce qui m’y faisait penser | 0 | 1 | 2 | 3 | 4 |
| 9. Des images de l’événement surgissaient dans ma tête | 0 | 1 | 2 | 3 | 4 |
| 10. J’étais nerveux (nerveuse) et je sursautais facilement | 0 | 1 | 2 | 3 | 4 |
| 11. J’essayais de ne pas y penser | 0 | 1 | 2 | 3 | 4 |
| 12. J’étais conscient(e) d’avoir encore beaucoup d’émotions à propos de l’événement, mais je n’y ai pas fait face | 0 | 1 | 2 | 3 | 4 |
| 13. Mes sentiments à propos de l’événement étaient comme figés | 0 | 1 | 2 | 3 | 4 |
| 14. Je me sentais et je réagissais comme si j’étais encore dans l’événement | 0 | 1 | 2 | 3 | 4 |
| 15. J’avais du mal à m’endormir | 0 | 1 | 2 | 3 | 4 |
| 16. J’ai ressenti des vagues de sentiments intenses à propos de l’événement | 0 | 1 | 2 | 3 | 4 |
| 17. J’ai essayé de l’effacer de ma mémoire | 0 | 1 | 2 | 3 | 4 |
| 18. J’avais du mal à me concentrer | 0 | 1 | 2 | 3 | 4 |
| 19. Ce qui me rappelait l’événement me causait des réactions physiques telles que des sueurs, des difficultés à respirer, des nausées ou des palpitations | 0 | 1 | 2 | 3 | 4 |
| 20. J’ai rêvé à l’événement | 0 | 1 | 2 | 3 | 4 |
| 21. J’étais aux aguets et sur mes gardes | 0 | 1 | 2 | 3 | 4 |
| 22. J’ai essayé de ne pas en parler | 0 | 1 | 2 | 3 | 4 |

**Score**:

- un score au-dessus de 22 moins d’1 mois après l’évènement : indice pour un stress aigü (surveiller)
- score au dessus de 36 plus d’1 mois après l’événement : indice pour un état de stress post-traumatique (consulter)
