# Supplementary material for: Open-label randomized controlled trial of ultra-low tidal ventilation without extracorporeal circulation in patients with COVID-19 pneumonia and moderate to severe ARDS: study protocol for the VT4COVID trial
Source: Trials. 2021 Oct 11;22:692. doi: 10.1186/s13063-021-05665-z (PMC8503716; doi:10.1186/s13063-021-05665-z)
Supplement: Supplementary file 9 — Additional file 9. SF36 questionnaire (French version). [file 13063_2021_5665_MOESM9_ESM.docx]

**Questionnaire de santé SF-36**

1. **Dans l’ensemble, pensez-vous que votre santé est** : (entourez la bonne réponse)

| Excellente | 1 |
| --- | --- |
| Très bonne | 2 |
| Bonne | 3 |
| Médiocre | 4 |
| Mauvaise | 5 |

2. **Par rapport à l’année dernière à la même époque, comment trouvez-vous votre état de santé en ce moment** ? (entourez la réponse de votre choix)

| Bien meilleur que l’an dernier | 1 |
| --- | --- |
| Plutôt meilleur | 2 |
| A peu près pareil | 3 |
| Plutôt moins bon | 4 |
| Beaucoup moins bon | 5 |

**3. Au cours de ces 4 dernières semaines, et en raison de votre état physique** (Entourez la réponse de votre choix, une par ligne)

| Liste de question | Oui | Non |
| --- | --- | --- |
| a. Avez-vous réduit le temps passé à votre travail ou à vos activités habituelles | 1 | 2 |
| b. Avez-vous accompli moins de choses que vous auriez souhaité ? | 1 | 2 |
| c. Avez-vous du arrêter de faire certaines choses ? | 1 | 2 |
| d. Avez-vous eu des difficultés à faire votre travail ou toute autre activité ? (par exemple, cela vous a demandé un effort supplémentaire) | 1 | 2 |

**4. Au cours de ces 4 dernières semaines, et en raison de votre état émotionnel (comme vous sentir triste, nerveux(se) ou déprimé(e))** (Entourez la réponse de votre choix, une par ligne)

| Liste de question | Oui | Non |
| --- | --- | --- |
| a. Avez-vous réduit le temps passé à votre travail ou à vos activités habituelles | 1 | 2 |
| b. Avez-vous accompli moins de choses que vous auriez souhaité ? | 1 | 2 |
| c. Avez-vous eu des difficultés à faire votre travail ou toute autre activité ? (par exemple, cela vous a demandé un effort supplémentaire) | 1 | 2 |

**5. Au cours de ces 4 dernières semaines dans quelle mesure votre état de santé, physique ou émotionnel, vous a-t-il gênée dans votre vie sociale et vos relations avec les autres, votre famille, vos connaissances** (Entourez la réponse de votre choix)

| Pas du tout | 1 |
| --- | --- |
| Un petit peu | 2 |
| Moyennement | 3 |
| Beaucoup | 4 |
| Enormément | 5 |

6. **Au cours de ces 4 dernières semaines, quelle a été l’intensité de vos douleurs (physiques) ?** (Entourez la réponse de votre choix)

| Nulle | 1 |
| --- | --- |
| Très faible | 2 |
| Faible | 3 |
| Moyenne | 4 |
| Grande | 5 |
| Très grande | 6 |

7. **Au cours de ces 4 dernières semaines, dans quelle mesure vos douleurs physiques vous ont-elles limité(e) dans votre travail ou vos activités domestiques ?** (Entourez la réponse de votre choix)

| Pas du tout | 1 |
| --- | --- |
| Un petit peu | 2 |
| Moyennement | 3 |
| Beaucoup | 4 |
| Enormément | 5 |

8. **Au cours de ces 4 dernières semaines ; y a-t-il eu des moments où votre état de santé ou émotionnel, vous a gêné dans votre vie et vos relations avec les autres, votre famille, vos amis, vos connaissances ?** (Entourez la réponse de votre choix)

| En permanence | 1 |
| --- | --- |
| Une bonne partie du temps | 2 |
| De temps en temps | 3 |
| Rarement | 4 |
| Jamais | 5 |

9. **Voici une liste d’activités que vous pouvez avoir à faire dans votre vie de tous les jours. Pour chacune d’entre elles indiquez si vous êtes limité(e) en raison de votre état de santé actuel.** (Entourez la réponse de votre choix, une par ligne)

| Liste d’activités | Oui, beaucoup limité(e) | Oui, un peu  limité(e) | Non, pas du tout limité(e) |
| --- | --- | --- | --- |
| a. Efforts physiques importants tels que courir, soulever un objet lourd, faire du sport | 1 | 2 | 3 |
| b. Efforts physiques modérés tels que déplacer une table, passer l’aspirateur, jouer aux boules | 1 | 2 | 3 |
| c. Soulever et porter des courses | 1 | 2 | 3 |
| d. Monter plusieurs étages par l’escalier | 1 | 2 | 3 |
| e. Monter un étage par escalier | 1 | 2 | 3 |
| f. se pencher en avant, se mettre à genoux, s’accroupir | 1 | 2 | 3 |
| g. Marcher plus d’un km à pied | 1 | 2 | 3 |
| h. Marcher plusieurs centaines de mètres | 1 | 2 | 3 |
| i. Marcher une centaine de mètres | 1 | 2 | 3 |
| j. Prendre un bain, une douche ou s’habiller | 1 | 2 | 3 |

**10. Les questions qui suivent portent sur comment vous vous êtes senti(e) au cours de ces 4 dernières semaines. Pour chaque question, veuillez indiquer la réponse qui vous semble la plus appropriée. Au cours de ces 4 dernières semaines, y a-t-il eu des moments où :**

(Entourez la réponse de votre choix, une par ligne)

| Liste d’activités | En permanence | Très souvent | Souvent | Quelques fois | Rarement | Jamais | |  |
| --- | --- | --- | --- | --- | --- | --- | --- | --- |
| a. vous vous êtes senti(e) dynamique ? | 1 | 2 | 3 | 4 | 5 | 6 | |  |
| b. vous vous êtes senti(e) très nerveux(se) ? | 1 | 2 | 3 | 4 | 5 | 6 | |  |
| c. Vous vous êtes senti(e) si découragé(e) que rien ne pouvait vous remonter le moral | 1 | 2 | 3 | 4 | 5 | 6 | |  |
| d. vous vous êtes senti(e) calme et détendu(e) ? | 1 | 2 | 3 | 4 | 5 | 6 | |  |
| e. vous vous êtes senti(e) débordant(e) d’énergie ? | 1 | 2 | 3 | 4 | 5 | 6 | |  |
| f. vous vous êtes senti(e) triste et abattu(e) ? | 1 | 2 | 3 | 4 | 5 | 6 | |  |
| g. vous vous êtes senti(e) épuisé(e) ? | 1 | 2 | 3 | 4 | 5 | | 6 | |
| h. vous vous êtes senti(e) heureux(e) | 1 | 2 | 3 | 4 | 5 | 6 | |  |
| i. vous vous êtes senti(e) fatigué(e) ? | 1 | 2 | 3 | 4 | 5 | 6 | |  |

**11. Indiquez pour chacune des phrases suivantes dans quelle mesure elles sont vraies ou fausses dans votre cas :** (Entourez la réponse de votre choix, une par ligne)

| Liste d’activités | Totalement vrai | Plutôt vrai | Je ne sais pas | Plutôt fausse | Totalement fausse |
| --- | --- | --- | --- | --- | --- |
| a. je tombe malade plus facilement que les autres | 1 | 2 | 3 | 4 | 5 |
| b. je me porte aussi bien que n’importe qui | 1 | 2 | 3 | 4 | 5 |
| c. je m’attends à ce que ma santé se dégrade | 1 | 2 | 3 | 4 | 5 |
| d. je suis en excellente santé | 1 | 2 | 3 | 4 | 5 |
