# Supplementary material for: Open-label randomized controlled trial of ultra-low tidal ventilation without extracorporeal circulation in patients with COVID-19 pneumonia and moderate to severe ARDS: study protocol for the VT4COVID trial
Source: Trials. 2021 Oct 11;22:692. doi: 10.1186/s13063-021-05665-z (PMC8503716; doi:10.1186/s13063-021-05665-z)
Supplement: Supplementary file 10 — Additional file 10. DSMB charter. [file 13063_2021_5665_MOESM10_ESM.docx]

| **Titre du protocole :** | **Ventilation ultraprotectrice chez les patients avec pneumonie à COVID-19 et SDRA modérément sévère à sévère – Etude randomisée contrôlée en ouvert** |
| --- | --- |
| **Type de projet :** | **RIPH mentionnées au 1 Hors Produit de Santé (HPS)** |
| **Références du Protocole :** | Référence Promoteur: **69HCL20_0322**  **N° IDRCB : 2020-A00869-30** |
| **Investigateur Coordonnateur :** | **Pr YONIS Hodane** |
| **Promoteur :** | **Hospices Civils de Lyon**  BP 2251  3 Quai des Célestins  69229 LYON Cedex 02  FRANCE |
| **Version du document :** | V1.0 de **03/04/2020** |

# Introduction

La Charte définit la composition du Comité de Surveillance Indépendant (CSI), **son rôle et ses responsabilités**, ses objectifs, les relations avec les autres acteurs de l'essai et le calendrier des réunions. Elle fournit également les procédures permettant d’assurer la confidentialité, les modalités de communication et le respect des lignes directrices de **méthodologie et de** statistiques.

Responsabilités du CSI

- Protection des intérêts des participants à l'étude,
- Evaluation de l'innocuité et l'efficacité le cas échéant de toutes les procédures de l'étude
- Garantir le bon déroulement et la **bonne** conduite générale de l'étude.

Le CSI devra, avec les données transmises :

• revoir les données individuelles des participants ou les résultats des analyses d’un point de vue sécurité, mais aussi efficacité (si requis par le protocole et les membres du CSI) afin de continuellement évaluer la balance bénéfice/risque de l’étude.

• surveiller la conception de l'étude, notamment : les procédures mises en œuvre pour la gestion des événements indésirables pour maximiser la sécurité des participants à l'étude et minimiser les risques; et aussi les données permettant le bon fonctionnement du CSI.

• évaluer l’état d’avancement de l'étude en évaluant périodiquement la balance bénéfice/risque des patients et d'autres facteurs qui peuvent influer sur les résultats d'études ;

• tenir compte de facteurs externes à l'étude, tels que des développements scientifiques ou thérapeutiques qui peuvent avoir un impact sur la sécurité des participants ou bien sur l'éthique de l'étude ;

• examiner la documentation concernant les événements indésirables graves et rapports de sécurité ;

• évaluer et signaler à l’investigateur coordonnateur et au promoteur tout problème pouvant impacter la bonne conduite de l'étude, le recrutement des participants, la taille de l'échantillon et / ou la collecte des données ;

• établir des recommandations sur la poursuite, la suspension, l’arrêt ou d'autres modifications concernant l'étude sur la base de l'expérience accumulée, y compris les effets observés, bénéfiques ou indésirables, du traitement à l'étude au promoteur de l’étude.

Ce comité est chargé d'identifier tout élément qui pourrait influer sur la protection des intérêts des participants à l'étude et la bonne conduite générale de l'étude.

# Organisation

## Composition

Le CSI est un groupe indépendant (*ne participant pas à l’étude, et ne travaillant pas aux HCL)*, consultatif, multidisciplinaire composé au minimum d'un premier expert médecin clinicien dans la pathologie étudiée, un deuxième expert de la pathologie étudiée ou un expert du produit ou de la procédure étudié (ex : pharmacologue/pharmacovigilant/matériovigilant) ou des mesures effectuées le cas échéant, un méthodologiste (ex : pharmacologue, biostatisticien …) qui, collectivement, ont de l'expérience dans la gestion et la conduite des essais cliniques.

Les membres du CSI ont été approuvés par le promoteur et l’investigateur coordonnateur. Les membres du CSI sont nommés pour la durée de l'essai clinique. Dans le cas où un membre est dans l'impossibilité de continuer à participer au CSI, le promoteur peut le remplacer selon l’avancement de l’étude et la pertinence. A noter que le CSI peut être force de proposition pour ce remplacement.

## Participation d’un biostatisticien

Idéalement, le biostatisticien présentant le rapport aux membres du CSI est un biostatisticien indépendant de l’étude. En cas d’impossibilité, le biostatisticien de l’étude produira l’information et le rapport de session fermée nécessaires aux membres du CSI. Le biostatisticien pourra participer aux sessions fermées du CSI afin de faciliter la présentation des résultats aux membres du CSI, mais en aucun cas il ne participera au vote n’étant pas membre du CSI.

## Conflits d'intérêts

Les membres du CSI doivent signer une déclaration de conflit d’intérêt (dernière page **de cette charte**) : ils n’ont obligatoirement pas de conflit d'intérêt significatif (de nature financière ou scientifique) avec l’étude. Ainsi, ni les investigateurs de l'étude, ni les personnes employées par le promoteur, ni les individus qui pourraient avoir des liens avec le produit ou la procédure à l’étude ne peuvent être un membre du CSI.

Les membres du CSI ne doivent pas posséder des actions de la société ayant son produit en cours d'évaluation par l'essai clinique. Tout membre du CSI qui développe d'importants conflits d'intérêts au cours de l'essai devra démissionner du CSI.

# Réunions du CSI

## Modalités des réunions

Le promoteur est responsable de l’organisation et la gestion des CSI. Il peut déléguer cette gestion au centre de coordination de l’étude qui sera alors le principal interlocuteur pour le CSI.

Toute autre personne en dehors du promoteur ou de son représentant impliquée dans l’étude ne devra pas communiquer directement avec les membres du CSI, au sujet de l’étude en dehors des réunions en ouvert.

La personne / le biostatisticien assurant les analyses et présentant le rapport aux membres du CSI sera responsable des échanges d’information avec le CSI. Il centralisera le rapport de session fermée (rapport statistique et vigilance) et sera le seul à transmettre ces informations (agrégées ou par bras, en « semi-levée d’aveugle » ou en ouvert) aux membres du CSI. L’intégrité de l’étude pourra ainsi être respectée.

Les réunions du CSI pourront avoir lieu physiquement ou par téléconférence et des mesures seront prises pour s'assurer que seules les personnes appropriées participent aux sessions ouvertes ou fermées. Si un membre du CSI ne peut être présent à une réunion comme initialement prévu, il en informe immédiatement le promoteur.

En l’absence d’un des membres lors de la réunion 0, le Président statuera de la validité de l’avis. En l’absence de Président nommé, les experts communiqueront entre eux (mail, conférence téléphonique ou autre) pour transmettre l’avis final.

La fréquence des réunions sera établie lors de la première réunion. La date, l’heure et le calendrier des réunions du CSI sont déterminés par accord mutuel entre les membres du CSI et le promoteur (ou centre de coordination).Des réunions supplémentaires peuvent être demandées par le CSI selon les informations qu’il reçoit au cours de l’étude, par le promoteur (ou comité directeur de l’étude, le cas échéant), en particulier dans le cas de :

- augmentation de la fréquence des effets indésirables attendus ou des effets indésirables graves;
- augmentation des effets indésirables graves inattendus.

Si l’investigateur coordonnateur souhaite solliciter le CSI pour une réunion exceptionnelle, il doit passer par le promoteur ou la structure responsable de la gestion du DSMB.

## Première réunion ou réunion 0 :

L'objectif de la première réunion du CSI est de:

- Rappeler le rôle d’un CSI et de ses membres, souligner l’importance du respect de l’intégrité des données
- Prendre connaissance de l’étude et éventuellement émettre des recommandations sur le protocole d'étude (design, objectifs et données recueillies…) et notamment les critères d’arrêt de l’étude et de traitement;
- Emettre des recommandations sur les données de sécurité et d’efficacité le cas échéant à surveiller particulièrement par le promoteur.
- Valider le format du rapport-type à fournir par le promoteur au CSI : notamment définir les données que souhaitent recevoir les membres du CSI et la nécessité de présenter les données en semi-levée d’aveugle…etc…
- Elire un Président (signataire de l’avis du CSI)
- Définir et valider la fréquence des réunions et les modalités de communication.

## Réunions de revue de données

Ces réunions se dérouleront en 2 sessions :

- Une session ouverte en présence des membres du CSI, du promoteur (du comité directeur de l’étude le cas échéant), de l’investigateur coordonnateur (ou représentant), de la coordination, du méthodologiste de l’étude, du biostatisticien assurant les analyses pour le CSI, de la vigilance et au besoin de tout autre intervenant dans l’étude (data manager, biologiste…). L’objectif de cette session est de faciliter les interactions, échanges entre les membres du CSI et le personnel de l’étude et leur fournir les informations pouvant impacter leur décision. Aucune donnée comparative (en aveugle ou non) ne devront être présentées ou abordées lors de cette session.
- Une session fermée restreinte aux seuls membres du CSI et au biostatisticien/personne en charge des analyses pour le CSI, non votant).

L’objectif de cette session est l’obtention d’un consensus et de recommandations des membres du CSI au promoteur de l’étude. Les membres du CSI ne devront pas divulguer d’informations comparatives concernant les patients de l’étude au personnel de l’étude lors de leurs recommandations.

# Les Rapports

Les rapports doivent être transmis aux membres du CSI au minimum **1 semaine** avant la date de la réunion.

## Contenu des rapports du CSI

On distingue deux rapports différents :

- un rapport de session ouverte incluant des données sur l’avancement de l’étude (bilan des inclusions, …), des données sur la qualité de l’étude (déviations, problèmes rencontrées, données manquantes sur les critères de jugement…etc…), des données administratives et réglementaires (amendements, résumé des monitorings…).

L’ensemble de ces informations sera transmis aux participants de la session ouverte avant la réunion du CSI par le promoteur ou le centre de coordination le cas échéant.

- un rapport de session fermée incluant une partie détaillée sur les données de vigilance (données de tolérance, bilan des EIG, EI le cas échéant …), le rapport statistique (données démographiques, données de sécurité et d’efficacité le cas échéant…)selon le format défini en réunion 0.

L’ensemble de ces informations (pouvant être agrégées ou par bras, en semi-levée d’aveugle ou en ouvert…etc…) sera transmis aux seuls membres du CSI avant la session fermée du CSI par le biostatisticien/personne en charge des analyses pour le CSI. Aucune autre personne impliquée dans l’étude ne devra avoir accès à ces données afin de préserver l’intégrité de l’étude et ses résultats.

En cas de nécessité, le CSI peut demander des informations complémentaires au promoteur.

Pour les essais contrôlés en double-aveugle, les données de sécurité seront présentés en semi-levée d’aveugle (bras A/bras B), en accord avec le statisticien, sauf si demande spécifique du CSI.

## Recommandations émises par le CSI

Après chaque réunion, le CSI fera des recommandations au promoteur sur la poursuite ou non de l’étude avec d’éventuelles modifications du protocole et sur la prise en charge des participants à l’étude.

Les recommandations seront envoyées au promoteur dans la semaine suivant la réunion par le président du CSI élu lors de la réunion 0.

L’avis du CSI est consultatif. A réception des recommandations du CSI, le promoteur (comité directeur de l’étude le cas échéant) prend sa décision et en informe le CSI si son avis diffère.

Le promoteur ou le centre de coordination transmettra à l’investigateur coordonnateur les recommandations du CSI et les décisions du promoteur si elles sont différentes.

## Compte-rendu

Le compte-rendu de session fermée : il est complémentaire aux recommandations mais peut comporter des éléments de discussion entre les membres, pouvant compromettre l’intégrité de l’étude. Ce compte-rendu devra donc rester confidentiel.

Si jugé nécessaire, le président du CSI devra rédiger un compte-rendu confidentiel décrivant les échanges de la session fermée. Ce compte-rendu confidentiel devra être conservé par le président du CSI pendant toute la durée de la recherche et adressé au promoteur en fin d’étude pour archivage, après analyse finale. Il doit rester consultable pendant toute la durée de l’étude en cas de demande des autorités et du promoteur.

**Tel Secrétariat :**

**……………………………….**

**Adresse email :**

**………………………………**

**Tel Portable :**

**……………………………….**

**Acceptation de la charte du CSI**

**Envoyer par fax au 04 72 11 51 90 ou à l’adresse** [**sa.drci.vigilance@chu-lyon.fr**](mailto:sa.drci.vigilance@chu-lyon.fr) **ce formulaire au promoteur ET les originaux par courrier postal au Siège administratif des HCL, DRCI, Vigilance des essais, 3 quai des célestins, 69002 LYON.**

Je, _________________________________ (indiquer **vos** nom **et prénom**), membre du Comité de Surveillance Indépendant (CSI) de l’étude **VT4COVID** suis d'accord avec les conditions énoncées dans la présente Charte **v1.0** du **03/04/2020**. Si la charte est modifiée de façon significative, tous les membres du CSI examineront les changements et doivent être d’accord avec la nouvelle Charte.

**Déclaration de conflit d’intérêt**

Je, __________________________________ (Indiquer vos nom et prénom), membre du CSI en tant qu’expert __________________________________ (Indiquer votre spécialité) de l’étude **VT4COVID** déclare être d’accord avec ce qui suit :

Je m'engage à:

- Protéger les intérêts et la sécurité des participants à l'étude;
- Préserver l'intégrité de l’étude
- D’être libre de tous préjugés et d’idées préconçues;
- Respecter les normes scientifiques et éthiques, et d'éliminer ou de divulguer, au cours de ma participation au projet de recherche clinique proposé, des conflits d'intérêts réels ou apparents.

En outre:

Je déclare que mon conjoint ou mes enfants à charge, mon employeur, ou moi-même n’avons aucun intérêt financier dans l’étude.

Je m'engage à ne pas interférer avec l'analyse ou la publication des résultats de l'étude.

Je m'engage à ne pas avoir un temps partiel ou plein, rémunéré ou non rémunéré dans toutes les organisations qui sont : (a) impliquées dans l'étude; (b) dont les produits seront utilisés ou testés dans l'étude considérée, ou dont les produits ou services seraient directement et de façon prévisible affectés de façon importante par les résultats de l'étude;

Je m'engage à ne pas être un dirigeant, membre, propriétaire, fiduciaire, administrateur, expert-conseil ou consultant de ces organisations;

**Confidentialité**

Je comprends que conformément à l’Article à l’article R. 5121-13 du Code de la santé publique, je m’engage à ne pas divulguer les informations scientifiques ou techniques de natures confidentielles appartenant au Promoteur (ci-après désignées les « informations confidentielles ») dont je pourrais avoir connaissance à lors de ma participation au CSI.

Je m’engage pendant toute la durée de l’Etude, et jusqu’au terme d’un délai de cinq (5) ans à compter de la fin de l’étude pour quelque cause que ce soit à :

- protéger, garder comme strictement confidentielles et à traiter avec un degré de précaution et de protection au moins égal à celui que j’ accorde à mes propres informations confidentielles de même importance l’ensemble des Informations Confidentielles dont j’aurai connaissance ;
- ne pas divulguer, soit directement soit indirectement, à tout tiers les Informations Confidentielles;
- ne pas utiliser, totalement ou partiellement les Informations Confidentielles dans un but autre que celui défini dans la présente charte sans le consentement préalable et écrit du promoteur;
- ne pas faire de demande de titre de propriété industrielle, ni à exercer un droit de propriété intellectuelle ou tout autre droit sur les Informations Confidentielles communiquées dans le cadre du présent CSI;
- ce que les Informations Confidentielles ne soient ni copiées, ni reproduites, ni dupliquées totalement ou partiellement lorsque de telles copies, reproductions ou duplications n'ont pas été autorisées par le promoteur.

Les obligations de confidentialité ne s'appliqueront qu'aux seules Informations Confidentielles qui auront été expressément présentées comme confidentielles par le promoteur au moment de leur divulgation, sous quelque forme que ce soit.

A cet effet, le promoteur qui transmettra une information confidentielle apposera la mention CONFIDENTIEL sur le support matériel des informations divulguées.

En l'absence de support matériel, Le promoteur s'engage à vous signaler expressément le caractère confidentiel de l'information et à vous confirmer ce caractère par écrit, dans le plus bref délai, et au plus tard dans les 30 jours de leur divulgation.

Toutes les Informations Confidentielles et leurs reproductions, transmises par la promoteur à votre attention resteront la propriété du promoteur et devront être restituées à ce dernier immédiatement sur sa demande.

Sauf tel que prévu ci-dessus, vous n’aurez aucune obligation et vous ne serez soumis à aucune restriction eu égard à toutes Informations Confidentielles dont vous pourrez apporter la preuve :

- qu'elles sont entrées dans le domaine public préalablement à leur divulgation ou après celle-ci mais dans ce cas en l'absence de toute faute qui vous soit imputable ; ou
- qu'elles vous sont déjà connues, cette connaissance préalable pouvant être démontrée par l'existence de documents appropriés dans vos dossiers ; ou
- qu'elles vous ont été transmises par un tiers de manière licite, sans restrictions, ni violation de la présente charte ; ou
- qu'elles ont été ou sont publiées sans contrevenir aux dispositions de la présente charte ; ou
- qu’elles n’ont pas été désignées ou confirmées comme Informations confidentielles conformément aux dispositions de la présente charte.

Il est expressément convenu que la divulgation et la fourniture par le promoteur d'Informations Confidentielles au titre de la présente Charte ne peut en aucun cas être interprétée comme vous conférant de manière expresse ou implicite un droit quelconque (aux termes d'une licence ou par tout autre moyen) sur les matières, les inventions ou les découvertes auxquelles se rapportent ces Informations Confidentielles.

Il en est de même en ce qui concerne les droits d'auteur ou autres droits attachés à la propriété littéraire et artistique, les marques de fabrique ou le secret des affaires.

En outre, il est convenu que le droit de propriété sur toutes les Informations Confidentielles que le promoteur vous divulgue au titre de la présente charte appartient en tout état de cause, sous réserve des droits des tiers, au promoteur.

Je vais recevoir des informations des Hospices Civils de Lyon, promoteur de l’étude y compris des informations exclusives et confidentielles.

Je comprends que je vais avoir accès à des dossiers dans le but de participer au CSI de l’étude.

Dans mon rôle de membre du CSI en tant que qu’expert je ne vais en aucun cas publier ou reproduire ces documents. Je reconnais également que je ne ferai aucune utilisation de ces documents, sauf dans le cadre de mes missions en tant membre du CSI de de l’étude.

Je vais prendre toutes les précautions pour empêcher l'accès de ces dossiers à toute autre personne. Je vais conserver toute la documentation confidentielle jusqu'à la fin de l'étude.

J'ai lu les termes de cet accord et je m’engage à les respecter.

______________________ ______________________ |___|___|-|___|___|-|___|___|___|___|

Signature Nom**, prénom**
