# Supplementary material for: Open-label randomized controlled trial of ultra-low tidal ventilation without extracorporeal circulation in patients with COVID-19 pneumonia and moderate to severe ARDS: study protocol for the VT4COVID trial
Source: Trials. 2021 Oct 11;22:692. doi: 10.1186/s13063-021-05665-z (PMC8503716; doi:10.1186/s13063-021-05665-z)
Supplement: Supplementary file 11 — Additional file 11. Funding document. [file 13063_2021_5665_MOESM11_ESM.pdf]

Hospices Civils de Lyon
votre santé,
notre engagement

# CERTIFICATE

I, Anne METZINGER –Deputy Director of the Clinical Research and Innovation Department, certify that the research project VT4-COVID:

*"Open label randomized controlled trial of ultraprotective ventilation without extracorporeal circulation in patients with COVID 19 pneumonia and moderate to severe ARDS",*

carried out by Professor Jean-Christophe RICHARD, is receiving financial support from the Ministry of Health within the framework of the hospital clinical research programm – PHRCI – COVID 2020. The total amount allocated is : 249 721,00 €

Lyon, 25th January 2021, for all legal intents and purposes.

**Deputy Director of Clinical Research and Innovation Department**

**Anne METZINGER**

3, quai des Célestins – 69002 Lyon – France B.P. 2251 – 69229 Lyon cedex 02
N° FINESS HCL : 690781810 | **www.chu-lyon.fr**
