# Supplementary material for: Open-label randomized controlled trial of ultra-low tidal ventilation without extracorporeal circulation in patients with COVID-19 pneumonia and moderate to severe ARDS: study protocol for the VT4COVID trial
Source: Trials. 2021 Oct 11;22:692. doi: 10.1186/s13063-021-05665-z (PMC8503716; doi:10.1186/s13063-021-05665-z)
Supplement: Supplementary file 12 — Additional file 12. Ethical approval document (French). [file 13063_2021_5665_MOESM12_ESM.pdf]

Comité de Protection des Personnes
Ile-de-France VII
BICETRE

Comité de Protection des Personnes Ile-de-France VII
CHU de BICETRE – 78 rue du Général Leclerc – 94275 LE KREMLIN BICETRE CEDEX

Bureau :
Présidente : Anne-Marie TABURET
Vice Présidente : Françoise BOISSY
Trésorier : Claude COTTET
Secrétaire : François HIRSCH

Secrétariat : Madame Brigitte PILATE-DAUSSY
(Portail des Champs – Secteur Bleu – Porte 74 bis – RDC)
e-mail : cpp.idf.7-bicetre@wanadoo.fr
site internet : http://cpp.idf.7.bicetre.free.fr
Téléphone : 01 45 21 28 46 – Télécopie : 01 45 21 21 45
Portable : 06 21 25 49 30

Premier Collège
Michel BOTTLAENDER Médecin investigateur (T)
François HIRSCH chercheur (T)
Paul de BOISSIEU Médecin de santé publique (T)
Gian Paolo DE FILIPPO pédiatre (T)
Renaud de BEAUREPAIRE Neurobiologiste (S)
Katia BOURDIC Technicienne d'étude clinique (S)
Catherine HILL Epidémiologiste (S)
Guillaume COINDARD Médecin généraliste (T)
Anne-Marie TABURET Pharmacien hospitalier (T)
Danièle BLONDELON Pharmacien hospitalier (S)

Deuxième Collège
Pascal CASAURANG Ethique (T)
Mireille COSQUER Psychologue (T)
Françoise BOISSY Juriste (T)
Valérie-Ann LAFOY Juriste (T)
Juliette GAUTIER Juriste (S)
Annie LABBE Représentant des associations (T)
Claude COTTET Représentant des associations (T)
Georges MARDUEL Représentant des associations (S)

Madame le Docteur Lucilla MANSUY
Hospices Civils de Lyon
BP 2251
3 quai des Célestins,
69229 LYON cedex 02

Kremlin Bicêtre le 14 avril 2020

Protocole N° 20-041
N° : 69HCL20_0322 - ETUDE VT4-COVID
ID-RCB n° 2020-A00869-30
(N° à rappeler dans toute correspondance)

Madame,

Le C.P.P. IDF VII a instruit en séance plénière (téléconférence) le 8 avril 2020 votre protocole intitulé :
ETUDE VT4-COVID : VENTILATION ULTRAPROTECTRICE CHEZ LES PATIENTS AVEC PNEUMONIE A COVID-19 ET SDRA MODEREMENT SEVERE A SEVERE – ETUDE RANDOMISEE CONTROLEE EN OUVERT. ETUDE RANDOMISEE CONTROLEE EN OUVERT."
dont l'investigateur principal est le Docteur Hodane YONIS - Service de Médecine Intensive Réanimation, - Service de Médecine Intensive Réanimation - Groupement Hospitalier Nord - Hôpital de la Croix Rousse - 103 Grande rue de la Croix Rousse - 69004 Lyon
et le promoteur est Hospices Civils de Lyon - BP 2251 - 3 quai des Célestins - 69229 LYON cedex 02

Recherche soumise en catégorie 1

Documents examinés :
- la lettre de soumisssion au CPP du 03/04/2020
- Le courrier AEC,
- Le formulaire de demande d'avis
- Le document additionnel
- le protocole d'étude (version 1 du 02/04/2020),
- le résumé en français (version 1 du 02/04/2020),
- la notice d'information consentement patient (version 1 du 02/04/2020),
- la notice d'information consentement représentant du patient (version 1 du
02/04/2020),
- la notice d'information consentement poursuite patient (version 1 du 02/04/2020),
- La Procédure d'urgence (version 1 du 02/04/2020),
- L'attestation d'assurance,
- La justification de l'adéquation des moyens mis en oeuvre,
- la liste des investigateurs (version 1 du 02/04/2020),
- le CV du Dr YONIS et de ses collaborateurs

Membres présents lors de la délibération de votre protocole

Premier Collège :
- Recherche biomédicale : Monsieur P. de BOISSIEU, épidémiologiste (T), Monsieur M. BOTTLAENDER (T), Monsieur F. HIRSCH (T), Monsieur G. P. de FILIPPO, pédiatre (T), Madame C. HILL (S), et Monsieur R. de BEAUREPAIRE (S)
- Pharmacien : Madame A. M. TABURET (T), Madame D BLONDELON (S)

Deuxième collège :
- Personne qualifiée en éthique : Monsieur P. CASAURANG (T)
- Psychologue : Madame M. COSQUER (T)
- Juriste : Madame F. BOISSY (T)
- Associations agréées : Madame A. LABBE (T) et Monsieur C. COTTET (T) et Monsieur G. MARDUEL (S)

Et a posé des questions en date du 9 et du 11 avril 2020

Les membres du CPP ont été contactés (échanges par courriel ou téléphone) les 11 et 14 avril 2020 pour pour examiner les réponses du 10 et du 13 avril 2020

Documents examinés :
- Lettre de réponse du 10/04/2020
- du protocole (version n°2 du 10/04/2020),
- du résumé (version n°2 du 10/04/2020),
- la notice d'information consentement patient (version n°2 du 10/04/2020),
- la notice d'information consentement représentant du patient (version n°2 du 10/04/2020),
- la notice d'information consentement poursuite patient (version n°2 du 10/04/2020),
- La Procédure d'urgence (version n°2 du 10/04/2020),

Et – lettre de réponse du 13/04/2020
- protocole (version n°3 du 12/04/2020),
- résumé (version n°3 du 12/04/2020),
- notice d'information consentement patient (version n°3 du 12/04/2020),
- notice d'information consentement représentant du patient (version n°3 du 12/04/2020),
- notice d'information consentement poursuite patient (version n°3 du 12/04/2020),

Le Comité :
considérant l'intérêt du projet de recherche
le respect d'une méthodologie adaptée à la question posée
considérant le respect d'un consentement libre et éclairé formulé au regard d'une note d'information adaptée

a adopté la délibération suivante :

AVIS FAVORABLE SANS RESTRICTION

Anne-Marie TABURET
Présidente du CPP IDF VII
