# Supplementary material for: Open-label randomized controlled trial of ultra-low tidal ventilation without extracorporeal circulation in patients with COVID-19 pneumonia and moderate to severe ARDS: study protocol for the VT4COVID trial
Source: Trials. 2021 Oct 11;22:692. doi: 10.1186/s13063-021-05665-z (PMC8503716; doi:10.1186/s13063-021-05665-z)
Supplement: Supplementary file 13 — Additional file 13. Ethical approval document (English). [file 13063_2021_5665_MOESM13_ESM.docx]

Comité de Protection des Personnes Ile-de-France VII

CHU de BICETRE – 78 rue du Général Leclerc – 94275 LE KREMLIN BICETRE CEDEX

Members : Secretariat : Madame Brigitte PILATE-DAUSSY

President : Anne-Marie TABURET (Portail des Champs – Secteur Bleu – Porte 74 bis – RDC)

Vice President : Françoise BOISSY e-mail : cpp.idf.7-bicetre@wanadoo.fr

Treasurer : Claude COTTET website : http://cpp.idf.7.bicetre.free.fr

Secretary : François HIRSCH Phone : 01 45 21 28 46 – Fax : 01 45 21 21 45

Portable : 06 21 25 49 30

1st College 2^nd^ College

Michel BOTTLAENDER MD (T) Pascal CASAURANG Ethics (T)

François HIRSCH researcher (T) Mireille COSQUER Psychologist (T)

Paul de BOISSIEU public health physician (T) Françoise BOISSY Jurist (T)

Gian Paolo DE FILIPPO pediatrician (T) Valérie-Ann LAFOY Jurist (T)

Renaud de BEAUREPAIRE Neurobiologist (S) Juliette GAUTIER Jurist (S)

Katia BOURDIC Clinical Research Assistant (S) Annie LABBE Associations representative (T)

Catherine HILL Epidemiologist (S) Claude COTTET Associations representative (T)

Guillaume COINDARD general practitioner (T) Georges MARDUEL Associations representative (S)

Anne-Marie TABURET hospital pharmacist (T)

Danièle BLONDELON hospital pharmacist (S)

Madam Lucilla MANSUY

Hospices Civils de Lyon

BP 2251

3 quai des Célestins,

69229 LYON cedex 02

Kremlin Bicêtre, avril 14^th^ 2020

Protocol N° 20-041

N° : 69HCL20_0322 - VT4-COVID study

ID-RCB n° 2020-A00869-30

(N° for all correspondence)

Madam,

The C.P.P. IDF VII has studied in plenary session (web conference) on April 8^th^ 2020 your study protocol entitled:

ETUDE VT4-COVID : VENTILATION ULTRAPROTECTRICE CHEZ LES PATIENTS AVEC PNEUMONIE A COVID-19 ET SDRA MODEREMENT SEVERE A SEVERE – ETUDE RANDOMISEE CONTROLEE EN OUVERT. ETUDE RANDOMISEE CONTROLEE EN OUVERT."

Whose principal investigator is Dr Hodane YONIS – Medical intensive care unit, Croix Rousse Hospital – 69004 LYON

And Whose sponsor is Hospices Civils de Lyon - BP 2251 - 3 quai des Célestins - 69229 LYON cedex 02

Research submitted as type 1 category.

Submitted documents :

- submission letter dated April 3^rd^ 2020

- clinical trial authorization letter

- request form

- additional document

- study protocol (version 1 dated April 2^nd^ 2020),

- French summary of the study protocol (version 1 dated April 2^nd^ 2020),

- patient inform consent (1st version dated April 2^nd^ 2020),

- authorized surrogate inform consent (1st version dated April 2^nd^ 2020),

- patient participation confirmation (1st version dated April 2^nd^ 2020),

- emergency inclusion procedure (1st version dated April 2^nd^ 2020),

- Insurance certificate,

- Investigators list (1st version dated April 2^nd^ 2020),

- Dr YONIS CV and her collaborators

Members in attendance during protocol deliberation

First College :

- Biomedical research : Mr P. de BOISSIEU, epidemiologist (T), Mister M. BOTTLAENDER (T), Mister F. HIRSCH (T), Mister G. P. de FILIPPO, pediatrician (T), Ms C. HILL (S), and Mr R. de BEAUREPAIRE (S)

- Pharmacist : Madame A. M. TABURET (T), Madame D BLONDELON (S)

Second college :

- Qualified person in ethics : Monsieur P. CASAURANG (T)

- Psychologist : Madame M. COSQUER (T)

- Jurist : Madame F. BOISSY (T)

- Authorized associations : Madame A. LABBE (T) and Monsieur C. COTTET (T) and Mr G. MARDUEL (S)

The Committee has asked for additional material on April 9^th^ and 11^th^ 2020

Ethics research committee members have been contacted (by mail or phone) on April 11^th^ and 14^th^ 2020 to examine answers from investigators dated April 11^th^ and 13^th^ 2020

Documents under review :

- letters from investigators dated April 10^th^ 2020 and April 13^th^ 2020

The Ethics research Committee, considering the interest of the research project, the adequacy of the methodology to the research question, the respect of informed consent and theadequacy of information to patients has stated the following deliberation:

FAVOURABLE RECOMMENDATION WITHOUT RESTRICTION

Anne-Marie TABURET

President of CPP IDF VII
