# Supplementary material for: Open-label randomized controlled trial of ultra-low tidal ventilation without extracorporeal circulation in patients with COVID-19 pneumonia and moderate to severe ARDS: study protocol for the VT4COVID trial
Source: Trials. 2021 Oct 11;22:692. doi: 10.1186/s13063-021-05665-z (PMC8503716; doi:10.1186/s13063-021-05665-z)
Supplement: Supplementary file 15 — Additional file 15. Consent form (surrogates). [file 13063_2021_5665_MOESM15_ESM.docx]

***Ventilation avec ultra faible volume courant chez les patients avec pneumonie à COVID-19 et SDRA modérément sévère à sévère – Etude randomisée contrôlée en ouvert***

***VT4-COVID***

***N° IDR-CB :*** ***2020-A00869-30***

***Version 2 du 10/04/2020***

**Promoteur** **(représenté par son représentant légal en exercice)** **:**

Hospices Civils de Lyon

BP 2251

3 quai des Célestins,

69229 LYON cedex 02

**Investigateur coordonnateur :** Dr Hodane YONIS

Service de Médecine Intensive Réanimation

Hôpital de la Croix-Rousse/Groupement Hospitalier Nord

103 Grande rue de la Croix Rousse 69004 Lyon

Téléphone : 04 72 07 17 62

*Madame, Monsieur,*

*Le médecin investigateur de votre proche vous a présenté l’étude VT4-COVID et sollicite votre accord pour qu’il (elle) y participe. Ce document a pour objectif de vous donner toutes les informations relatives à cette étude de façon à vous permettre d’exercer au mieux votre liberté de décision. Ce document est obligatoire et son contenu est défini par* [*le* ***Code de la Santé Publique, article L 1122-1***](http://www.legifrance.gouv.fr/WAspad/VisuArticleCode?commun=&code=&h0=CSANPUNL.rcv&h1=1&h3=11) *régissant les recherches impliquant la personne humaine.*

*Il décrit précisément l’étude et mentionne toutes les autorisations réglementaires obtenues pour sa mise en œuvre.*

*L’état de santé de votre proche lui empêche actuellement de prendre connaissance des informations contenues dans ce document et d’exprimer sa volonté de participer à l’étude. C’est pourquoi, en tant que membre de sa famille ou proche de confiance, le médecin de votre proche ou un investigateur sollicite votre accord.*

*Avant de prendre une décision, il est important que vous lisiez attentivement ces pages qui vous apporteront les informations nécessaires concernant les différents aspects de cette étude. Vous devez conserver ce document. N’hésitez pas à poser des questions si vous ne comprenez pas certains éléments.*

*La signature du formulaire de consentement, devenue obligatoire par l'application du code de la Santé Publique (livre I, titres 2 et 3 du CSP), n'affecte aucunement vos droits légaux, ni ceux de votre proche.*

*La participation de votre proche est entièrement volontaire. Si vous ne désirez pas qu’il (elle) prenne part à cette étude, il/elle continuera à bénéficier de la meilleure prise en charge médicale possible, conformément aux connaissances actuelles.*

**Pourquoi cette recherche?**

La pneumonie à COVID-19 est une maladie sévère, compliquée dans environ 25% des cas d’une insuffisance respiratoire aiguë sévère nécessitant l’admission en réanimation. En réanimation, plus de la moitié des patients vont nécessiter la ventilation artificielle et une anesthésie générale pour éviter une asphyxie. Ces patients sont dans un état extrêmement sévère avec un risque vital engagé chez environ la moitié d’entre eux.

La ventilation artificielle doit être réalisée le temps de la guérison de la pneumonie, c’est-à-dire pendant une quinzaine de jours. Cette ventilation peut sauver la vie du patient, et les réglages que l’on utilise actuellement sont qualifiés de protecteurs (par opposition aux réglages utilisés une vingtaine d’années auparavant dont on a démontré qu’ils nuisaient aux patients). La ventilation telle qu’elle est réalisée actuellement est donc qualifiée de protectrice. Toutefois, nous avons des arguments suggérant qu’elle puisse parfois encore être délétère, c’est-à-dire qu’elle peut aggraver les lésions pulmonaires faites par le virus.

Il est donc absolument nécessaire de trouver des solutions pour améliorer les modalités de réalisation de la ventilation artificielle.

**Quel est l’objectif de cette recherche?**

Votre proche est actuellement traité pour une infection pulmonaire liée au virus SARS-Cov2 (responsable de la maladie appelée COVID-19). Cette infection s’est compliquée d’une insuffisance respiratoire sévère (définie par l’abréviation SDRA pour syndrome de détresse respiratoire aiguë) à l’origine d’une dégradation importante de l’oxygénation. Le traitement du SDRA repose sur la ventilation artificielle pour améliorer l’oxygénation du sang, au moyen d’un respirateur artificiel. Les réglages de cette ventilation sont complexes, et il est possible que les modalités de ventilation artificielle qu’on utilise actuellement soient trop agressives sur le poumon, et que la réduction du volume d’air insufflé à chaque respiration (ou volume courant) permette de minimiser cette agression du poumon par la ventilation artificielle.

Une avancée potentielle est d’utiliser des réglages encore plus protecteurs, en diminuant le niveau d’assistance respiratoire pour mettre le poumon « au repos ». En effet, le poumon est déjà agressé par le virus, et la ventilation telle qu’elle est réalisée actuellement peut réaliser une 2^ème^ agression par le stress mécanique qu’elle impose au poumon. L’utilisation de réglages ultraprotecteurs (à très faible volume) devrait donc diminuer le stress imposé au poumon à chaque inspiration, en diminuant les variations de volume répétées à chaque inspiration (d’où le concept de mise au repos).

Cette stratégie ultraprotectrice s’accompagne toutefois d’une augmentation du gaz carbonique dans le sang, qui n’est pas ressentie par le patient car il est sous anesthésie générale. Les données actuelles sur cette stratégie ont été obtenues sur un petit nombre de patients. Il est maintenant absolument nécessaire de confirmer leur efficacité à plus grande échelle, afin de démontrer formellement leur effet potentiel sur la durée de la ventilation artificielle et la survie des patients atteints de pneumonie à COVID-19

L’objectif de l’étude dans laquelle nous souhaiterions inclure votre proche vise à évaluer l’intérêt de réaliser la ventilation avec un volume courant qui serait 30% plus faible que celui utilisé habituellement. Cette modalité de ventilation est appelée ultraprotectrice (par opposition à la ventilation habituelle qu’on considère protectrice). Pour réaliser cette évaluation et confirmer l’intérêt de cette thérapeutique innovante, il est nécessaire de réaliser un tirage au sort pour chaque patient, soit pour la réalisation d’une ventilation protectrice (prise en charge standard), soit pour la réalisation d’une ventilation ultraprotectrice (prise en charge innovante). Chaque patient a une chance sur 2 de se retrouver dans chaque groupe. C’est en effet le seul moyen dont on dispose pour conclure formellement à une supériorité éventuelle de la ventilation ultraprotectrice.

**Quelle est la méthodologie de cette recherche ?**

L’étude est réalisée dans une dizaine de centres français. La population susceptible d’être incluse dans l’étude est exclusivement les patients avec SDRA liée à une pneumonie à COVID-19, sous ventilation artificielle.

La durée d’application de la ventilation artificielle sera la plus courte possible en réanimation (une quinzaine de jour en moyenne) le temps d’attendre l’amélioration de l’état respiratoire de votre proche. Une fois la ventilation artificielle arrêtée, plus aucune procédure directement liée à l’étude ne sera réalisée, si ce n’est des questionnaires téléphoniques à 1 an suivant l’inclusion.

La durée de participation à l’étude est de 1 an, dans la mesure où un questionnaire téléphonique doit être réalisé à cette date pour que l’évaluation du traitement soit complète.

Le nombre total de personnes attendues pour participer à la recherche est de 200 patients.

En dehors des réglages sur le respirateur artificiel différents dans les 2 groupes de l’étude, le reste de la prise en charge sera conforme à l’ensemble des recommandations nationales et internationales sur la prise en charge du SDRA.

**Comment va se dérouler cette recherche ?**

Après inclusion dans l’étude, les réglages du respirateur artificiel vont être éventuellement modifiés et appliqués jusqu’à l’apparition des signes de guérison du SDRA (amélioration de l’oxygénation). Les réglages du ventilateur pourront être modifiés en fonction de l’évolution de certains paramètres mesurés sur les prises de sang au moins quotidiennement.

La participation à l’étude n’implique aucune souffrance supplémentaire, dans la mesure où les réglages ventilatoires testés ne seront appliqués que pendant la phase où votre proche sera sous anesthésie (traitement habituellement utilisé pour tolérer la ventilation mécanique à la phase aiguë du SDRA sévère).

Aucune prise de sang ne sera pratiquée spécifiquement pour l’étude.

Aucun traitement ne sera spécifiquement administré pour l’étude.

Aucun examen radiologique ne sera spécifiquement réalisé pour l’étude.

En revanche, des prises de sang et des examens biologiques seront régulièrement réalisés dans le cadre de la prise en charge habituelle d’un patient de réanimation.

La participation à l’étude comporte des visites supplémentaires à la prise en charge habituelle mais uniquement sous la forme de questionnaires téléphoniques sur la qualité de vie de votre proche, son état de stress et évaluant son fonctionnement cérébral 1 an après l’inclusion

L’inclusion dans l’étude nécessite des mesures de données cliniques et biologiques, qui se font dans le cadre du soin habituel aux patients avec insuffisance respiratoire aiguë grave. Nous souhaitons collecter pour les besoins de l’étude les données suivantes : données démographiques (âge, sexe, poids), données sur l’état de santé antérieur (antécédents médicaux, chirurgicaux, …), données sur l’histoire de la maladie (date de diagnostic, traitement, évolution), résultats des bilans biologiques habituellement pratiqués au cours de l'insuffisance respiratoire aiguë, données de mécanique respiratoire fournies par le respirateur (pression, volume, fréquence...), données cardiovasculaires (pression artérielle et veineuse, pouls, échographie cardiaque…), qui sont habituellement surveillées dans le cadre du soin.

Il existe des obligations ou restrictions imposées par la participation de votre proche à l’étude. Il lui sera impossible de participer à une étude interférant avec la stratégie ventilatoire testée (en pratique d’autres études sur la ventilation artificielle pendant le séjour en réanimation). Il lui sera aussi impossible de participer à une étude interférant avec l’évaluation de la présente étude. Enfin, il lui sera impossible de participer à une recherche impliquant la personne humaine de catégorie 1 dans les 90 jours suivant l’inclusion dans la présente étude.

Si vous décidez secondairement de retirer votre consentement ou si votre proche retire son consentement lorsqu’il sera en état de le faire, cela n’aura aucune implication pour sa prise en charge, et nous arrêterons de collecter des données dans le cadre de la recherche.

**Figure 1. Schéma de l’étude**

*
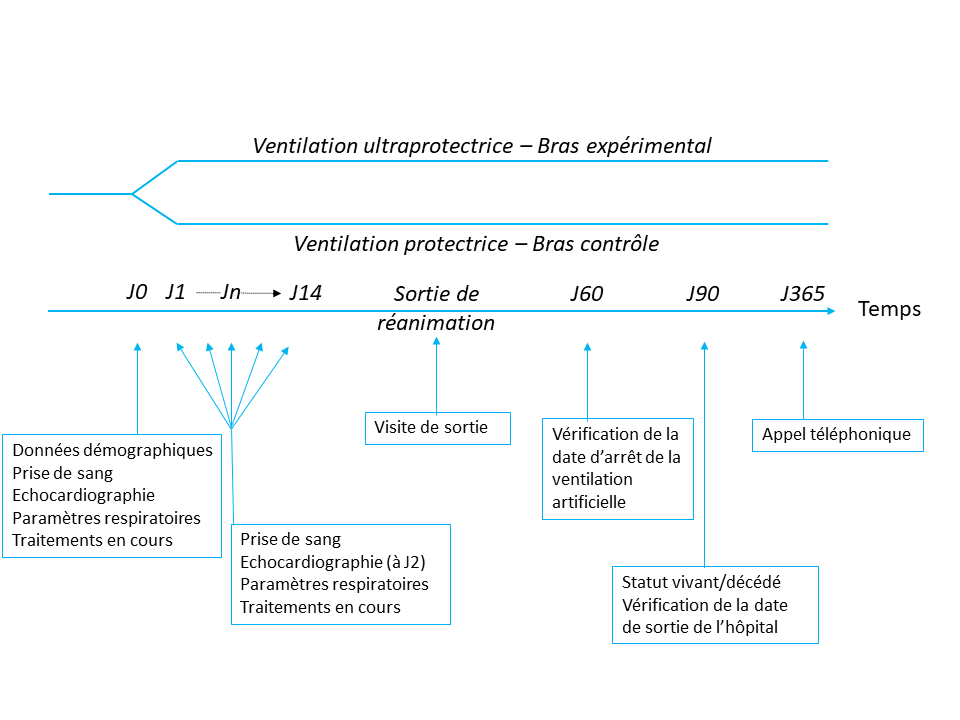
*

**Quels sont les bénéfices, les risques et les contraintes liés à la participation de votre proche ?**

Les bénéfices individuels potentiels associés à l’application de la stratégie testée sont une réduction de la durée de la ventilation mécanique, une sortie plus précoce de réanimation et un effet bénéfique sur la mortalité du SDRA.

Les bénéfices collectifs sont une réduction de la saturation des lits de réanimation dans le contexte épidémique, si la durée de ventilation et la durée de séjour en réanimation sont réduites par la ventilation ultraprotectrice.

Le seul risque associé à l’application de la stratégie testée identifié dans une étude pilote est une augmentation du gaz carbonique dans le sang en conséquence de la baisse du volume d’air insufflé par le respirateur. Ce risque sera contrôlé par un ajustement des réglages du respirateur, pour maintenir les paramètres biologiques à un niveau habituel.

Les contraintes liées à la participation à l’étude sont les suivantes : un appel téléphonique à J365 de l’inclusion pour les questionnaires de qualité de vie, d’évaluation des troubles du fonctionnement cérébral et du syndrome de stress post-traumatique. Cet appel durera environ 45 minutes

Il n’est pas attendu d’évènement indésirable lié au traitement à l’étude après la sortie de la réanimation. En cas de survenue d’un événement indésirable quel qu’il soit pendant l’hospitalisation en réanimation, celui-ci est signalé au promoteur de l’étude qui peut prendre la décision d’arrêter l’étude. Cet évènement indésirable sera pris en charge par l’équipe médicale.

**Quelles sont les éventuelles alternatives médicales ?**

Si vous décidez de ne pas faire participer votre proche à l’étude, il sera traité avec la ventilation protectrice, c’est-à-dire le groupe contrôle de l’étude, dans la mesure où il s’agit à ce jour du traitement de référence.

**Quels sont les traitements autorisés et non autorisés durant la recherche ?**

Il n’y a aucun traitement interdit pendant la durée de la recherche.

**Quels sont vos droits ?**

- - ***Participation libre et volontaire***

Vous êtes entièrement libre d’accepter ou de refuser que votre proche participe à cette recherche sans que cela ne modifie la qualité des soins auxquels il (elle) a droit, ou les relations existant avec son médecin ou l’investigateur.

Si vous décidez que votre proche participe à cette recherche, mais que vous changiez d'avis au cours de celle-ci, vous pouvez à tout moment demander d'interrompre sa participation à l’étude sans aucun préjudice, sans justification de votre part et sans que votre responsabilité ne soit engagée. Dans ce cas, les données de votre proche recueillies jusque-là seront utilisées dans les résultats de l’étude.

D'autre part, s'il le juge nécessaire pour le bien de votre proche, l’investigateur pourra modifier son suivi et il pourra continuer à bénéficier pleinement de sa compétence.

**Une fois que votre proche sera apte à comprendre et à exprimer sa volonté, nous solliciterons son accord écrit pour la poursuite de sa participation à l’étude *VT4-COVID*.**

Plus généralement, participer à cette étude ne décharge en aucune façon le représentant du promoteur ou les investigateurs de leurs devoirs envers votre proche.

A tout moment, toutes les informations que vous souhaiteriez obtenir ultérieurement concernant cette recherche vous seront communiquées dans la mesure du possible par le médecininvestigateur. Vous serez tenu informé(e) de toute nouvelle donnée importante concernant l’étude à laquelle vous acceptez que votre proche participe.

L’investigateur, tout comme le promoteur peut interrompre à tout moment la participation de votre proche à l'étude s’il juge que cela est dans son intérêt, ou arrêter l’étude dans sa globalité pour des raisons médicales, administratives ou autres.

Pour pouvoir participer à cette étude, votre proche doit nécessairement être affilié à un régime d’assurance maladie telle que celui de la sécurité sociale.

La participation de à cette étude n’empêche pas la participation de votre proche à une autre recherche.

Tous les frais médicaux liés à l’étude seront à la charge du promoteur. Il n’y aura pas de coût supplémentaire pour vous. Ni vous, ni votre proche ne serez rémunérés du fait de la participation à l’étude.

- - ***Confidentialités accès et protection des données***

Dans le cadre de la recherche interventionnelle à laquelle les Hospices Civils de Lyon proposent à votre proche de participer, un traitement informatique des données personnelles de votre proche va être mis en œuvre pour permettre d’analyser les résultats de la recherche au regard de l’objectif de cette dernière. Le responsable du traitement des données (qui est également responsable de la protection de vos données personnelles) est le promoteur, dont les coordonnées figurent sur la première page de ce document*.* Ce traitement des données a pour fondement juridique l’article 6 du Règlement Général sur la Protection des Données (RGPD) à savoir l’exécution d’une mission d’intérêt public dont est investi le responsable de traitement et les intérêts légitimes poursuivis par lui. De plus, au titre de l’article 9 du RGPD le responsable de traitement peut de manière exceptionnelle traiter des catégories particulières de données, incluant des données de santé notamment à des fins de recherche scientifique. Toutes vos données personnelles seront anonymisées (remplacées par un code).

La protection des données personnelles doit être assurée, conformément au Règlement Général Européen de la Protection des Données (RGPD) du 26 mars 2016, applicable au 25 mai 2018, et à la loi du 6 janvier 1978, modifiée, le 4 août 2004, dite « Loi Informatique et Liberté » et sa mise en cohérence avec la réglementation européenne par la loi du 20 juin 2018.

Pour l’analyse, les données médicales le/la concernant seront transmises aux Hospices Civils de Lyon ou aux personnes ou sociétés agissant pour son compte, en France ou à l’étranger. En cas de transfert de données à caractère personnel hors de l’Union Européenne et/ ou vers un pays ne garantissant pas un niveau de protection suffisant par rapport à l’Union Européenne ou à une organisation internationale, le promoteur et/ou le responsable de traitement mettront en place des garanties appropriées pour ce transfert (Clauses Contractuelles Spécifiques).

Si vous souhaitez obtenir une copie des Clauses Contractuelles Spécifiques, vous pouvez vous adresser au Délégué à la Protection des Données (DPO) du promoteur à l’adresse suivante : [dpo@chu-lyon.fr](mailto:dpo@chu-lyon.fr)**.**

Ces données seront identifiées par un code. Ces données pourront également, dans des conditions assurant leur confidentialité, être transmises aux autorités de santé françaises ou étrangères et à d’autres entités en dehors des Hospices Civils de Lyon.

Les seules personnes autorisées à consulter votre dossier médical sous sa forme nominative (c’est-à-dire directement identifiable) sont :

- votre médecin/le professionnel de santé qui vous suit et l’équipe soignante
- le personnel dédié aux recherches au sein de l'hôpital (appelées "Attachés Recherche Clinique ARC ou Techniciens d'Études Cliniques" TEC) pour saisir les données liées à la recherche ou assurer le contrôle des données
- le personnel du promoteur intervenant pour l’assurance qualité des données (appelé Attaché de Recherche Clinique) ou les autorités de santé

Sachez que toutes ces personnes sont soumises au secret professionnel.

Les données seront transférées et collectées conformément à la méthodologie de référence MR001 de la Commission Nationale de l’Informatique et des Libertés (CNIL) pour laquelle les Hospices Civils de Lyon ont signé un engagement de conformité. Conformément à la réglementation française et européenne, les données de l’étude seront conservées 25 ans.

Avec votre accord, le médecin traitant de votre proche sera informé de sa participation à l’étude.

Par ailleurs, sauf opposition expresse de votre part adressée à l’investigateur coordonnateur dont les coordonnées figurent sur la première page de ce document, les données de votre proche recueillies dans le cadre de cette étude pourront être transmises ailleurs dans le monde et réutilisées par des partenaires publics ou privés lors de recherches ultérieures exclusivement à des fins scientifiques.

Si vous avez des questions ou des réclamations au sujet du traitement des données de votre proche au cours de cette étude, vous pouvez contacter le DPO par voie électronique : [dpo@chu-lyon.fr](mailto:dpo@chu-lyon.fr)ou par courrier postal :

Le délégué à la protection des données

162 avenue Lacassagne

Bâtiment A – 3e étage – Bureau 316

69003 LYON

Si vous estimez, après avoir contacté le DPO des HCL, que vos droits sur les données de votre proche ne sont pas respectés, vous pouvez adresser une réclamation (plainte) à la CNIL (adresse postale : 3, Place Fontenoy - TSA 80715 – 75334 Paris CEDEX 07 ou courriel <https://www.cnil.fr/fr/webform/adresser-une-plainte>

- - ***Exercer vos droits***

Vous pourrez également, à tout moment, exercer votre droit d’accès, de vérification, de correction, de limitation, d’effacement et d’opposition au traitement et à la transmission des données concernant votre proche en faisant la demande auprès du médecin de votre choix ou auprès d’un investigateur de l’étude. Si vous souhaitez exercer votre droit à l’effacement des données de votre proche, le responsable de traitement peut au titre des Articles 17.3.c et 17.3.d. du RGPD ne pas faire droit à cette demande si celle-ci est susceptible de rendre impossible ou de compromettre gravement la réalisation des objectifs de la recherche. Ainsi, les données de votre proche recueillies préalablement au retrait de votre consentement pourront ne pas être effacées et pourront continuer à être traitées dans les conditions prévues par la recherche.

Si les résultats de cette étude devaient être présentés dans des communications et/ou des publications scientifiques médicales, l’identité des participants n’apparaîtra d’aucune façon.

Les données relatives à des effets indésirables qui seraient détectés dans le cadre de la présente étude doivent être collectées et traitées pour répondre à une obligation légale de vigilance et ne pourront donc donner lieu à une opposition de votre part.

A l’issue de l’étude, les résultats globaux de la recherche pourront vous être communiqués sur simple demande auprès de l’investigateur coordonnateur de l’étude, le Dr YONIS. Les données collectées dans le cadre de cette recherche peuvent être utilisées lors de nouvelles recherches conduites ultérieurement à des fins scientifiques. Vous pouvez décider de vous opposer à cette utilisation à tout moment auprès de votre investigateur.

- - ***Dispositions réglementaires***

L’étude sera conduite conformément aux lignes directives des Bonnes Pratiques Cliniques françaises et européennes, à la déclaration d’Helsinki dans sa dernière version, aux recommandations de l’ICH (International Conference on Harmonisation), Guideline for Good Clinical Practice ainsi qu’aux dispositions législatives et réglementaires en vigueur

Le Comité de Protection des Personnes *XX (Préciser le CPP tiré au sort et son adresse postale)* a émis un avis favorable à la réalisation de cette étude le *XX/XX/XXX (préciser la date d’avis favorable)*. L’Agence nationale de sécurité du médicament et des produits de santé (ANSM) a également donné son autorisation à la mise en œuvre de l’étude le *XX/XX/XXXX (préciser la date d’autorisation de l’étude)*. Enfin, cette recherche rempli les critères de conformité à la Méthodologie de Référence MR001 de la Commission Nationale de l’Informatique et des Libertés (CNIL) pour laquelle le promoteur a signé un engagement de conformité et respecte le règlement général sur la protection des données.

Le promoteur de cette recherche, les Hospices Civils de Lyon, BP 2251, quai des célestins, 69229 Lyon cedex 02, a souscrit une assurance de responsabilité civile auprès de la Société Hospitalière d’Assurance Mutuelle, 18 rue Edouard Rochet, 69008 Lyon, sous le numéro 159077.

Les personnes ayant subi un préjudice après participation à une recherche interventionnelle peuvent faire valoir leurs droits auprès de l’assureur du promoteur.

L’investigateur doit vous fournir toutes les explications nécessaires concernant cette recherche. Vous avez le droit d’arrêter la participation de votre proche à quelque moment que ce soit, et quel que soit le motif ; il/elle continuera à bénéficier du suivi médical et cela n'affectera en rien sa surveillance future.

**Qui pouvez-vous contacter pour toute question ?**

Si vous avez des questions concernant l’étude, n’hésitez pas à nous les poser. Nous pouvons vous donner les informations complémentaires que vous souhaitez. Les noms et numéros de téléphone des personnes à contacter sont les suivants :

Investigateur coordonnateur de l’étude

**Dr Hodane YONIS**, Service de Médecine Intensive Réanimation

Hôpital de la Croix-Rousse/Groupement Hospitalier Nord ; 103 Grande rue de la Croix Rousse 69004 Lyon

Téléphone : 04 72 07 17 62

Nous vous remercions de l’attention que vous avez portée à la lecture de cette notice. Une copie de ce document vous sera remise pour que vous puissiez bénéficier de l’ensemble des informations concernant la participation de votre proche à l’étude.

Lorsque vous aurez lu cette note d’information, il vous sera proposé, si vous êtes d’accord, de donner votre consentement écrit en signant le formulaire préparé à cet effet.

| 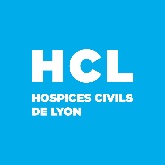  **Direction de la Recherche Clinique**  **et de l’Innovation** | **FORMULAIRE DE CONSENTEMENT A L’ATTENTION**  **DU REPRESENTANT DU PATIENT** |
| --- | --- |

**La loi 2012-300 du 5 mars 2012 relative aux recherches impliquant la personne humaine rend obligatoire le recueil de l'accord écrit des patients sollicités pour participer à toute recherche interventionnelle ou recherche interventionnelle à risques et contraintes minimes. C'est un tel accord qui vous est demandé ci-dessous, pour participer à l’étude intitulée :**

***Ventilation avec ultra faible volume courant chez les patients avec pneumonie à COVID-19 et SDRA modérément sévère à sévère – Etude randomisée contrôlée en ouvert***

***VT4-COVID***

***N° IDR-CB : 2020-A00869-30***

***Version 2 du 10/04/2020***

**Promoteur** **(représenté par son représentant légal en exercice) :**

Hospices Civils de Lyon

BP 2251

3 quai des Célestins,

69229 LYON cedex 02

**Investigateur coordonnateur :** Dr Hodane YONIS

Service de Médecine Intensive Réanimation

Hôpital de la Croix-Rousse/Groupement Hospitalier Nord

103 Grande rue de la Croix Rousse 69004 Lyon

Téléphone : 04 72 07 17 62

Je soussigné(e),……………….………………………………………………………………………………………………………………… *(NOM, Prénom)*, agissant en qualité de membre de la famille ou de proche de confiance de ………………………..………………………………………….……………….……………… *(NOM, Prénom)* certifie avoir lu et compris la note d’information qui m’a été remise.

J’ai eu la possibilité de poser toutes les questions que je souhaitais au Médecin investigateur qui m’a expliqué la nature, les objectifs, les risques potentiels et les contraintes liées à la participation de mon proche à cette recherche.

Je connais la possibilité qui m’est réservée d’interrompre la participation de mon proche à cette recherche à tout moment sans avoir à justifier ma décision et je ferai mon possible pour en informer l’investigateur qui suit mon proche dans la recherche. Cela ne remettra naturellement pas en cause la qualité des soins ultérieurs.

J’ai eu l’assurance que les décisions qui s’imposent pour la santé de mon proche seront prises à tout moment, conformément à l’état actuel des connaissances médicales.

J’ai bien compris que l’investigateur peut interrompre à tout moment la participation de mon proche à l’essai s’il le juge nécessaire.

Je suis informé(e) de la possibilité que les données de mon proche recueillies dans le cadre de cette étude puissent être réutilisées lors de recherches ultérieures exclusivement à des fins scientifiques et que je peux m’y opposer.

J’ai bien noté / été informé(e) que cette recherche a reçu l’avis favorable du Comité de Protection des Personnes de *nom du CPP* le *XX/XX/XXXX (préciser la date de l’avis favorable)* et l’autorisation de l’ANSM le *XX/XX/XXXX* *(préciser la date de l’autorisation de l’étude)* et a fait l’objet d’une déclaration à la Commission Nationale Informatique et Libertés (CNIL).

J’ai bien noté que cette recherche est menée conformément aux articles L1121-1 et suivants du Code de la Santé Publique, relatifs à la protection des personnes qui se prêtent à des recherches impliquant la personne humaine et conformément à la règlementation en vigueur.

Je certifie sur l’honneur que mon proche est affilié à un régime de sécurité sociale ou bénéficiaire d’un tel régime.

Le promoteur de la recherche, les Hospices civils de Lyon, BP 2251, quai des célestins, 69229 Lyon cedex 02 a souscrit une assurance de responsabilité civile en cas de préjudice auprès de de la Société Hospitalière d’Assurance Mutuelle, 18 rue Edouard Rochet, 69008 Lyon, sous le numéro 159077.

J’accepte que les personnes qui collaborent à cette recherche ou qui sont mandatées par le promoteur, ainsi qu’éventuellement le représentant des Autorités de Santé, aient accès à l’information contenue dans le dossier médical de mon proche dans le respect le plus strict de la confidentialité.

J’accepte que les données de mon proche enregistrées à l’occasion de cette recherche, puissent faire l’objet d’un traitement informatisé sous la responsabilité du promoteur.

J’ai bien noté que, conformément aux dispositions de la loi relative à l’informatique, aux fichiers et aux libertés, je dispose d’un droit d’accès, de rectification, de vérification, de correction, **de limitation, d’effacement** et d’opposition à la transmission des données de mon proche couvertes par le secret professionnel susceptibles d’être utilisées dans le cadre de cette recherche et d’être traitées. Ces droits s’exercent auprès de l’investigateur qui suit mon proche dans le cadre de cette recherche et qui connaît son identité.

Mon consentement ne décharge en rien l’investigateur et le promoteur de la recherche de leurs responsabilités à l’égard de mon proche. Mon proche et moi-même conservons tous les droits garantis par la loi.

Les résultats globaux de la recherche me seront communiqués directement, si j’en fais la demande, conformément à la loi du 4 mars 2002 relative aux droits des malades et à la qualité du système de santé. Je peux à tout moment demander des informations complémentaires au médecin investigateur.

Deux exemplaires originaux de ce formulaire de consentement ont été établis : un m’a été remis, l’autre a été remis à l’investigateur et sera conservé au minimum 25 ans après la fin de l’étude.

## Représentant du patient donnant son consentement :

## Ayant disposé d’un temps de réflexion suffisant avant de prendre ma décision, j’accepte librement et volontairement : - que mon proche …………………………………..……………………………………………….. *(NOM, Prénom)* participe à la recherche *VT4-COVID*

NOM, Prénom du représentant du patient : ……………………………………………………………………………………………………………………

Fait à : ………………………………………………………, le |___|___| / |___|___| / |___|___|___|___|

Signature du représentant du patient :

## Investigateur obtenant le consentement :

## J’atteste que toutes les obligations liées à un consentement éclairé ont été satisfaites dans le cadre de ce projet de recherche clinique – que le représentant du participant a reçu une information relative à ses droits, que nous avons discuté de ce projet et que je lui ai expliqué en termes compréhensibles l’ensemble des informations contenues dans la notice. Je certifie également avoir laissé le représentant du participant me poser toutes les questions qu’il souhaitait et y avoir répondu.

NOM, Prénom de l’investigateur : …………………………………………………………………………………………………………….

Fait à : ………………………………………………………, le |___|___| / |___|___| / |___|___|___|___|

Signature de l’investigateur :
